# From NO to NTe: *In Silico* Study of Ruthenium Compounds Containing Chalcogenonitrosyl Ligands

**DOI:** 10.1021/acsomega.5c08990

**Published:** 2025-11-25

**Authors:** Vinícius Glitz, Richard Fragnani Cardoso, Giovanni Finoto Caramori, Luis Henrique da Silveira Lacerda

**Affiliations:** Group of Molecular Electronic Structure and Materials, Departamento de Química, 28117 Universidade Federal de Santa Catarina, Campus Universitário Trindade, Florianópolis, SC 88040-900, Brasil

## Abstract

Ruthenium compounds bearing chalcogenonitrosyl ligands (NE, where E = O, S, Se, Te) represent a unique class of molecules with intriguing bonding patterns and potential relevance in redox-active systems. This manuscript presents a systematic investigation of a series of ruthenium-chalcogenonitrosyl compounds through combined Density Functional Theory (DFT) and generalized Kohn–Sham energy decomposition analysis (GKS-EDA) calculations. The compounds were evaluated both before and after one-electron reduction, focusing on their structural and electronic properties. Our results reveal clear trends in geometry, bond strength, and charge distribution, providing insight into the fundamental bonding interactions that govern the stability and redox behavior of these species. In particular, we examine the nature of the Ru–NE bond across the chalcogen series and evaluate the effects of one-electron reduction on these systems. The results demonstrate that one-electron reduction favors the labilization of the chalcogenonitrosyl ligands, as demonstrated by spin density plots and wave function analyses. The total interaction energy (Δ*E* ^tot^) for the Ru–NE bond indicates that, following one-electron reduction, this interaction is weakened by a factor of 2.9 to 4.2 (from NTe to NO) compared to the neutral species, accompanied by a decrease in the Ru–N–E angle of approximately 30°.

## Graphical abstract

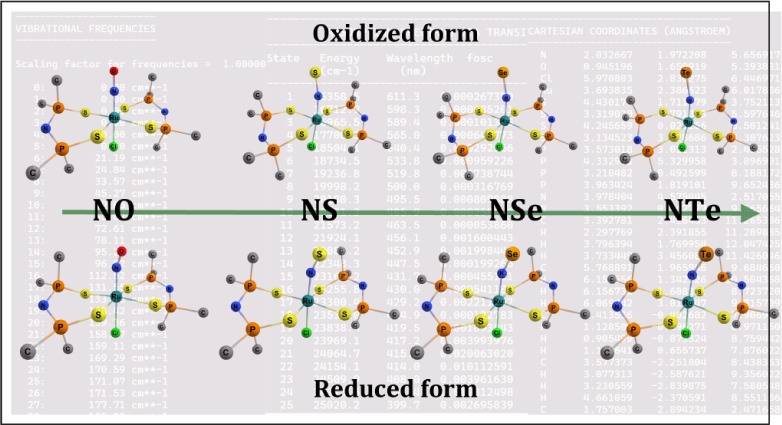

## Introduction

Nitric oxide (NO) has long been a subject of intense study, owing to its crucial role in biological systems, where it participates in processes such as cellular signaling and vascular regulation.^1,2^ In contrast, significantly less is known about its heavier congeners nitrogen sulfide (NS), nitrogen selenide (NSe), and nitrogen telluride (NTe), which are highly reactive and typically observed only under cryogenic or matrix-isolation conditions, with NTe presenting particular challenges due to its extreme reactivity and instability.^3–7^

Transition metal compounds of NO have therefore been widely investigated and well-characterized, both for their potential biological applications and for their versatility in various catalytic processes using NO.^8–11^ Significant contributions have been made over the years to elucidating the physical nature, through computational studies, of the various bonding situations present in ruthenium nitrosyl compounds.^12–16^ While the coordination of NS and NSe to transition metals has enabled some structural and spectroscopic studies,^17–19^ the heavier analogues, particularly NTe, remain largely unexplored.

Among the transition metals, ruthenium has proven particularly effective in stabilizing reactive ligands such as NO, NS, and NSe, owing to its flexible coordination chemistry and accessible redox states.^20^ Ruthenium compounds bearing these ligands have been successfully synthesized and characterized, providing valuable insights into the bonding, electronic structure, and reactivity of coordinated NO, as well as to support the stabilization of its heavier analogues, NS and NSe, through strong metal–ligand interactions.^21–23^ However, to date, there is an absence of structurally characterized ruthenium compounds containing the NTe ligand, likely due to the intrinsic instability of this species, which poses significant challenges for experimental isolation and characterization.

The redox behavior of ruthenium-nitrosyl compounds plays a crucial role in modulating their structural and electronic properties.^24^ In particular, the reduction of Ru–NO species has been shown to significantly impact the Ru–N–O bonding mode.^12,25^ Typically, the NO ligand in these compounds adopts a linear geometry with a bond angle close to 180°, consistent with a formal (NO)^+1^ configuration. Upon one-electron reduction, the added electron occupies the antibonding π* orbital of NO, generating a neutral NO^0^ species and a pronounced bending of the Ru–N–O angle.^12,26–28^

In contrast to the well-documented redox chemistry of Ru–NO compounds, the behavior of heavier nitrosyl chalcogenide analogues in reduced form remains poorly understood, mainly due to their limited experimental accessibility. The substitution of oxygen by sulfur, selenium, or tellurium introduces additional complexity since these ligands are inherently less stable and often require cryogenic conditions to persist. In this context, computational chemistry provides a powerful alternative for probing the structural and electronic consequences of chalcogen substitution in Ru–NE (E = O, S, Se, Te) systems.^22^ Through the application of theoretical studies, which include modeling reduction processes, analyzing bond situations, charge distributions, and orbital interactions, significant insights can be gained regarding the interaction of the Ru–NE bond and the release of NE.

This study presents a systematic computational investigation of ruthenium-chalcogenonitrosyl coordination compounds. The compounds prepared by Cheung and co-workers,^29^ containing dichalcogenoimidodiphosphinate ligands, have been used as prototypical to build the model structures used here. Furthermore, the effects of one-electron reduction of these systems are carefully assessed, yielding eight distinct models (Figure 1). These structures were analyzed using Density Functional Theory (DFT) to predict their geometries and electronic properties. In addition, generalized Kohn–Sham energy decomposition analysis (GKS-EDA) was performed to explore the physical nature of Ru–NE bonding situations.

**Figure 1:**
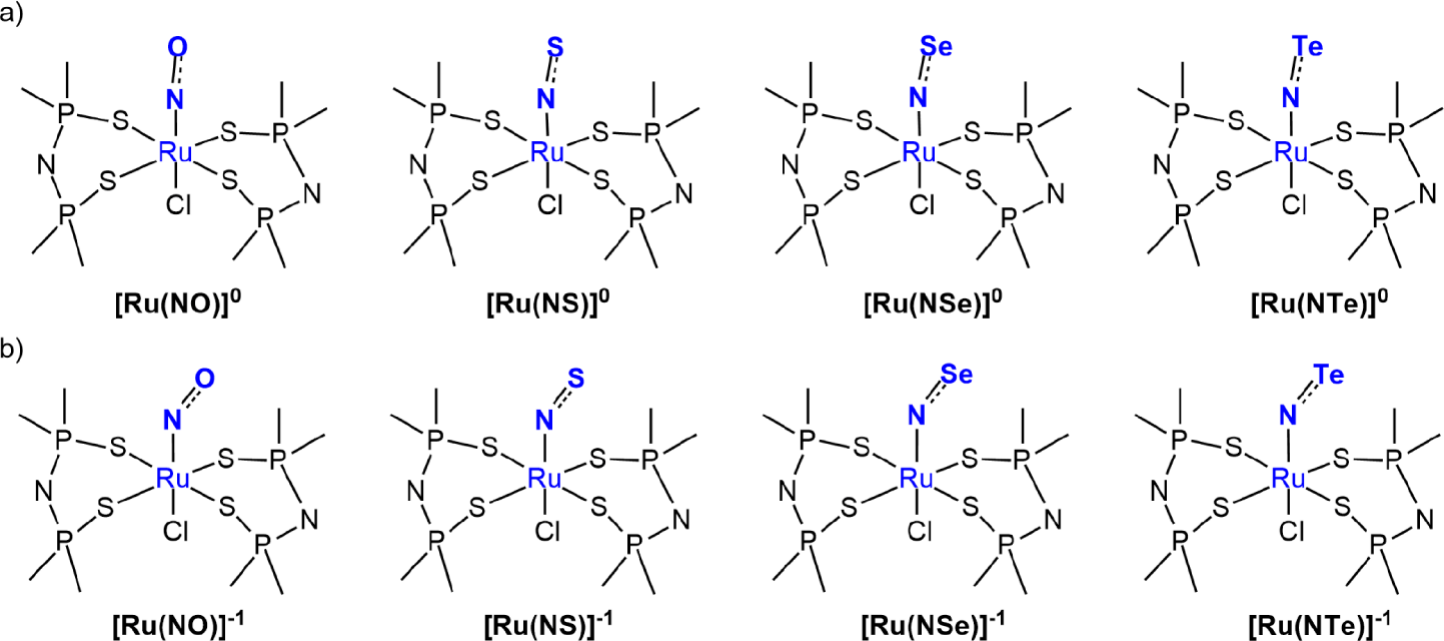
2D representations of the structures used in this work, (a) prior and (b) after one-electron reduction. The Ru–NE (E = O, S, Se, Te) bonds are highlighted.

In that sense, we present a thorough and systematic comparison of the physical nature of metal–ligand bonding situations of chalcogenonitrosyl with ruthenium, prior to and after the reduction of the NE groups. This comparison contributes to a deeper understanding of how reduction affects the bond strength in heavier Ru–NE analogues.

## Computational Methods

As previously stated in the introductory section, the reference structure employed was based on XRD data reported by Cheung et al. for the **[Ru­(NO)]** ^**0**^ compound (CCDC number: 632255).^29^ It has been utilized as a reference structure to construct the others examined in this study.

The Density Functional Theory calculations were performed using the generalized gradient approximation functional BP86, which combines the Becke exchange functional^30^ with the Perdew correlation functional.^31^ Grimme’s D3 dispersion correction^32^ with Becke-Johnson damping^33^ (BJ) was also applied. The sapporo-TZP-2012 basis set was employed for all atoms.^34–36^ Hessian matrices were analytically calculated for all optimized model structures to confirm the correct minimum on the potential energy surface, as evidenced by the absence of imaginary frequencies. The Hirshfeld charge, CHELPG charge, and Löwdin atomic charge and bond orders were obtained to assist in the structural analysis of the compounds. All calculations were performed using the ORCA package,^37^ version 5.0.3,^38^ and the images were rendered using the Chimera software^39^ or Chemcraft.^40^

Absorption spectra of all compounds were obtained using TD-DFT,^41^ by employing the same level of theory described above. To account for solvent effects in the absorption spectra, the geometries were reoptimized in the presence of the solvent using the SMD continuum solvation model with dichloromethane,^42^ and TD-DFT calculation were then performed on these solvent-optimized geometries as well as in the gas phase. The TheoDORE package (version 3.2) was then used to analyze the excited-state properties based on state and transition density matrices.^43^ This approach provides a unified framework for characterizing wave functions, enabling a detailed investigation of charge-transfer effects, excitonic interactions, and the nature of electronic transitions, regardless of the underlying wave function model.^44,45^

The level of theory applied was chosen based on an evaluation of different functionals (B3LYP,^46,47^ CAM-B3LYP,^48^ M06,^49^ M06L,^50^ PBE,^51^ PBE0,^52^ ωB97X,^53^ and BP86 ^30,31^ ) with various basis set (def2-SVP/TZVP/TZVP­(−f)/TZVPP and sapporo-(D/T/Q/)­ZP-2012)^34–36,54,55^ on the **[Ru­(NO)]** ^**0**^ structure. The obtained results demonstrate that the BP86-D3­(BJ)/sapporo-TZP-2012 level of theory provides the most accurate representation of the system. The reduced species was treated as an open-shell doublet, and the UHF spin contamination (*S* ^2^) showed a deviation of approximately 0.0045, indicating that spin contamination is negligible and the single-determinant description is reliable for further analysis.

Generalized Kohn–Sham energy decomposition analysis (GKS-EDA)^56^ was employed to gain insights into the [{Ru}–{NE}]^0/‑1^ bond through the total interaction energy (Δ*E* ^*tot*^) and its components. For these calculations, two fragments were considered: (i) the chalcogenonitrosyl ligand {NE}^1/0^, with E = O, S, Se, and Te; (ii) the rest of the molecule, represented solely as {Ru}. Known for its versatility, simplicity and robustness, GKS-EDA provides a powerful framework for dissecting chemical interactions.^57^ Its ability to quantify and decompose bonding contributions has been widely demonstrated, from covalent metal–ligand bonds in coordination complexes to noncovalent interactions, making it particularly suited to unravel the bonding trends observed in the present series.^58–61^

The GKS-EDA divides Δ*E* ^*tot*^ into physically meaningful terms, including electrostatic (Δ*E* ^*el*^), exchange (Δ*E* ^*ex*^), repulsion (Δ*E* ^*rep*^), polarization (Δ*E* ^*pol*^), correlation (Δ*E* ^*corr*^), and dispersion (Δ*E* ^*disp*^) contributions, as shown in eq 1. The exchange and repulsion terms can be combined, resulting in the Pauli repulsion (Δ*E* ^*exrep*^).^62^

1 $$\begin{array}{ll} \Delta E^{\mathrm{tot}}= & \Delta E^{\mathrm{el}}+\Delta E^{\mathrm{exrep}}\\ & +\Delta E^{\mathrm{pol}}+\Delta E^{\mathrm{corr}}+\Delta E^{\mathrm{disp}} \end{array}$$

The GKS-EDA results were obtained using the Xiamen Atomistic Computing Suite (XACS) and the GAMESS software.^63^ The analysis was performed using the B3LYP^30,64^ functional with Grimme’s D3 dispersion correction^32^ as the level of theory, in conjunction with the valence-only basis set SBKJC, which employs Stevens, Basch, Krauss, Jasien, and Cundari ECP potentials for all heavy atoms.^65–67^ For the one-electron reduced compounds, we employed an ROHF reference self-consistent field wave function.

## Results and Discussion

### Geometry and Structural Parameters

Initially, the neutral structures were optimized based on the structure reported by Cheung et al.,^29^ varying the nature of the chalcogen and replacing the isopropyl group bound to phosphorus with a methyl group. The data obtained for the main bond lengths are presented in Table 1. A close examination of the data reveals that the bond lengths of the Ru–N, Ru–Cl, and Ru–S bonds demonstrate minimal variability in response to variations in the type of chalcogen, E. The N–E bond length, where E = O, S, Se, and Te, has values of 1.164 Å, 1.529 Å, 1.686 Å, and 1.891 Å, respectively. Consequently, as the size of the chalcogen increases, the bond length of the chalcogenonitrosyl rises concomitantly.

**Table 1:** Main Bond Length (Å) and Angles (°) Values and Infrared Stretching Frequencies (*ν* in cm^–1^) Obtained at the BP86/Sapporo-TZP-2012 Level

	Ru–N	N–E	Ru–N–E	Ru–Cl	Ru–S^a^	ν(NE)
**[Ru(NO)]** ^**0**^	1.750	1.164	177.34	2.361	2.474	1826
**[Ru(NS)]** ^**0**^	1.760	1.529	170.35	2.379	2.463	1267
**[Ru(NSe)]** ^**0**^	1.751	1.686	169.40	2.386	2.463	1119
**[Ru(NTe)]** ^**0**^	1.748	1.891	170.35	2.397	2.460	1035
**[Ru(NO)]** ^**–1**^	1.817	1.201	142.36	2.852	2.515	1574
**[Ru(NS)]** ^**–1**^	1.834	1.574	143.87	2.497	2.461	1040
**[Ru(NSe)]** ^**–1**^	1.818	1.744	142.03	2.507	2.460	880
**[Ru(NTe)]** ^**–1**^	1.811	1.955	141.24	2.511	2.458	808

a Average Ru–S bond length.

It is noteworthy that when oxygen is part of the nitrosyl ligand, the Ru–N–E angle is 177°, whereas for the other chalcogens (S, Se, and Te), this angle is approximately 170°. This difference can be rationalized in terms of the atomic radii of the chalcogen atoms involved and the noncovalent interaction that they establish with the nearby hydrogen atoms in the methyl groups of equatorial ligand (Figure 2). In the case of **[Ru­(NO)]** ^**0**^, this distance is approximately 2.7 Å and 3.0 Å, whereas for S, Se, and Te, the shortest distance is 3.0 Å and the longest is 3.8 Å. It is well established that oxygen has the smallest van der Waals radius, (1.52 Å) followed by S (1.80 Å), then Se (1.90 Å), and finally Te (2.06 Å), which has the largest vdW radius among these four elements, but the last three have atomic radii that are closer to each other.^68^

**Figure 2:**
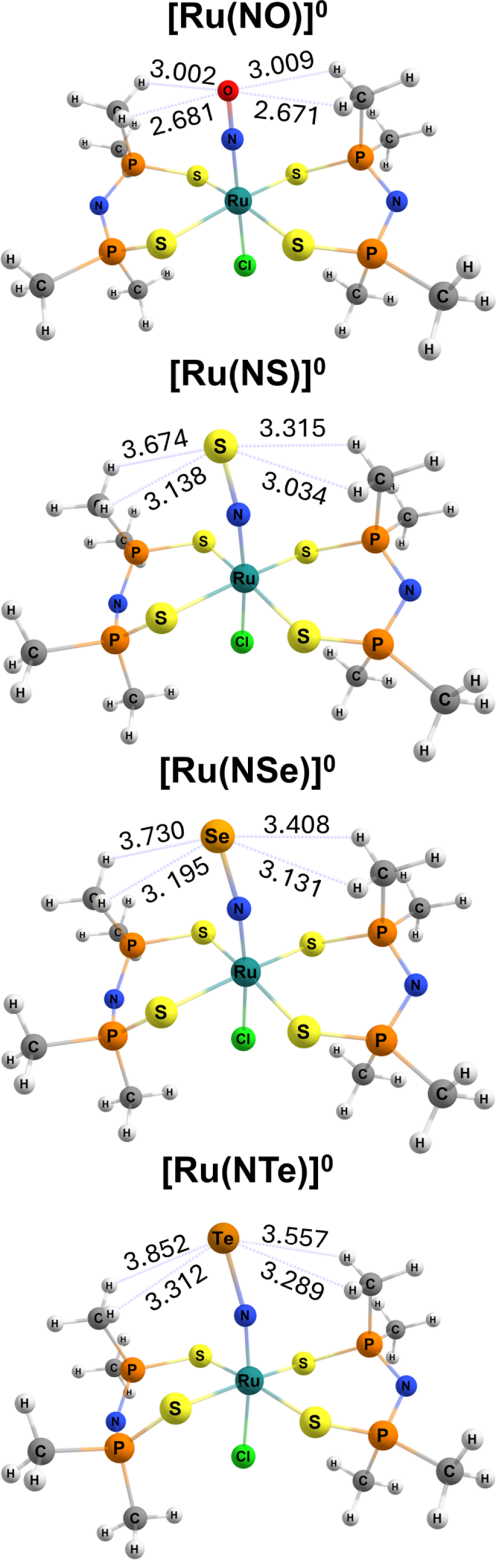
Distance in angstroms (Å) between the chalcogen and the hydrogen of the methyl groups.

In comparison with the structure reported by Cheung et al.,^29^ which has isopropyl groups bound to phosphorus, we observe very similar values between the XRD data and **[Ru­(NO)]** ^**0**^ structure, particularly for the Ru–N–O angle (177.3(5)° experimental and 177.34° calculated). Therefore, it is possible to state that the employed theoretical protocol has reproduced the experimental structural features with sufficient accuracy, as expected.

Ng et al. report a thionitrosyl compound with phenyl groups attached to phosphorus.^23^ Compared to the analogous **[Ru­(NS)]** ^**0**^ compound, the Ru–L bond lengths also exhibit similar values. However, the Ru–N–S angle reported is 177.0(6)°. The difference between our results (170.35°) and that reported by Ng et al. may be related to the presence of methyl and phenyl groups attached to phosphorus, which influence the Ru–N–E interaction, as previously demonstrated.

Comparing the experimentally reported stretching frequencies of ν­(NO) and ν­(NS) for the two compounds cited above (**[Ru­(NO)]** ^**0**^ and **[Ru­(NS)]** ^**0**^), our calculated results show good agreement with experiment, further confirming that the chosen level of theory is appropriate for describing the systems studied here. Cheung et al.^29^ measured ν­(NO) bands at 1834, 1841, and 1826 cm^–1^ (KBr) for Ru­(NO)­(L) complexes, while Ng et al.^23^ reported ν­(NS) bands at 1281, 1304, and 1305 cm^–1^ (KBr) for Ru­(NS)­(L) complexes. Our calculations yielded 1826 cm^–1^ for Ru­(NO) and 1267 cm^–1^ for Ru­(NS). It is worth noting that the phosphorus substituents in the experimental complexes differ from those in the models employed in our study, which can account for small variations in the predicted values.

For one-electron reduced compounds, the main difference is observed in the Ru–N–E angle, which decreases to approximately 142° for all four chalcogens, while Ru–Cl and Ru–N bond lengths increase; the Ru–S bond length remains unchanged. The observed phenomenon of bond bending upon one-electron reduction suggests a decrease in Pauli repulsion, as previously rationalized in other studies reported by our research group.^12^ In general, this behavior arises from the fact that the most available state for reduction is the Ru–NE π* orbital, which is unoccupied in neutral species but becomes occupied after the one-electron reduction. This bending reduces the Pauli repulsion stemming from Ru→N–E π-back-donation.

To confirm the site of reduction, the spin density of the reduced compounds was calculated (Figure 3). The results confirm that the spin density is localized on the Ru–N–E for all four chalcogens, suggesting that the reduction occurs in this region of the structure, which explains the decrease in the Ru–N–E angle in order to diminish the Pauli repulsion.

**Figure 3:**
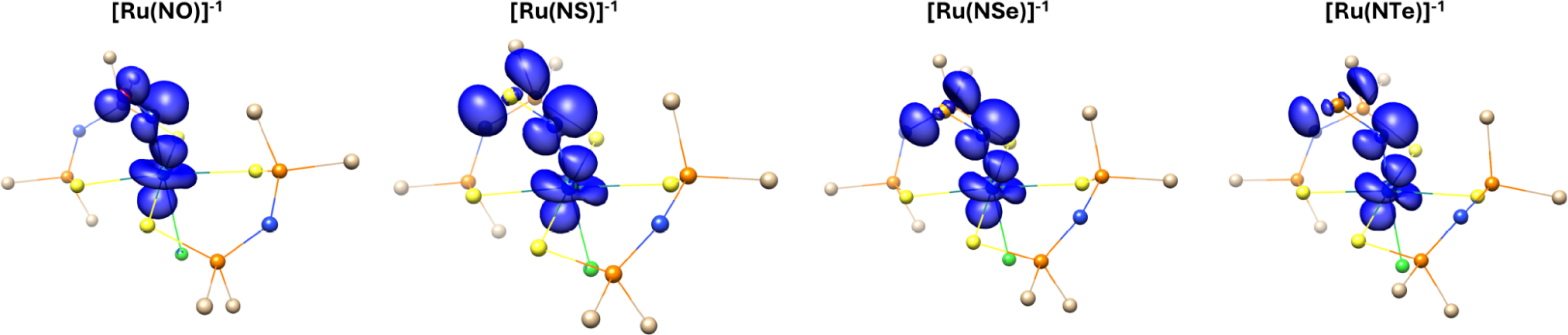
Spin density for reduced ruthenium compounds, in which the hydrogen atoms attached to carbon are omitted for sake of clarity. The isovalue used is 0.0067.

As a consequence, when compared with the free chalcogenonitrosyl in both cases, prior and after the one-electron reduction, (NE)^0^ and (NE)^−1^ respectively, an increase in the N–E bond length is observed when the group is coordinated to the ruthenium metal center. This change reflects a transition from a triple-bond character (free chalcogenonitrosyl) to a double-bond character (coordinated chalcogenonitrosyl), as shown in Tables 1, S1, and S3.

The analysis of the electronic structure of the chalcogenonitrosyl compounds was conducted by comparing the Hirshfeld, CHELPG, and Löwdin atomic charges, as well as the Löwdin bond orders, for the oxidized, reduced, and free NE species (Tables S1–S4). Additionally, the N–E stretching frequencies provide insights into the electronic effects induced by coordination and redox processes.

The Hirshfeld charges (Table S2) indicate that, in the neutral compounds (**[Ru­(NE)]** ^**0**^), the chalcogen atom exhibits a charge ranging from −0.02 to 0.12. This charge increases with the size of the chalcogen, from NO to NTe, respectively. This trend is also observed in the CHELPG and Löwdin charge analyses (Tables S3 and S4), where E becomes more electron-deficient in the neutral state. However, the chalcogen charge decreases after one-electron reduction, suggesting an increase in electron density on this atom. This behavior has been consistently observed across all three charge analysis methods.

Comparing the free NE species with the coordinated forms, the charge on nitrogen is significantly lower in the coordinated states, particularly in the neutral and reduced compounds. This indicates that the metal center plays a role in delocalizing charge over the Ru–N–E moiety, which is corroborated by the bond order analysis. The Löwdin bond orders for the N–E bond show a decrease upon coordination, consistent with the weakening of the bond from a triple bond (in the free NE) to a double bond character (in the coordinated NE). The bond order undergoes a further decrease upon reduction, thereby confirming the electronic influence of the additional electron.

The infrared stretching frequencies provide support for this interpretation, as evidenced by the higher stretching frequencies exhibited by the free chalcogenonitrosyl species in comparison to their coordinated counterparts (Tables S1 and 1). This observation indicates the presence of stronger N–E bonds. When coordinated, the N–E stretching frequencies decrease, reflecting the elongation and weakening of the N–E bond. Additionally, the stretching frequencies also decrease after one-electron reduction, confirming the increased electron density in the Ru–N–E moiety and the weakening of the N–E bond.

### Energy Decomposition Analysis

In order to shed light on the nature of Ru–NE bonding interactions, the generalized Kohn–Sham energy decomposition analysis (GKS-EDA) was performed on the neutral and reduced compounds employing a B3LYP/SBKJC level of theory. The results, presented in Table 2, demonstrate the predicted periodic trend in bond strengths, which is influenced by the nature of the chalcogen and the charge of the compound. The total interaction energy (Δ*E* ^*tot*^) is inversely proportional when comparing the neutral and reduced compounds, with a slope of −6.2 kcal mol^–1^ for [{Ru}–{NE}]^−1^ and 6.6 kcal mol^–1^ for [{Ru}–{NE}]^0^. Thus, **[Ru­(NO)]** ^**0**^ exhibits the most stabilizing total interaction energy among the neutral compounds (−248.46 kcal mol^–1^). Conversely, **[Ru­(NO)]** ^**–1**^ exhibits the least stabilizing energy among the reduced compounds, with a value of −59.25 kcal mol^–1^. This outcome is a direct consequence of the exchange-repulsion component, Δ*E* ^*exrep*^, which is only slightly diminished after the one-electron reduction. In contrast, the stabilizing components such as Δ*E* ^*ele*^, Δ*E* ^*pol*^, and Δ*E* ^*corr*^, exhibited a significant reduction, resulting in an inverted trend in the Ru–NE bond strengths to the reduced species compared to the nonreduced ones.

**Table 2:** Values of components for GKS-EDA (kcal mol^–1^), where the {Ru}^−^–{NE}^+^ and {Ru}^−^–{NE}^0^ bonds are decomposed

	Δ*E* ^*ele*^	Δ*E* ^*exrep*^	Δ*E* ^*pol*^	Δ*E* ^*corr*^	Δ*E* ^*disp*^	Δ*E* ^*tot*^
**[Ru(NO)]** ^**0**^	–104.77	161.43	–217.03	–83.62	–4.46	–248.46
**[Ru(NS)]** ^**0**^	–130.77	188.71	–215.31	–71.57	–7.93	–236.87
**[Ru(NSe)]** ^**0**^	–131.43	199.74	–227.76	–66.90	–8.87	–235.21
**[Ru(NTe)]** ^**0**^	–135.81	212.58	–231.90	–61.33	–10.41	–226.88
**[Ru(NO)]** ^**–1**^	–57.04	150.78	–73.81	–70.84	–8.35	–59.25
**[Ru(NS)]** ^**–1**^	–72.58	175.69	–102.65	–57.13	–9.11	–65.77
**[Ru(NSe)]** ^**–1**^	–73.47	188.63	–119.87	–56.64	–10.31	–71.66
**[Ru(NTe)]** ^**–1**^	–78.70	202.08	–134.15	–54.80	–12.52	–78.09

Furthermore, for the neutral compounds, the Δ*E* ^*tot*^ of the {Ru}–{NE} interaction is −248.46, −236.87, −235.21, and −226.88 kcal mol^–1^ for E = O, S, Se, and Te, respectively. For the reduced species, these values increase to −59.25, −65.77, −71.66, and −78.09 kcal mol^–1^ for E = O, S, Se, and Te, respectively. This destabilization is a consequence of the one-electron reduction, which weakens the Ru–NE interaction and consequently facilitates the labilization of the chalcogenonitrosyl group.

By examining the components contributing to Δ*E* ^*tot*^ (Table 2), it is observed that for the neutral compounds, the polarization values (Δ*E* ^*pol*^) make the largest contribution to stabilization (from −215 to −231 kcal mol^–1^), followed by electrostatic (Δ*E* ^*ele*^, from −104 to −135 kcal mol^–1^), correlation (Δ*E* ^*corr*^, from −61 to −83 kcal mol^–1^), and finally dispersion (Δ*E* ^*disp*^), which provides the smallest contribution (from −4 to −10 kcal mol^–1^). The Pauli repulsion (Δ*E* ^*exrep*^) has the largest value and increases as the size of the chalcogen increases, from 161 to 215 kcal mol^–1^.

The reduced compounds follow the same trend: the polarization term contribute from −73 to −134 kcal mol^–1^, electrostatic term from −57 to −78 kcal mol^–1^, correlation term from −54 to −70 kcal mol^–1^, and for the last, dispersion term contribute from −8 to −12 kcal mol^–1^. The Pauli repulsion term shows values similar to those of the neutral compounds, from 150 to 202 kcal mol^–1^.

It is interesting to note that **[Ru­(NO)]** ^**–1**^ stands out compared to the heavier congeners, once the correlation term contributes more significantly than the electrostatic term, with values of −70.84 and −57.04 kcal mol^–1^, respectively. For other reduced compounds, values ranging from −54 to −57 kcal mol^–1^ for Δ*E* ^*corr*^ and −72 to −78 kcal mol^–1^ for Δ*E* ^*ele*^ were found.

The observed trends in total interaction energy and its decomposition components reinforce the role of electronic effects in modulating bond strength and stability before and after one-electron reduction. Notably, the pronounced decrease in electrostatic and polarization contributions to Δ*E* ^*tot*^ upon reduction suggests a shift in the nature of the interaction. These findings corroborate the structural and energetic trends discussed earlier, supporting the idea that the reduction process weakens the Ru–NE bond and enhances the lability of the chalcogenonitrosyl ligand.

To verify the behavior of the physical components that constitute the total energy, a variation of the Ru–N–E angle in oxidized and reduced compounds between 120° and 180° has been performed. The values corresponding to the most stable angle for each compound, obtained via GKS-EDA, are presented in Figure 4 and the values depicted in Tables S5 - S12.

**Figure 4:**
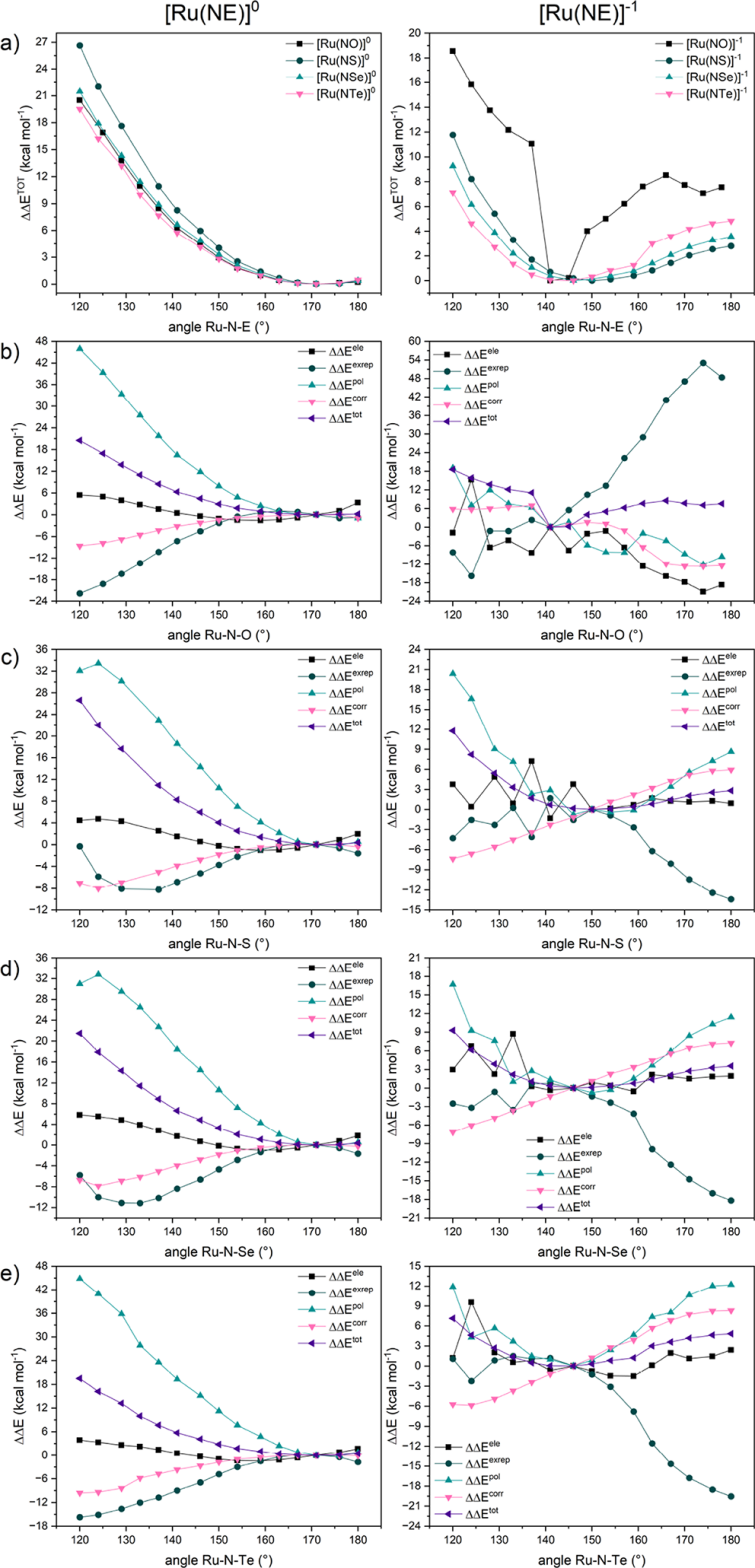
Variations in the GKS-EDA values (ΔΔ*E*) for all compounds, with oxidized species on the left and reduced species on the right. (a) Total energy variation for the four chalcogen atoms. Variation in the components relative to the angle with the lowest Δ*E* ^tot^ for [Ru­(NE)]^0/‑1^, where E = (b) oxygen, (c) sulfur, (d) selenium, and (e) tellurium.

Focusing first on the oxidized species, we observe that for all chalcogen atoms considered, as the Ru–N–E angle becomes more linear (approaching 180°), the value of ΔΔ*E* ^*tot*^ stabilizes near Ru–N–E angles of 170–180° (Figure 4a). The components ΔΔ*E* ^*ele*^, ΔΔ*E* ^*exrep*^, ΔΔ*E* ^*pol*^, and ΔΔ*E* ^*corr*^ for E = O, S, Se, and Te, are shown in Figure 4b-e, respectively, on the left side. It is evident that all components exhibit a uniform trend, irrespective of the chalcogen. The most significant variations occur for ΔΔ*E* ^*pol*^, which stabilizes, and for ΔΔ*E* ^*exrep*^ and ΔΔ*E* ^*corr*^, which become less stable as the Ru–N–E angle approaches linearity. ΔΔ*E* ^*ele*^ shows only a slight variation with angle, as does ΔΔ*E* ^*disp*^, as evidenced by the values presented in Tables S5 - S8 in the Supporting Information.

For the reduced species, the most stable total interaction energy for Ru–NE is found in the range of 140–150°, as seen in the ΔΔ*E* ^*tot*^ values in Figure 4a on the right. Regarding the components shown in Figure 4b-e for E = O, S, Se, and Te, respectively, ΔΔ*E* ^*pol*^ stabilizes and then destabilizes as the angle varies from 120° to 180°, while the opposite trend is observed for ΔΔ*E* ^*corr*^. For ΔΔ*E* ^*exrep*^, minor variations occur before the 140–150° range, followed by a stabilization beyond this region. The values of ΔΔ*E* ^*ele*^ and ΔΔ*E* ^*disp*^ exhibit slight variations with changes in the angle. All values are presented in Tables S9 - S12.

These results further support the influence of electronic effects on the Ru–NE bonding interactions, particularly in response to angular variations. The observed stabilization trends in ΔΔ*E* ^*tot*^ for both oxidation states highlight the structural preference of each species, with the oxidized compounds favoring a more linear geometry and the reduced species stabilizing at a more bent configuration. The distinct behaviors of ΔΔ*E* ^*pol*^, ΔΔ*E* ^*corr*^, and ΔΔ*E* ^*exrep*^ reinforce the idea that reduction not only weakens the Ru–NE bond but also alters the balance between attractive and repulsive forces, particularly diminishing the Pauli repulsion between the interacting fragments. Notably, the polarization and correlation terms play a crucial role in dictating the angular dependence of stability, particularly for the reduced compounds, where these components exhibit opposing trends.

### Wave Function Analysis

The frontier molecular orbitals were analyzed based on canonical Kohn–Sham orbitals obtained from TD-DFT calculations. Figure 5a presents the molecular orbital for **[Ru­(NO)]** ^**0**^ and **[Ru­(NO)]** ^**–1**^, with the remaining compounds exhibiting analogous behavior. For the oxidized species, the highest occupied molecular orbital (HOMO) is primarily composed of contributions from *d*_(Ru)_ and the bidentate ligand, through its *p*_(S)_ and *p*_(N)_ orbitals. The lowest unoccupied molecular orbital (LUMO) consists mainly of *d*_(Ru)_and $\pi_{\left(\mathrm{NO}\right)}^{*}$ orbitals. Figure 5 supports the conclusion that upon one-electron reduction of **[Ru­(NO)]** ^**0**^, the added electron is primarily localized on the NO moiety, where the LUMO is centered on the Ru–NE fragment.

**Figure 5:**
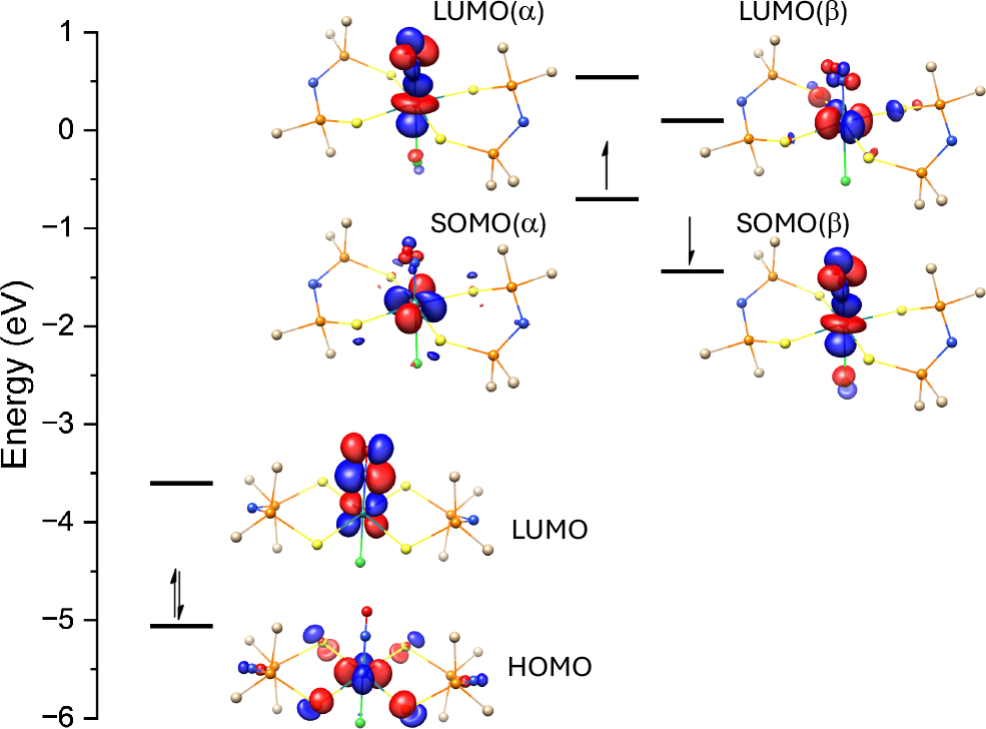
Frontier molecular orbitals for **[Ru­(NO)]** ^**0**^ and **[Ru­(NO)]** ^**–1**^. Hydrogens attached to carbon are omitted for clarity; an isovalue of 0.072 was assumed.

The reduced species displays two sets of orbitals, as it is an open-shell compound, as shown in Figure 5b. The alpha singly occupied molecular orbital (SOMO­(α)) is mainly localized on *d*_(Ru)_, with small contributions of *p*_(S)_ and $\pi_{\left(\mathrm{NO}\right)}^{*}$ , while the SOMO­(β) is composed of *d*_(Ru)_and $\pi_{\left(\mathrm{NO}\right)}^{*}$ orbitals. The LUMO­(α) show similar characteristics to SOMO­(β), whereas the LUMO­(β) is localized on *d*_(Ru)_, $\pi_{\left(\mathrm{NO}\right)}^{*}$ and *p*_(S)_ orbitals.

To complement the orbital analysis, Table S13 reports the frontier orbital energies (in eV) for both oxidized and reduced **[Ru­(NE)]** species (E = O, S, Se, Te). For the oxidized series, the HOMO levels remain relatively consistent across all ligands, indicating that the substitution of oxygen by heavier chalcogens does not significantly affect the highest occupied orbitals, which are largely metal-centered. In contrast, the LUMO levels show a progressive decrease in energy from NO to NTe (−3.10 to −3.71 eV), reflecting increased interaction between the metal center and the more polarizable chalcogenonitrosyl ligands.

For the reduced species, the SOMO­(β) levels are consistently more stabilized than SOMO­(α), as expected due to spin polarization effects. A comparison across the reduced series reveals a similar pattern of decreasing energy in the unoccupied orbitals, with the SUMOs of **[Ru­(NTe)]** ^**–1**^ exhibiting the lowest energy, suggesting increased electron affinity and potential reactivity compared to the lighter congeners.

To gain deeper insight into the orbital transitions and the effect of one-electron reduction, we performed a fragment-based excited-state analysis, as developed by Plasser and implemented in the TheoDORE package.^43^ This approach decomposes the one-particle transition density matrix (1TDM) to examine charge-resonance and excitonic correlation phenomena.^44,45^ The analysis focused on the graphical interpretation of excited-state character through electron–hole correlation plots derived from the Ω matrices, as well as the decomposition of the charge-transfer number matrices. In this context, the hole refers to the region of electron depletion (initial orbital), while the electron corresponds to the area of electron accumulation (final orbital) upon excitation.

These visual tools provided qualitative insights into the degree of charge separation and delocalization across different chalcogens and oxidation states. For all structures, prior to and after one-electron reduction, the first 25 singlet excited states were computed at the TD-DFT level, and the molecular system was divided into four fragments: (i) Ru, (ii) NE, (iii) Cl, and (iv) L. In this case, L refers to a both bidentate ligand.

As shown in Figures 6 and S1a - S3a, except for **[Ru­(NO)]** ^**0**^, all oxidized species exhibit low-energy electronic transitions below 2.0 eV. These correspond to transitions ranging from S_1_ to S_8_ for the **[Ru­(NS)]** ^**0**^ and **[Ru­(NSe)]** ^**0**^ compounds, and up to S_9_ for the **[Ru­(NTe)]** ^**0**^ compound. All remaining excited states, S_1–25_ for **[Ru­(NO)]** ^**0**^, S_9–25_ for **[Ru­(NS)]** ^**0**^ and **[Ru­(NSe)]** ^**0**^, and S_10–25_ for **[Ru­(NTe)]** ^**0**^, are located within the 2.0 to 3.0 eV energy range.

**Figure 6:**
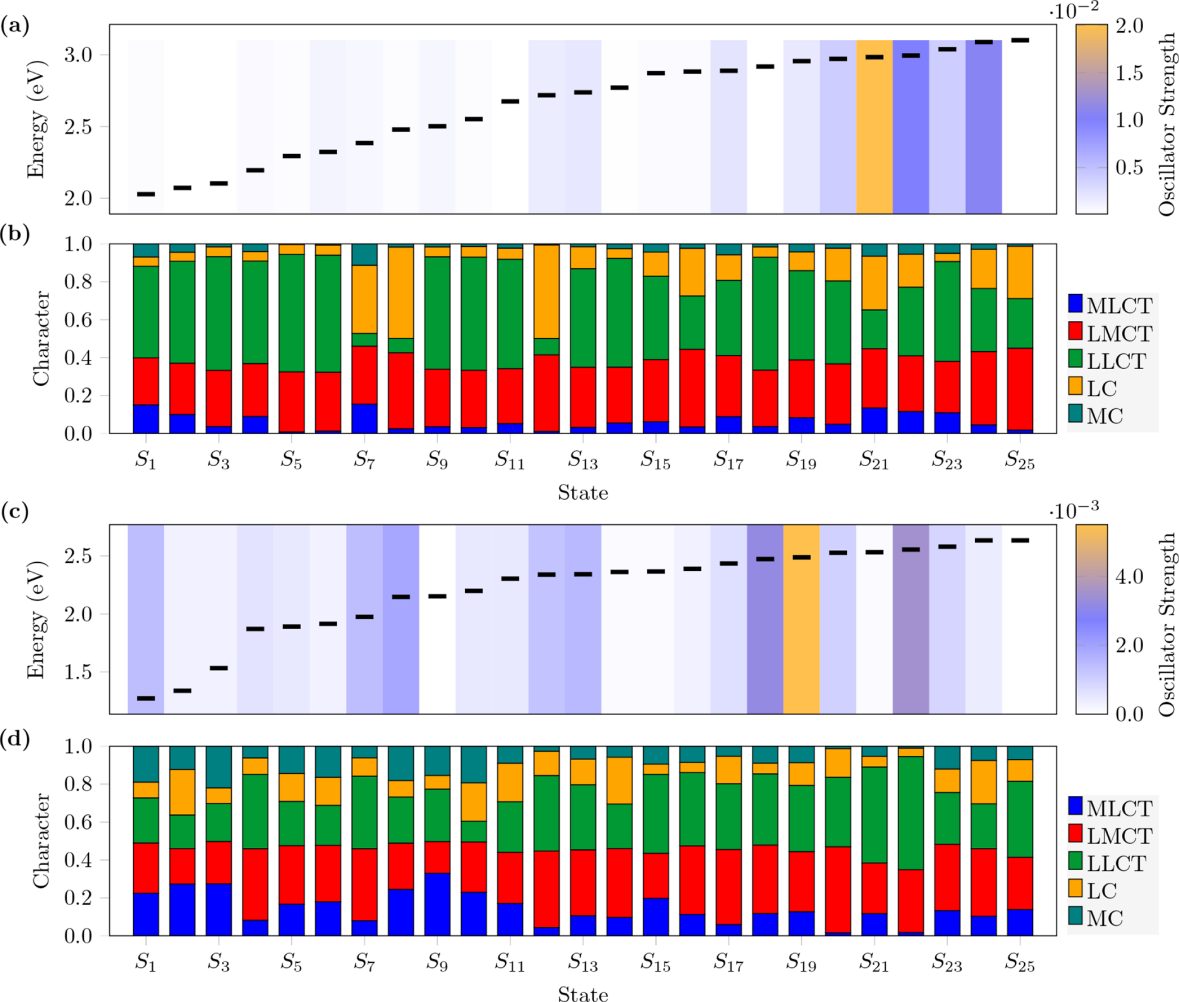
(a) and (c): Excitation energies (black lines) and oscillator strengths (color scale on the right); (b) and (d): Decomposition of the charge transfer number matrices for the first 25 excited states of **[Ru­(NO)]** ^**0**^ (a, b) and **[Ru­(NO)]** ^**–1**^ (c, d).

Theodore analysis shows that the main excitations are not simple HOMO–LUMO transitions but involve multiple orbital contributions. For **[Ru­(NO)]** ^**0**^, the most intense transition is S_21_ at 415 nm (*f* = 0.02), while for **[Ru­(NS)]** ^**0**^, **[Ru­(NSe)]** ^**0**^, and **[Ru­(NTe)]** ^**0**^, the strongest transitions occur at S_6_, with lower oscillator strengths (0.0039 – 0.0049) and bathochromically shifted to 688 – 726 nm. Importantly, the correlation between shorter excitation wavelengths and higher oscillator strength values explains why **[Ru­(NO)]** ^**0**^ displays its most intense transition at a higher state (S_21_). In contrast, in the heavier congeners, the strongest transitions are observed already at S_6_, and are related to the number of roots included in the calculation.

The decomposition of the charge transfer number matrices is shown in Figures 6b and S1b - S3b, and the corresponding charge transfer matrix plots are presented in Figures S7 - S10. These plots reveal that most transitions are predominantly attributed to ligand-to-ligand charge transfer (LLCT) (ca. 50%), followed by ligand-to-metal charge transfer (LMCT) (ca. 30%). The remaining contributions arise from metal-to-ligand charge transfer (MLCT), ligand-centered (LC), and metal-centered (MC) transitions. Some states, however, exhibit distinct behavior, namely S_7_, S_8_, S_12_, and S_21_ for **[Ru­(NO)]** ^**0**^; S_16_, S_17_, S_18_, S_21_, and S_25_ for **[Ru­(NS)]** ^**0**^; S_16_, S_17_, S_18_, and S_21_ for **[Ru­(NSe)]** ^**0**^; and S_18_, S_20_, and S_24_ for **[Ru­(NTe)]** ^**0**^. These states show a predominant ligand-centered character, in contrast to the other excited states, which are mainly dominated by LLCT contributions.

For the reduced species, the decomposition of the charge transfer number matrices (Figures 6d and S4b - S6b) shows that, compared to the oxidized species, there is an increase in MLCT, LC, and MC contributions, accompanied by a decrease in LLCT character. The charge transfer matrix plots (Figures S11 - S14) confirm that the evaluated transitions involve greater contributions from Cl, bidentate ligand (L), and Ru, whereas in the oxidized species, the dominant contributions originate primarily from the ligand (L) to NE and Ru.

The inclusion of solvent effects in the TD-DFT calculation was considered, and led to slight shifts in the transition energies and oscillator strengths, when compared to the gas-phase results, while preserving the overall profile and character of the electronic transitions (Figures S15 - S18). Notably, analysis with the TheoDORE software confirms that the main contributions to all transitions up to S_25_ remain dominated by charge-transfer processes (Figures S19 - S34). These results indicate that, although solvent polarization slightly modifies the spectral features, the fundamental electronic structure and the charge-transfer nature of the transitions do not change significantly.

## Conclusions

This study provides a comprehensive computational analysis of ruthenium-chalcogenonitrosyl (Ru–NE) compounds, shedding light on the effects of chalcogen substitution (E = O, S, Se, Te) on the bonding and electronic properties of these species before and after one-electron reduction. Our findings reveal critical trends in the geometry, bond strength, and electron distribution on these compounds, as well as the significant impact of one-electron reduction on the Ru–NE moiety. Spin density plots demonstrated that the reduction of the neutral species occurs primarily in the Ru–NE fragment, as also evidenced by the frontier orbitals. As a consequence of the one-electron reduction, the Ru–N–E angle, which is nearly linear in the neutral species (ranging from 169 to 177°), becomes bent in the reduced species, decreasing to approximately 142°. This change suggests a decrease in Pauli repulsion due to the reduction taking place in the π* orbitals of the **[Ru–NE]** ^**0**^ species.

The generalized Kohn–Sham energy decomposition analysis shows that, for the oxidized species, there is a strong interaction between Ru and NE, ranging from −248 to −226 kcal mol^–1^. The opposite behavior is observed for the reduced species, where Δ*E* ^*tot*^ increases to a range of −78 to −59 kcal mol^–1^. The dichotomous behavior is a consequence of a shift in the nature of the interaction, indicating that the NE group can be readily released after reduction of these neutral complexes due to the weakening of the Ru–NE bond. In turn, the wave function analysis shows that the LUMO of the neutral species is localized on the *d* orbitals of ruthenium and π* orbitals of NE, corroborating that the reduction will occur in this portion of the structure. Furthermore, the neutral species exhibit predominantly ligand-to-ligand charge transfer transitions, followed by ligand-to-metal charge transfer. In contrast, in the reduced species, the transitions acquire metal-to-ligand charge transfer, ligand-centered, and metal-centered character.

Finally, the present investigation demonstrates how the chalcogen substitution and one-electron reduction modulate the chemical bonding, electronic structure, and reactivity of Ru–NE compounds, enabling the rational tuning of these species for targeted applications, such as controlled ligand release or catalytic processes.

## Supplementary Material



## Notes

Population and bonding analysis; components for GKS-EDA; transition density matrix; simulated absorption spectra; Cartesian coordinates (PDF)
