# Supplementary material for: From NO to NTe: In Silico Study of Ruthenium Compounds Containing Chalcogenonitrosyl Ligands
Source: ACS Omega. 2025 Nov 25;10(48):59447–57. doi: 10.1021/acsomega.5c08990 (PMC12771179; doi:10.1021/acsomega.5c08990)
Supplement: Supplementary file 1 [file ao5c08990_si_001.pdf]

# Supporting Information: From NO to NTe: in silico study of ruthenium compounds containing chalcogenonitrosyl ligands

Vinícius Glitz, Richard Fragnani Cardoso, Giovanni Finoto Caramori, and Luis Henrique da Silveira Lacerda*

*Group of Molecular Electronic Structure and Materials, Departamento de Química, Universidade Federal de Santa Catarina, Campus Universitário Trindade, 88040-900, Florianópolis, SC, Brasil*

E-mail: luis.lacerda@ufsc.br

# List of Figures


S1 (a)Excitation energies and oscillator strength; (b) decomposition of the charge transfer number matrices of $[\mathbf{Ru(NS)}]^{\mathbf{0}}$ in the gas phase. . . . . . . . . . . . S-12

S2 (a)Excitation energies and oscillator strength; (b) decomposition of the charge transfer number matrices of $[\mathbf{Ru(NSe)}]^{\mathbf{0}}$ in the gas phase. . . . . . . . . . . S-12

S3 (a)Excitation energies and oscillator strength; (b) decomposition of the charge transfer number matrices of $[\mathbf{Ru(NTe)}]^{\mathbf{0}}$ in the gas phase. . . . . . . . . . . S-13

S4 (a)Excitation energies and oscillator strength; (b) decomposition of the charge transfer number matrices of $[\mathbf{Ru(NS)}]^{-\mathbf{1}}$ in the gas phase. . . . . . . . . . . S-13

S5 (a)Excitation energies and oscillator strength; (b) decomposition of the charge transfer number matrices of $[\mathbf{Ru(NSe)}]^{-\mathbf{1}}$ in the gas phase. . . . . . . . . . S-14

S6 (a)Excitation energies and oscillator strength; (b) decomposition of the charge transfer number matrices of $[\mathbf{Ru(NTe)}]^{-\mathbf{1}}$ in the gas phase. . . . . . . . . . S-14

S7 Electron-hole correlation plots of the Omega matrices for the individual states of $[\mathbf{Ru(NO)}]^{\mathbf{0}}$ in the gas phase. . . . . . . . . . . . . . . . . . . . . . . . . . . S-15

S8 Electron-hole correlation plots of the Omega matrices for the individual states of $[\mathbf{Ru(NS)}]^{\mathbf{0}}$ in the gas phase. . . . . . . . . . . . . . . . . . . . . . . . . . . S-16

S9 Electron-hole correlation plots of the Omega matrices for the individual states of $[\mathbf{Ru(NSe)}]^{\mathbf{0}}$ in the gas phase. . . . . . . . . . . . . . . . . . . . . . . . . S-17

S10 Electron-hole correlation plots of the Omega matrices for the individual states of $[\mathbf{Ru(NTe)}]^{\mathbf{0}}$ in the gas phase. . . . . . . . . . . . . . . . . . . . . . . . . S-18

S11 Electron-hole correlation plots of the Omega matrices for the individual states of $[\mathbf{Ru(NO)}]^{-\mathbf{1}}$ in the gas phase. . . . . . . . . . . . . . . . . . . . . . . . . S-19

S12 Electron-hole correlation plots of the Omega matrices for the individual states of $[\mathbf{Ru(NS)}]^{-\mathbf{1}}$ in the gas phase. . . . . . . . . . . . . . . . . . . . . . . . . S-20

S13 Electron-hole correlation plots of the Omega matrices for the individual states of $[\mathbf{Ru(NSe)}]^{-\mathbf{1}}$ in the gas phase. . . . . . . . . . . . . . . . . . . . . . . . S-21

S14 Electron-hole correlation plots of the Omega matrices for the individual states of $[\mathbf{Ru(NTe)}]^{-1}$ in the gas phase. . . . . . . . . . . . . . . . . . . . . . . . . S-22
S15 Calculated spectra for $[\mathbf{Ru(NO)}]^{\mathbf{0}}$ (top) and $[\mathbf{Ru(NO)}]^{-\mathbf{1}}$ (bottom) in the gas phase (black) and SMD(dichloromethane) (red). . . . . . . . . . . . . . . . S-23
S16 Calculated spectra for $[\mathbf{Ru(NS)}]^{\mathbf{0}}$ (top) and $[\mathbf{Ru(NS)}]^{-\mathbf{1}}$ (bottom) in the gas phase (black) and SMD(dichloromethane) (red). . . . . . . . . . . . . . . . S-24
S17 Calculated spectra for $[\mathbf{Ru(NSe)}]^{\mathbf{0}}$ (top) and $[\mathbf{Ru(NSe)}]^{-\mathbf{1}}$ (bottom) in the gas phase (black) and SMD(dichloromethane) (red). . . . . . . . . . . . . . . . S-25
S18 Calculated spectra for $[\mathbf{Ru(NTe)}]^{\mathbf{0}}$ (top) and $[\mathbf{Ru(NTe)}]^{-\mathbf{1}}$ (bottom) in the gas phase (black) and SMD(dichloromethane) (red). . . . . . . . . . . . . . . . S-26
S19 (a)Excitation energies and oscillator strength; (b) decomposition of the charge transfer number matrices of $[\mathbf{Ru(NO)}]^{\mathbf{0}}$ in the SMD(dichloromethane) environment. . . . . . . . . . . . . . . . . . . . . . . . . . . . . . . . . . . . . . . . . S-27
S20 (a)Excitation energies and oscillator strength; (b) decomposition of the charge transfer number matrices of $[\mathbf{Ru(NS)}]^{\mathbf{0}}$ in the SMD(dichloromethane) environment. . . . . . . . . . . . . . . . . . . . . . . . . . . . . . . . . . . . . . . . . S-27
S21 (a)Excitation energies and oscillator strength; (b) decomposition of the charge transfer number matrices of $[\mathbf{Ru(NSe)}]^{\mathbf{0}}$ in the SMD(dichloromethane) environment. . . . . . . . . . . . . . . . . . . . . . . . . . . . . . . . . . . . . . . . S-28
S22 (a)Excitation energies and oscillator strength; (b) decomposition of the charge transfer number matrices of $[\mathbf{Ru(NTe)}]^{\mathbf{0}}$ in the SMD(dichloromethane) environment. . . . . . . . . . . . . . . . . . . . . . . . . . . . . . . . . . . . . . . . S-28
S23 (a)Excitation energies and oscillator strength; (b) decomposition of the charge transfer number matrices of $[\mathbf{Ru(NO)}]^{-\mathbf{1}}$ in the SMD(dichloromethane) environment. . . . . . . . . . . . . . . . . . . . . . . . . . . . . . . . . . . . . . . . S-29

S24 (a)Excitation energies and oscillator strength; (b) decomposition of the charge transfer number matrices of **[Ru(NS)]$^{-1}$** in the SMD(dichloromethane) environment. . . . . . . . . . . . . . . . . . . . . . . . . . . . . . . . . . . . . S-29
S25 (a)Excitation energies and oscillator strength; (b) decomposition of the charge transfer number matrices of **[Ru(NSe)]$^{-1}$** in the SMD(dichloromethane) environment. . . . . . . . . . . . . . . . . . . . . . . . . . . . . . . . . . . . . S-30
S26 (a)Excitation energies and oscillator strength; (b) decomposition of the charge transfer number matrices of **[Ru(NTe)]$^{-1}$** in the SMD(dichloromethane) environment. . . . . . . . . . . . . . . . . . . . . . . . . . . . . . . . . . . . . S-30
S27 Electron-hole correlation plots of the Omega matrices for the individual states of **[Ru(NO)]$^{0}$** in the SMD(dichloromethane) environment. . . . . . . . . . . S-31
S28 Electron-hole correlation plots of the Omega matrices for the individual states of **[Ru(NS)]$^{0}$** in the SMD(dichloromethane) environment. . . . . . . . . . . S-32
S29 Electron-hole correlation plots of the Omega matrices for the individual states of **[Ru(NSe)]$^{0}$** in the SMD(dichloromethane) environment. . . . . . . . . . S-33
S30 Electron-hole correlation plots of the Omega matrices for the individual states of **[Ru(NTe)]$^{0}$** in the SMD(dichloromethane) environment. . . . . . . . . . S-34
S31 Electron-hole correlation plots of the Omega matrices for the individual states of **[Ru(NO)]$^{-1}$** in the SMD(dichloromethane) environment. . . . . . . . . . S-35
S32 Electron-hole correlation plots of the Omega matrices for the individual states of **[Ru(NS)]$^{-1}$** in the SMD(dichloromethane) environment. . . . . . . . . . S-36
S33 Electron-hole correlation plots of the Omega matrices for the individual states of **[Ru(NSe)]$^{-1}$** in the SMD(dichloromethane) environment. . . . . . . . . . S-37
S34 Electron-hole correlation plots of the Omega matrices for the individual states of **[Ru(NTe)]$^{-1}$** in the SMD(dichloromethane) environment. . . . . . . . . . S-38

# List of Tables


S1 Bond length, N−E stretching ($cm^{-1}$), Löwdin atomic charge ($q^{Löwdin}$) and bond order ($b_{AB}^{Löwdin}$), Hirshfeld and CHELPG charges for the free chalcogenonitrosyl obtained at the BP86/sapporo-TZP-2012 level. . . . . . . . . . . . . . . S-6

S2 Hirshfeld charge obtained at the BP86/sapporo-TZP-2012 level. . . . . . . . S-6

S3 Löwdin atomic charge and bond order obtained at the BP86/sapporo-TZP-2012 level. . . . . . . . . . . . . . . . . . . . . . . . . . . . . . . . . . . . . S-6

S4 CHELPG charge obtained at the BP86/sapporo-TZP-2012 level. . . . . . . . S-7

S5 Values of components for GKS-EDA (kcal $mol^{-1}$) for $[\mathbf{Ru(NO)}]^{\mathbf{0}}$, where the Ru−$\{NO\}^{+}$ bonds are decomposed by varying the Ru-N-O angle. . . . . . . S-7

S6 Values of components for GKS-EDA (kcal $mol^{-1}$) for $[\mathbf{Ru(NS)}]^{\mathbf{0}}$, where the Ru−$\{NS\}^{+}$ bonds are decomposed by varying the Ru-N-S angle. . . . . . . . S-8

S7 Values of components for GKS-EDA (kcal $mol^{-1}$) for $[\mathbf{Ru(NSe)}]^{\mathbf{0}}$, where the Ru−$\{NSe\}^{+}$ bonds are decomposed by varying the Ru-N-Se angle. . . . . . . S-8

S8 Values of components for GKS-EDA (kcal $mol^{-1}$) for $[\mathbf{Ru(NTe)}]^{\mathbf{0}}$, where the Ru−$\{NTe\}^{+}$ bonds are decomposed by varying the Ru-N-Te angle. . . . . . S-9

S9 Values of components for GKS-EDA (kcal $mol^{-1}$) for $[\mathbf{Ru(NO)}]^{\mathbf{-1}}$, where the Ru−$\{NO\}^{0}$ bonds are decomposed by varying the Ru-N-O angle. . . . . . S-9

S10 Values of components for GKS-EDA (kcal $mol^{-1}$) for $[\mathbf{Ru(NS)}]^{\mathbf{-1}}$, where the Ru−$\{NS\}^{0}$ bonds are decomposed by varying the Ru-N-S angle. . . . . . . . S-10

S11 Values of components for GKS-EDA (kcal $mol^{-1}$) for $[\mathbf{Ru(NSe)}]^{\mathbf{-1}}$, where the Ru−$\{NSe\}^{0}$ bonds are decomposed by varying the Ru-N-Se angle. . . . . S-10

S12 Values of components for GKS-EDA (kcal $mol^{-1}$) for $[\mathbf{Ru(NTe)}]^{\mathbf{-1}}$, where the Ru−$\{NTe\}^{0}$ bonds are decomposed by varying the Ru-N-Te angle. . . . S-11

S13 Energy in eV for the frontier orbitals of the oxidized and reduced species. . . S-11

Table S1: Bond length, N−E stretching ($cm^{-1}$), Löwdin atomic charge ($q^{Löwdin}$) and bond order ($b_{AB}^{Löwdin}$), Hirshfeld and CHELPG charges for the free chalcogenonitrosyl obtained at the BP86/sapporo-TZP-2012 level.

| | N−E | $\nu$(N−E)$_{sym}$ | Hirshfeld | | CHELPG | | $q^{Löwdin}$ | | $b_{AB}^{Löwdin}$ |
|---|---|---|---|---|---|---|---|---|---|
| | | | N | E | N | E | N | E | |
| **(NO)$^{0}$** | 1.069 | 2356 | 0.57 | 0.43 | 0.54 | 0.46 | 0.39 | 0.61 | 3.67 |
| **(NS)$^{0}$** | 1.444 | 1429 | 0.19 | 0.81 | 0.02 | 0.98 | 0.10 | 0.90 | 3.62 |
| **(NSe)$^{0}$** | 1.595 | 1135 | 0.13 | 0.87 | -0.02 | 1.02 | 0.06 | 0.94 | 3.55 |
| **(NTe)$^{0}$** | 1.807 | 954 | 0.04 | 0.96 | -0.12 | 1.12 | -0.05 | 1.05 | 3.51 |
| **(NO)$^{-1}$** | 1.158 | 1883 | 0.01 | -0.01 | -0.03 | 0.03 | -0.15 | 0.15 | 3.17 |
| **(NS)$^{-1}$** | 1.507 | 1206 | -0.14 | 0.14 | -0.24 | 0.24 | -0.22 | 0.22 | 3.16 |
| **(NSe)$^{-1}$** | 1.665 | 963 | -0.16 | 0.16 | -0.25 | 0.25 | -0.23 | 0.23 | 2.98 |
| **(NTe)$^{-1}$** | 1.871 | 818 | -0.20 | 0.20 | -0.29 | 0.29 | -0.28 | 0.28 | 2.91 |

Table S2: Hirshfeld charge obtained at the BP86/sapporo-TZP-2012 level.

| | Ru | N | E | Cl | L | (NE)$^{+1/0}$ |
|---|---|---|---|---|---|---|
| **[Ru(NO)]$^{0}$** | 0.29 | 0.02 | -0.02 | -0.26 | -0.03 | 0.00 |
| **[Ru(NS)]$^{0}$** | 0.32 | -0.12 | 0.09 | -0.27 | -0.02 | -0.03 |
| **[Ru(NSe)]$^{0}$** | 0.32 | -0.15 | 0.10 | -0.27 | -0.01 | -0.04 |
| **[Ru(NTe)]$^{0}$** | 0.33 | -0.18 | 0.12 | -0.27 | 0.00 | -0.06 |
| **[Ru(NO)]$^{-1}$** | 0.21 | -0.06 | -0.13 | -0.46 | -0.56 | -0.20 |
| **[Ru(NS)]$^{-1}$** | 0.23 | -0.19 | -0.11 | -0.39 | -0.54 | -0.30 |
| **[Ru(NSe)]$^{-1}$** | 0.25 | -0.20 | -0.14 | -0.40 | -0.51 | -0.34 |
| **[Ru(NTe)]$^{-1}$** | 0.26 | -0.23 | -0.16 | -0.40 | -0.47 | -0.39 |

Table S3: Löwdin atomic charge and bond order obtained at the BP86/sapporo-TZP-2012 level.

| | Atomic charge | | | | | | Bond order | | | |
|---|---|---|---|---|---|---|---|---|---|---|
| | Ru | N | E | Cl | L | (NE)$^{+1/0}$ | Ru−N | N−E | Ru−Cl | Ru−S$^{a}$ |
| **[Ru(NO)]$^{0}$** | -1.21 | 0.05 | 0.19 | 0.06 | 0.90 | 0.24 | 2.17 | 2.73 | 1.30 | 1.08 |
| **[Ru(NS)]$^{0}$** | -1.19 | -0.01 | 0.23 | 0.05 | 0.91 | 0.22 | 2.06 | 2.47 | 1.26 | 1.08 |
| **[Ru(NSe)]$^{0}$** | -1.19 | 0.02 | 0.19 | 0.05 | 0.93 | 0.21 | 2.15 | 2.23 | 1.24 | 1.08 |
| **[Ru(NTe)]$^{0}$** | -1.19 | -0.02 | 0.22 | 0.04 | 0.95 | 0.20 | 2.19 | 2.03 | 1.22 | 1.08 |
| **[Ru(NO)]$^{-1}$** | -1.08 | -0.03 | 0.07 | -0.30 | 0.34 | 0.04 | 1.95 | 2.58 | 0.67 | 1.02 |
| **[Ru(NS)]$^{-1}$** | -1.24 | -0.06 | 0.01 | -0.13 | 0.43 | -0.05 | 1.79 | 2.29 | 1.05 | 1.07 |
| **[Ru(NSe)]$^{-1}$** | -1.24 | -0.04 | -0.05 | -0.14 | 0.46 | -0.09 | 1.91 | 1.99 | 1.04 | 1.06 |
| **[Ru(NTe)]$^{-1}$** | -1.23 | -0.07 | -0.06 | -0.14 | 0.51 | -0.14 | 1.97 | 1.79 | 1.03 | 1.06 |

[a] Average Ru–S bond order.

Table S4: CHELPG charge obtained at the BP86/sapporo-TZP-2012 level.

| | Ru | N | E | Cl | L | $(NE)^{+1/0}$ |
|---|---|---|---|---|---|---|
| **[Ru(NO)]$^{\mathbf{0}}$** | 0.34 | 0.18 | -0.11 | -0.28 | -0.12 | 0.07 |
| **[Ru(NS)]$^{\mathbf{0}}$** | 0.55 | -0.14 | 0.09 | -0.34 | -0.17 | -0.05 |
| **[Ru(NSe)]$^{\mathbf{0}}$** | 0.56 | -0.17 | 0.11 | -0.34 | -0.16 | -0.06 |
| **[Ru(NTe)]$^{\mathbf{0}}$** | 0.66 | -0.19 | 0.13 | -0.36 | -0.24 | -0.06 |
| **[Ru(NO)]$^{\mathbf{-1}}$** | 0.53 | -0.16 | -0.14 | -0.57 | -0.66 | -0.30 |
| **[Ru(NS)]$^{\mathbf{-1}}$** | 0.76 | -0.34 | -0.14 | -0.52 | -0.76 | -0.48 |
| **[Ru(NSe)]$^{\mathbf{-1}}$** | 0.82 | -0.36 | -0.17 | -0.53 | -0.77 | -0.52 |
| **[Ru(NTe)]$^{\mathbf{-1}}$** | 0.83 | -0.32 | -0.20 | -0.53 | -0.78 | -0.52 |

Table S5: Values of components for GKS-EDA (kcal mol$^{-1}$) for **[Ru(NO)]$^{\mathbf{0}}$**, where the Ru$-\{NO\}^+$ bonds are decomposed by varying the Ru-N-O angle.

| angle | $\Delta E^{ele}$ | $\Delta E^{exrep}$ | $\Delta E^{pol}$ | $\Delta E^{corr}$ | $\Delta E^{disp}$ | $\Delta E^{tot}$ |
|---|---|---|---|---|---|---|
| 120 | -102.35 | 139.66 | -169.77 | -91.28 | -4.36 | -228.10 |
| 125 | -102.77 | 142.29 | -176.37 | -90.51 | -4.35 | -231.71 |
| 129 | -103.82 | 145.09 | -182.35 | -89.45 | -4.31 | -234.84 |
| 133 | -105.01 | 148.06 | -188.22 | -88.21 | -4.27 | -237.65 |
| 137 | -106.20 | 151.19 | -193.95 | -86.95 | -4.23 | -240.14 |
| 141 | -107.28 | 154.21 | -199.24 | -85.82 | -4.19 | -242.32 |
| 146 | -108.17 | 156.93 | -203.90 | -84.87 | -4.16 | -244.16 |
| 150 | -108.83 | 159.24 | -207.82 | -84.11 | -4.13 | -245.66 |
| 154 | -109.22 | 160.98 | -210.91 | -83.54 | -4.11 | -246.80 |
| 159 | -109.33 | 162.14 | -213.24 | -83.12 | -4.08 | -247.64 |
| 163 | -109.11 | 162.54 | -214.71 | -82.84 | -4.06 | -248.18 |
| 167 | -108.58 | 162.28 | -215.47 | -82.67 | -4.04 | -248.48 |
| 171 | -107.75 | 161.51 | -215.68 | -82.64 | -4.04 | -248.60 |
| 176 | -106.73 | 160.58 | -215.61 | -82.67 | -4.05 | -248.49 |
| 180 | -104.43 | 160.73 | -216.66 | -83.57 | -4.44 | -248.39 |

Table S6: Values of components for GKS-EDA (kcal mol$^{-1}$) for $\mathbf{[Ru(NS)]^0}$, where the Ru$-\{NS\}^+$ bonds are decomposed by varying the Ru-N-S angle.

| angle | $\Delta E^{ele}$ | $\Delta E^{exrep}$ | $\Delta E^{pol}$ | $\Delta E^{corr}$ | $\Delta E^{disp}$ | $\Delta E^{tot}$ |
|---|---|---|---|---|---|---|
| 120 | -126.16 | 188.23 | -183.26 | -78.70 | -10.36 | -210.24 |
| 124 | -125.88 | 182.63 | -181.88 | -79.62 | -10.11 | -214.86 |
| 129 | -126.32 | 180.46 | -185.16 | -78.59 | -9.64 | -219.25 |
| 137 | -128.07 | 180.30 | -192.38 | -76.66 | -9.14 | -225.96 |
| 141 | -129.09 | 181.62 | -196.74 | -75.49 | -8.91 | -228.62 |
| 146 | -130.06 | 183.24 | -201.04 | -74.37 | -8.70 | -230.92 |
| 150 | -130.84 | 184.81 | -204.91 | -73.40 | -8.50 | -232.83 |
| 154 | -131.39 | 186.33 | -208.33 | -72.64 | -8.32 | -234.35 |
| 159 | -131.65 | 187.63 | -211.19 | -72.10 | -8.18 | -235.49 |
| 163 | -131.55 | 188.37 | -213.16 | -71.81 | -8.07 | -236.22 |
| 167 | -131.19 | 188.81 | -214.75 | -71.61 | -7.98 | -236.72 |
| 171 | -130.60 | 188.55 | -215.30 | -71.59 | -7.93 | -236.87 |
| 176 | -129.73 | 187.91 | -215.43 | -71.66 | -7.92 | -236.84 |
| 180 | -128.64 | 186.94 | -214.78 | -71.99 | -8.03 | -236.51 |

Table S7: Values of components for GKS-EDA (kcal mol$^{-1}$) for $\mathbf{[Ru(NSe)]^0}$, where the Ru$-\{NSe\}^+$ bonds are decomposed by varying the Ru-N-Se angle.

| angle | $\Delta E^{ele}$ | $\Delta E^{exrep}$ | $\Delta E^{pol}$ | $\Delta E^{corr}$ | $\Delta E^{disp}$ | $\Delta E^{tot}$ |
|---|---|---|---|---|---|---|
| 120 | -125.33 | 193.84 | -196.92 | -73.66 | -11.68 | -213.74 |
| 124 | -125.64 | 189.60 | -195.10 | -74.81 | -11.38 | -217.31 |
| 129 | -126.32 | 188.51 | -198.41 | -73.83 | -10.84 | -220.88 |
| 133 | -127.26 | 188.46 | -201.41 | -73.06 | -10.52 | -223.79 |
| 137 | -128.35 | 189.44 | -205.20 | -71.99 | -10.22 | -226.32 |
| 141 | -129.45 | 191.24 | -209.48 | -70.87 | -10.01 | -228.56 |
| 146 | -130.48 | 192.98 | -213.44 | -69.71 | -9.71 | -230.36 |
| 150 | -131.32 | 194.93 | -217.28 | -68.75 | -9.48 | -231.91 |
| 154 | -131.90 | 196.75 | -220.69 | -68.00 | -9.30 | -233.14 |
| 159 | -132.15 | 198.25 | -223.62 | -67.44 | -9.14 | -234.10 |
| 163 | -132.08 | 199.29 | -225.83 | -67.11 | -9.01 | -234.74 |
| 167 | -131.71 | 199.73 | -227.28 | -66.94 | -8.91 | -235.11 |
| 171 | -131.17 | 199.62 | -227.88 | -66.93 | -8.84 | -235.20 |
| 176 | -130.41 | 199.05 | -227.96 | -67.00 | -8.82 | -235.14 |
| 180 | -129.36 | 197.96 | -227.33 | -67.20 | -8.90 | -234.83 |

Table S8: Values of components for GKS-EDA (kcal $mol^{-1}$) for $\mathbf{[Ru(NTe)]^0}$, where the Ru$-\{NTe\}^+$ bonds are decomposed by varying the Ru-N-Te angle.

| angle | $\Delta E^{ele}$ | $\Delta E^{exrep}$ | $\Delta E^{pol}$ | $\Delta E^{corr}$ | $\Delta E^{disp}$ | $\Delta E^{tot}$ |
|---|---|---|---|---|---|---|
| 120 | -131.38 | 196.81 | -187.70 | -70.93 | -14.22 | -207.41 |
| 124 | -131.95 | 197.41 | -191.59 | -70.73 | -13.85 | -210.71 |
| 129 | -132.68 | 198.91 | -196.71 | -69.74 | -13.50 | -213.72 |
| 133 | -133.05 | 200.47 | -204.65 | -67.16 | -12.53 | -216.93 |
| 137 | -133.88 | 201.78 | -208.97 | -66.08 | -12.10 | -219.25 |
| 141 | -134.79 | 203.56 | -213.21 | -65.02 | -11.74 | -221.19 |
| 146 | -135.61 | 205.60 | -217.37 | -63.99 | -11.43 | -222.79 |
| 150 | -136.26 | 207.71 | -221.32 | -63.09 | -11.16 | -224.12 |
| 154 | -136.62 | 209.56 | -224.86 | -62.36 | -10.92 | -225.19 |
| 159 | -136.68 | 211.07 | -227.77 | -61.86 | -10.73 | -225.97 |
| 163 | -136.43 | 212.13 | -230.13 | -61.53 | -10.58 | -226.53 |
| 167 | -135.90 | 212.61 | -231.74 | -61.36 | -10.44 | -226.84 |
| 171 | -135.26 | 212.55 | -232.51 | -61.33 | -10.36 | -226.91 |
| 176 | -134.51 | 212.07 | -232.68 | -61.38 | -10.31 | -226.81 |
| 180 | -133.61 | 210.80 | -231.85 | -61.46 | -10.32 | -226.45 |

Table S9: Values of components for GKS-EDA (kcal $mol^{-1}$) for $\mathbf{[Ru(NO)]^{-1}}$, where the Ru$-\{NO\}^0$ bonds are decomposed by varying the Ru-N-O angle.

| angle | $\Delta E^{ele}$ | $\Delta E^{exrep}$ | $\Delta E^{pol}$ | $\Delta E^{corr}$ | $\Delta E^{disp}$ | $\Delta E^{tot}$ |
|---|---|---|---|---|---|---|
| 120 | -54.77 | 139.71 | -55.70 | -65.29 | -4.69 | -40.75 |
| 124 | -37.67 | 132.05 | -67.74 | -65.44 | -4.67 | -43.47 |
| 128 | -59.54 | 146.65 | -62.85 | -65.16 | -4.66 | -45.56 |
| 132 | -57.24 | 146.64 | -67.21 | -64.71 | -4.62 | -47.14 |
| 137 | -61.25 | 150.19 | -68.42 | -64.17 | -4.58 | -48.24 |
| 141 | -52.95 | 147.88 | -74.73 | -71.12 | -8.36 | -59.30 |
| 145 | -60.54 | 153.36 | -73.16 | -70.44 | -8.32 | -59.10 |
| 149 | -55.07 | 158.30 | -80.63 | -69.54 | -8.34 | -55.29 |
| 153 | -54.17 | 161.19 | -82.88 | -70.06 | -8.37 | -54.28 |
| 157 | -59.54 | 170.08 | -82.98 | -72.22 | -8.42 | -53.08 |
| 161 | -65.60 | 176.81 | -76.76 | -77.65 | -8.49 | -51.69 |
| 166 | -68.86 | 188.84 | -79.19 | -83.02 | -8.56 | -50.78 |
| 170 | -70.74 | 194.91 | -83.49 | -83.75 | -8.51 | -51.58 |
| 174 | -73.90 | 200.91 | -86.92 | -83.81 | -8.51 | -52.23 |
| 178 | -71.67 | 196.23 | -84.33 | -83.59 | -8.38 | -51.75 |

Table S10: Values of components for GKS-EDA (kcal mol$^{-1}$) for $\mathbf{[Ru(NS)]^{-1}}$, where the Ru$-$\{NS\}$^0$ bonds are decomposed by varying the Ru-N-S angle.

| angle | $\Delta E^{ele}$ | $\Delta E^{exrep}$ | $\Delta E^{pol}$ | $\Delta E^{corr}$ | $\Delta E^{disp}$ | $\Delta E^{tot}$ |
|---|---|---|---|---|---|---|
| 120 | -69.46 | 170.81 | -83.20 | -62.85 | -9.65 | -54.35 |
| 124 | -72.80 | 173.54 | -86.97 | -62.09 | -9.57 | -57.90 |
| 129 | -68.36 | 172.79 | -94.55 | -61.08 | -9.48 | -60.69 |
| 133 | -72.31 | 175.33 | -96.45 | -60.00 | -9.38 | -62.81 |
| 137 | -66.00 | 171.00 | -101.25 | -58.90 | -9.27 | -64.41 |
| 141 | -74.53 | 176.77 | -100.70 | -57.76 | -9.17 | -65.41 |
| 146 | -69.44 | 173.54 | -104.35 | -56.63 | -9.06 | -65.94 |
| 150 | -73.21 | 175.10 | -103.58 | -55.48 | -8.94 | -66.11 |
| 154 | -73.07 | 174.20 | -103.98 | -54.33 | -8.83 | -66.01 |
| 159 | -72.51 | 172.43 | -103.70 | -53.26 | -8.68 | -65.72 |
| 163 | -71.55 | 168.87 | -101.93 | -52.28 | -8.41 | -65.29 |
| 167 | -71.94 | 167.00 | -100.17 | -51.24 | -8.33 | -64.68 |
| 171 | -72.08 | 164.61 | -98.00 | -50.32 | -8.28 | -64.07 |
| 176 | -71.95 | 162.68 | -96.34 | -49.71 | -8.25 | -63.57 |
| 180 | -72.29 | 161.71 | -94.96 | -49.55 | -8.23 | -63.31 |

Table S11: Values of components for GKS-EDA (kcal mol$^{-1}$) for $\mathbf{[Ru(NSe)]^{-1}}$, where the Ru$-$\{NSe\}$^0$ bonds are decomposed by varying the Ru-N-Se angle.

| angle | $\Delta E^{ele}$ | $\Delta E^{exrep}$ | $\Delta E^{pol}$ | $\Delta E^{corr}$ | $\Delta E^{disp}$ | $\Delta E^{tot}$ |
|---|---|---|---|---|---|---|
| 120 | -70.67 | 185.55 | -103.87 | -62.76 | -10.92 | -62.67 |
| 124 | -66.88 | 184.88 | -111.32 | -61.65 | -10.81 | -65.78 |
| 129 | -71.39 | 187.43 | -112.96 | -60.46 | -10.68 | -68.05 |
| 133 | -64.93 | 184.50 | -119.53 | -59.26 | -10.53 | -69.74 |
| 137 | -73.36 | 188.75 | -117.80 | -58.09 | -10.38 | -70.88 |
| 141 | -73.98 | 188.79 | -119.21 | -56.94 | -10.23 | -71.57 |
| 146 | -73.63 | 188.05 | -120.55 | -55.61 | -10.18 | -71.93 |
| 150 | -72.74 | 186.70 | -121.28 | -54.47 | -10.03 | -71.82 |
| 154 | -73.24 | 185.71 | -120.84 | -53.32 | -9.86 | -71.55 |
| 159 | -74.18 | 183.91 | -119.01 | -52.24 | -9.65 | -71.16 |
| 163 | -71.48 | 178.13 | -116.90 | -51.16 | -9.12 | -70.53 |
| 167 | -71.75 | 175.63 | -114.62 | -50.05 | -9.06 | -69.85 |
| 171 | -72.11 | 173.28 | -112.17 | -49.14 | -9.05 | -69.20 |
| 176 | -71.79 | 171.02 | -110.30 | -48.54 | -9.04 | -68.66 |
| 180 | -71.68 | 169.85 | -109.15 | -48.37 | -9.02 | -68.37 |

Table S12: Values of components for GKS-EDA (kcal mol$^{-1}$) for $[\mathbf{Ru(NTe)}]^{-\mathbf{1}}$, where the Ru$-\{$NTe$\}^0$ bonds are decomposed by varying the Ru-N-Te angle.

| angle | $\Delta E^{ele}$ | $\Delta E^{exrep}$ | $\Delta E^{pol}$ | $\Delta E^{corr}$ | $\Delta E^{disp}$ | $\Delta E^{tot}$ |
|---|---|---|---|---|---|---|
| 120 | -77.51 | 201.27 | -122.36 | -59.49 | -13.21 | -71.31 |
| 124 | -69.18 | 197.97 | -129.96 | -59.62 | -13.02 | -73.81 |
| 129 | -76.69 | 201.04 | -128.58 | -58.67 | -12.83 | -75.73 |
| 133 | -78.15 | 201.69 | -130.56 | -57.44 | -12.63 | -77.09 |
| 137 | -77.90 | 201.29 | -132.76 | -56.18 | -12.41 | -77.96 |
| 141 | -79.37 | 201.39 | -133.27 | -54.96 | -12.17 | -78.39 |
| 146 | -78.71 | 200.20 | -134.24 | -53.76 | -11.92 | -78.44 |
| 150 | -79.47 | 198.96 | -133.37 | -52.52 | -11.73 | -78.14 |
| 154 | -80.16 | 197.11 | -131.84 | -50.97 | -11.76 | -77.62 |
| 159 | -80.21 | 193.41 | -129.57 | -49.89 | -10.95 | -77.21 |
| 163 | -78.62 | 188.64 | -126.88 | -48.09 | -10.49 | -75.44 |
| 167 | -76.77 | 185.52 | -126.20 | -46.93 | -10.46 | -74.85 |
| 171 | -77.59 | 183.41 | -123.61 | -46.02 | -10.45 | -74.26 |
| 176 | -77.27 | 181.66 | -122.23 | -45.52 | -10.46 | -73.81 |
| 180 | -76.30 | 180.64 | -122.03 | -45.45 | -10.48 | -73.61 |

Table S13: Energy in eV for the frontier orbitals of the oxidized and reduced species.

| oxidized | HOMO | LUMO | | |
|---|---|---|---|---|
| $[\mathbf{Ru(NO)}]^{\mathbf{0}}$ | -5.06 | -3.10 | | |
| $[\mathbf{Ru(NS)}]^{\mathbf{0}}$ | -5.04 | -3.60 | | |
| $[\mathbf{Ru(NSe)}]^{\mathbf{0}}$ | -5.06 | -3.62 | | |
| $[\mathbf{Ru(NTe)}]^{\mathbf{0}}$ | -5.06 | -3.71 | | |
| reduced | SOMO($\alpha$) | SOMO($\beta$) | LUMO($\alpha$) | LUMO($\beta$) |
| $[\mathbf{Ru(NO)}]^{-\mathbf{1}}$ | -0.70 | -1.44 | 0.54 | 0.10 |
| $[\mathbf{Ru(NS)}]^{-\mathbf{1}}$ | -0.43 | -1.37 | 0.26 | 0.32 |
| $[\mathbf{Ru(NSe)}]^{-\mathbf{1}}$ | -0.57 | -1.47 | 0.16 | 0.13 |
| $[\mathbf{Ru(NTe)}]^{-\mathbf{1}}$ | -0.68 | -1.50 | 0.00 | -0.03 |

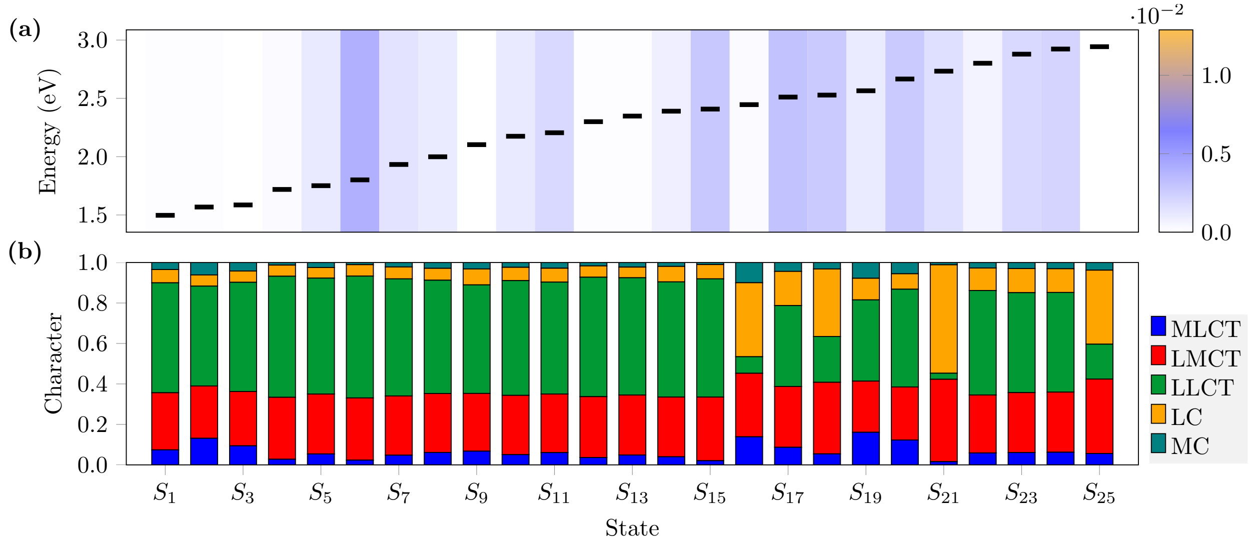


Figure S1: (a)Excitation energies and oscillator strength; (b) decomposition of the charge transfer number matrices of **[Ru(NS)]⁰** in the gas phase.

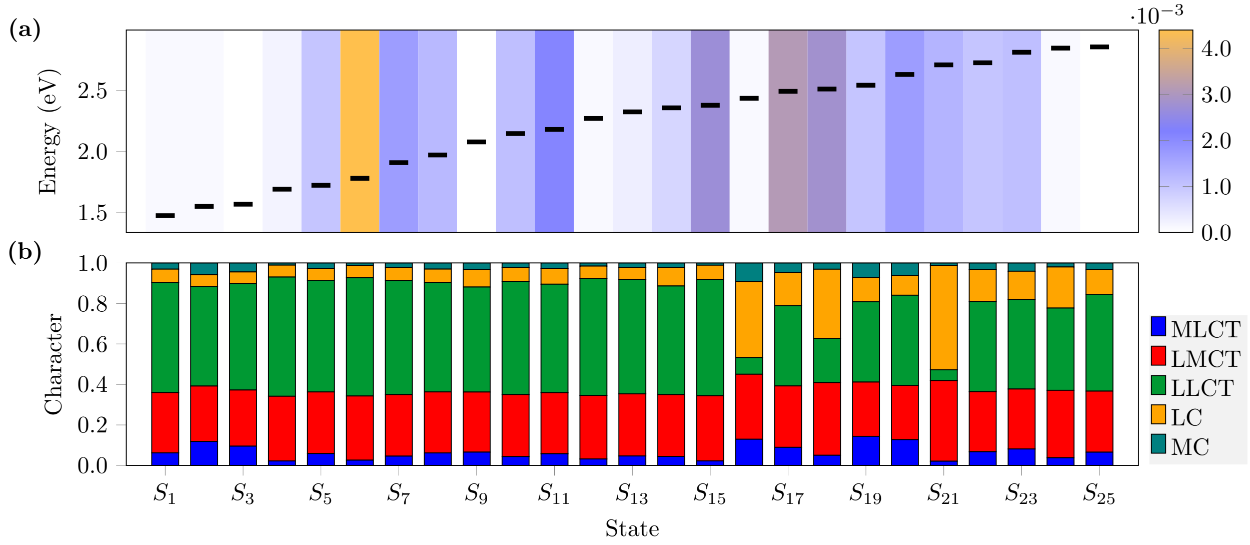


Figure S2: (a)Excitation energies and oscillator strength; (b) decomposition of the charge transfer number matrices of **[Ru(NSe)]⁰** in the gas phase.

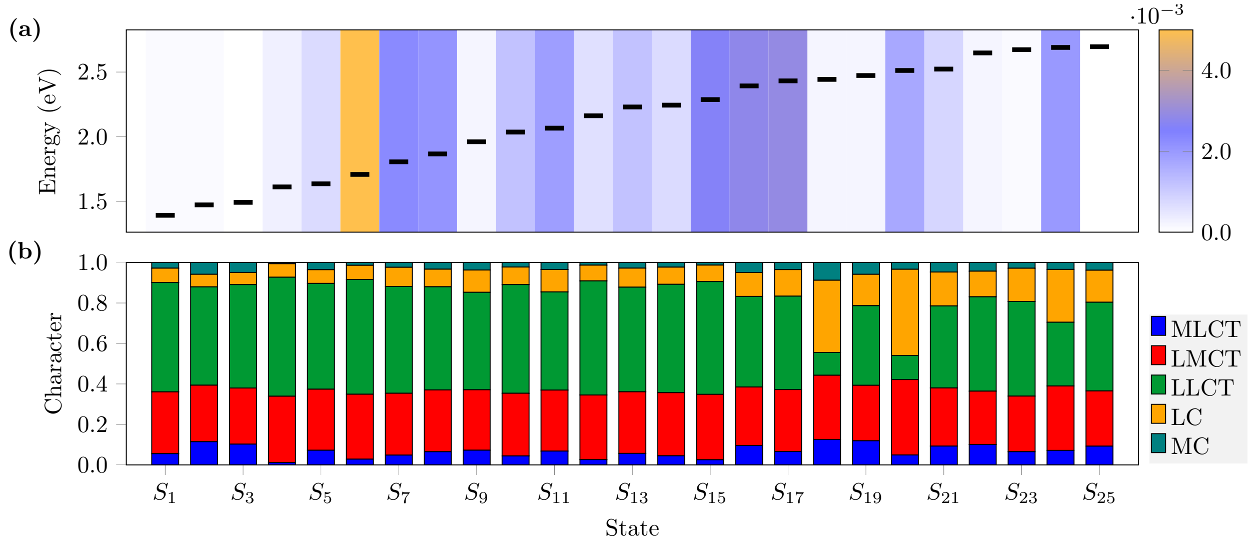


Figure S3: (a)Excitation energies and oscillator strength; (b) decomposition of the charge transfer number matrices of $[\mathbf{Ru(NTe)}]^{\mathbf{0}}$ in the gas phase.

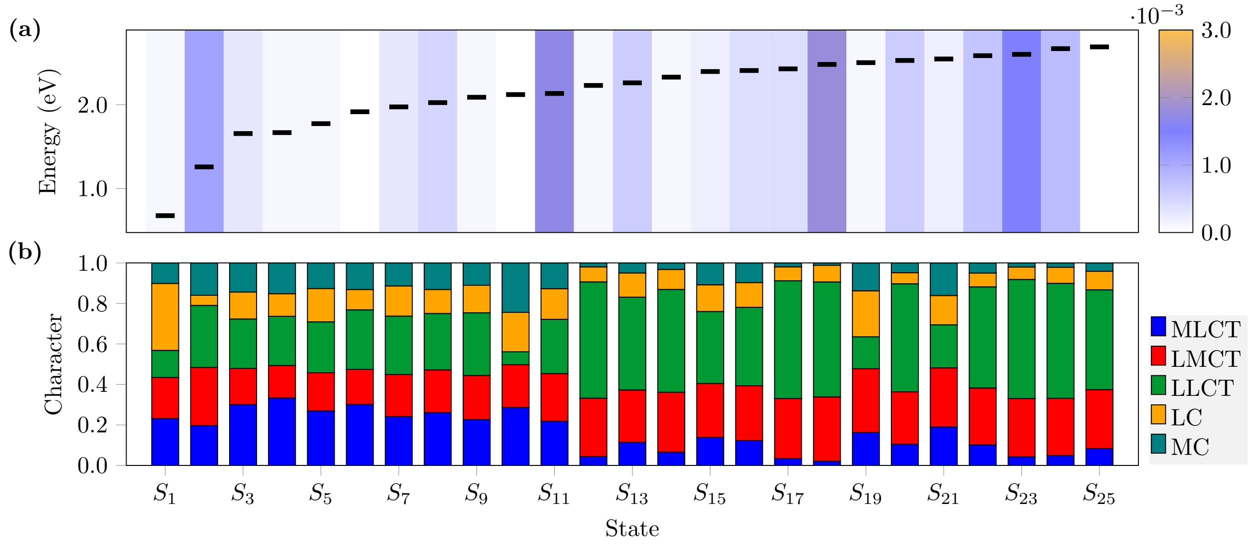


Figure S4: (a)Excitation energies and oscillator strength; (b) decomposition of the charge transfer number matrices of $[\mathbf{Ru(NS)}]^{-\mathbf{1}}$ in the gas phase.

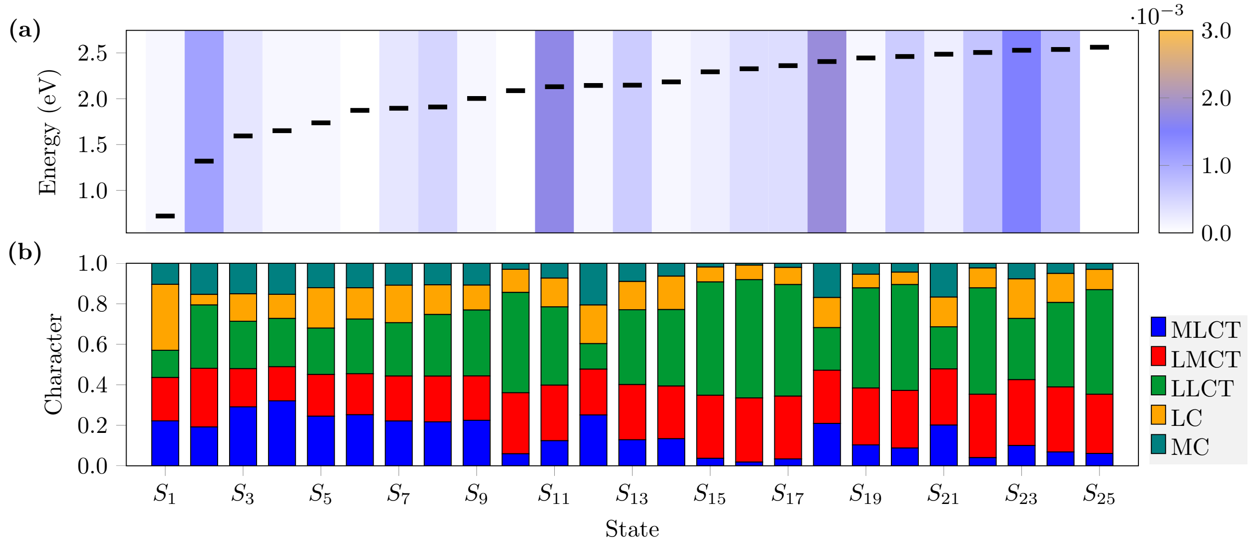


Figure S5: (a)Excitation energies and oscillator strength; (b) decomposition of the charge transfer number matrices of $[\mathbf{Ru(NSe)}]^{-1}$ in the gas phase.

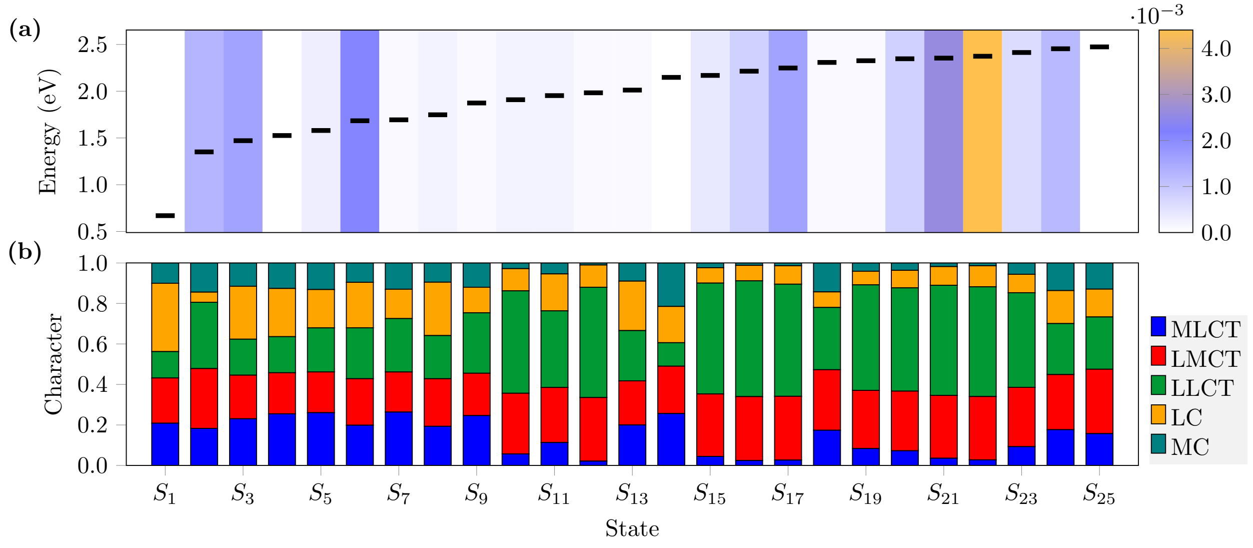


Figure S6: (a)Excitation energies and oscillator strength; (b) decomposition of the charge transfer number matrices of $[\mathbf{Ru(NTe)}]^{-1}$ in the gas phase.

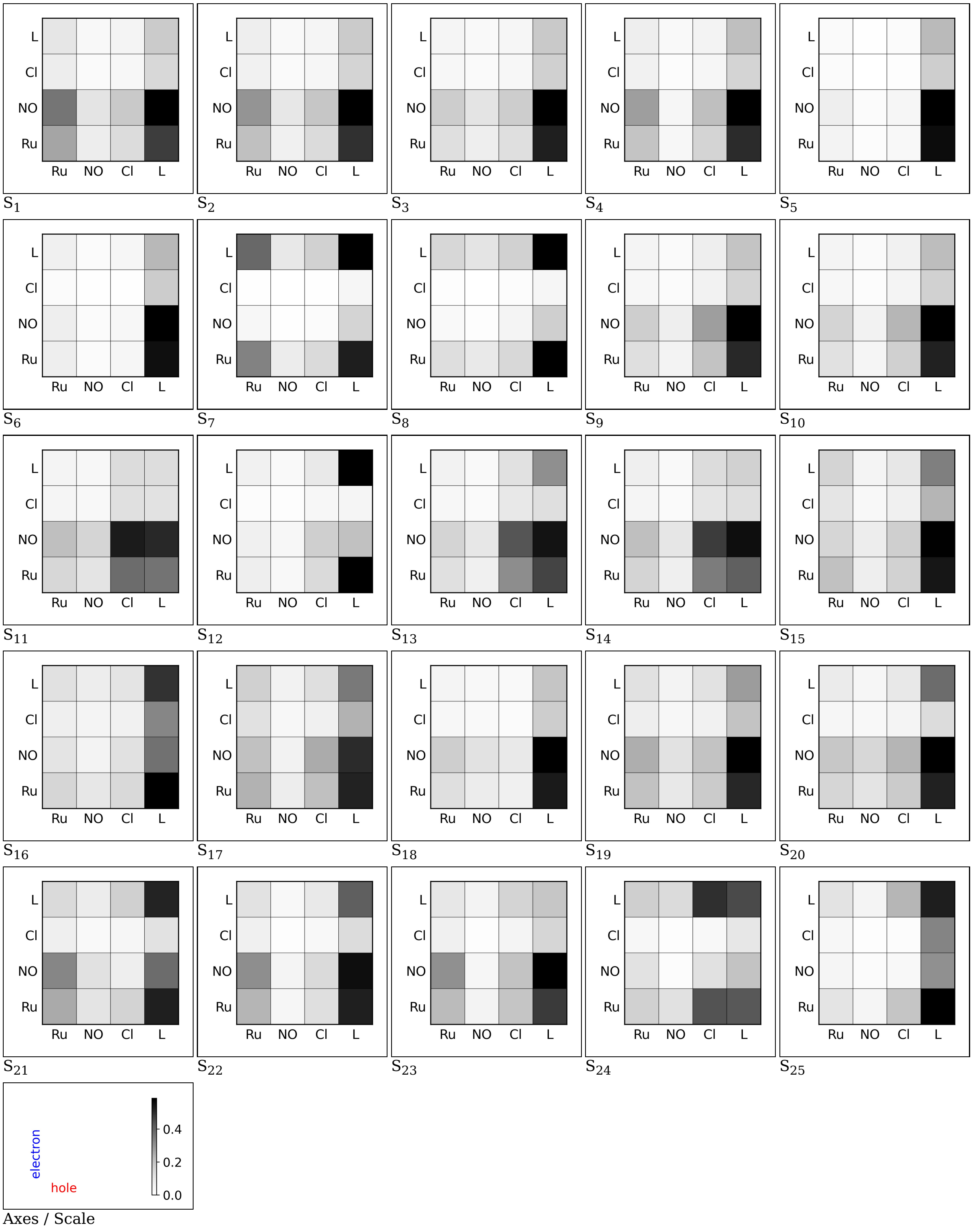


Figure S7: Electron-hole correlation plots of the Omega matrices for the individual states of $\mathbf{[Ru(NO)]^0}$ in the gas phase.

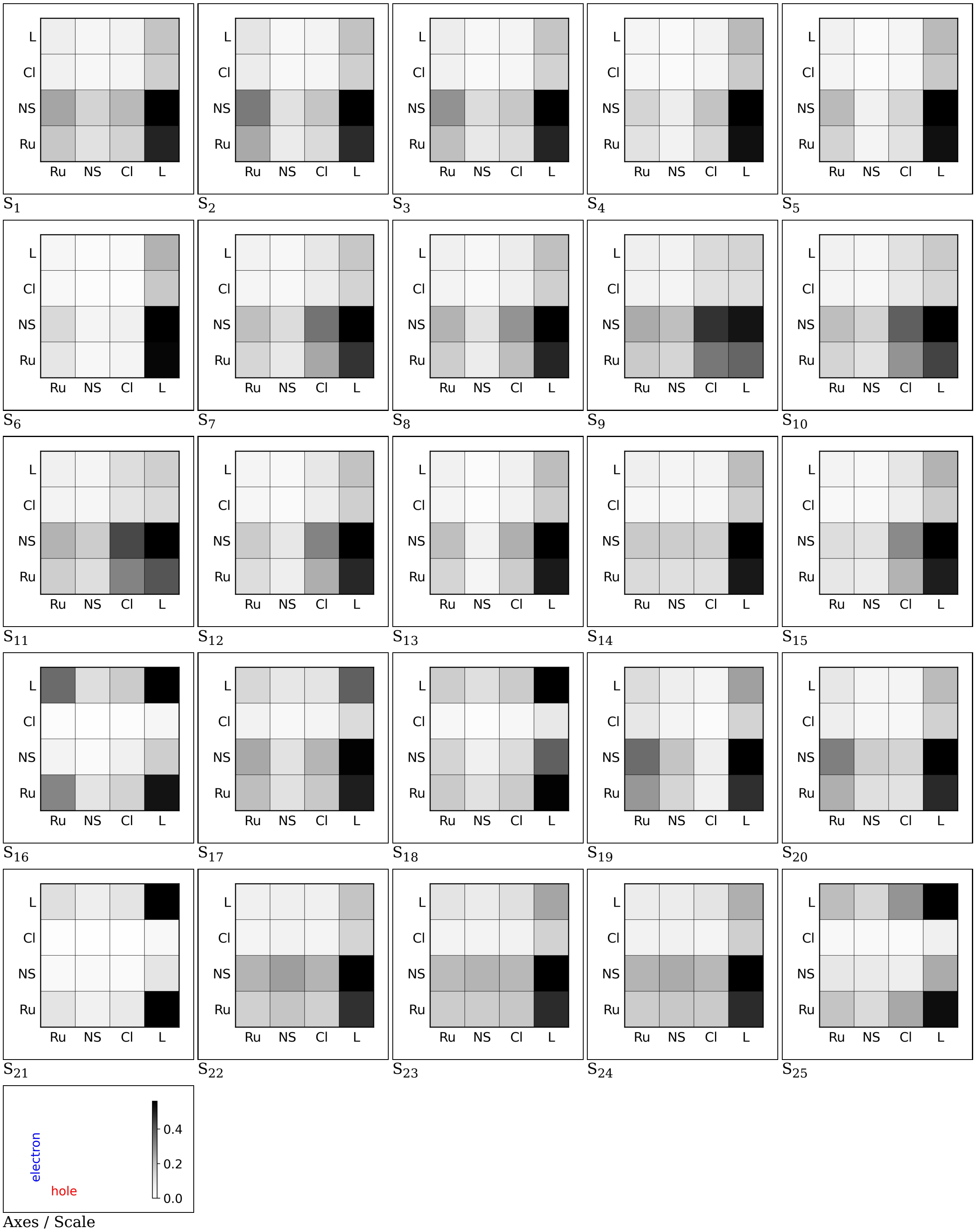


Figure S8: Electron-hole correlation plots of the Omega matrices for the individual states of **[Ru(NS)]$^{\mathbf{0}}$** in the gas phase.

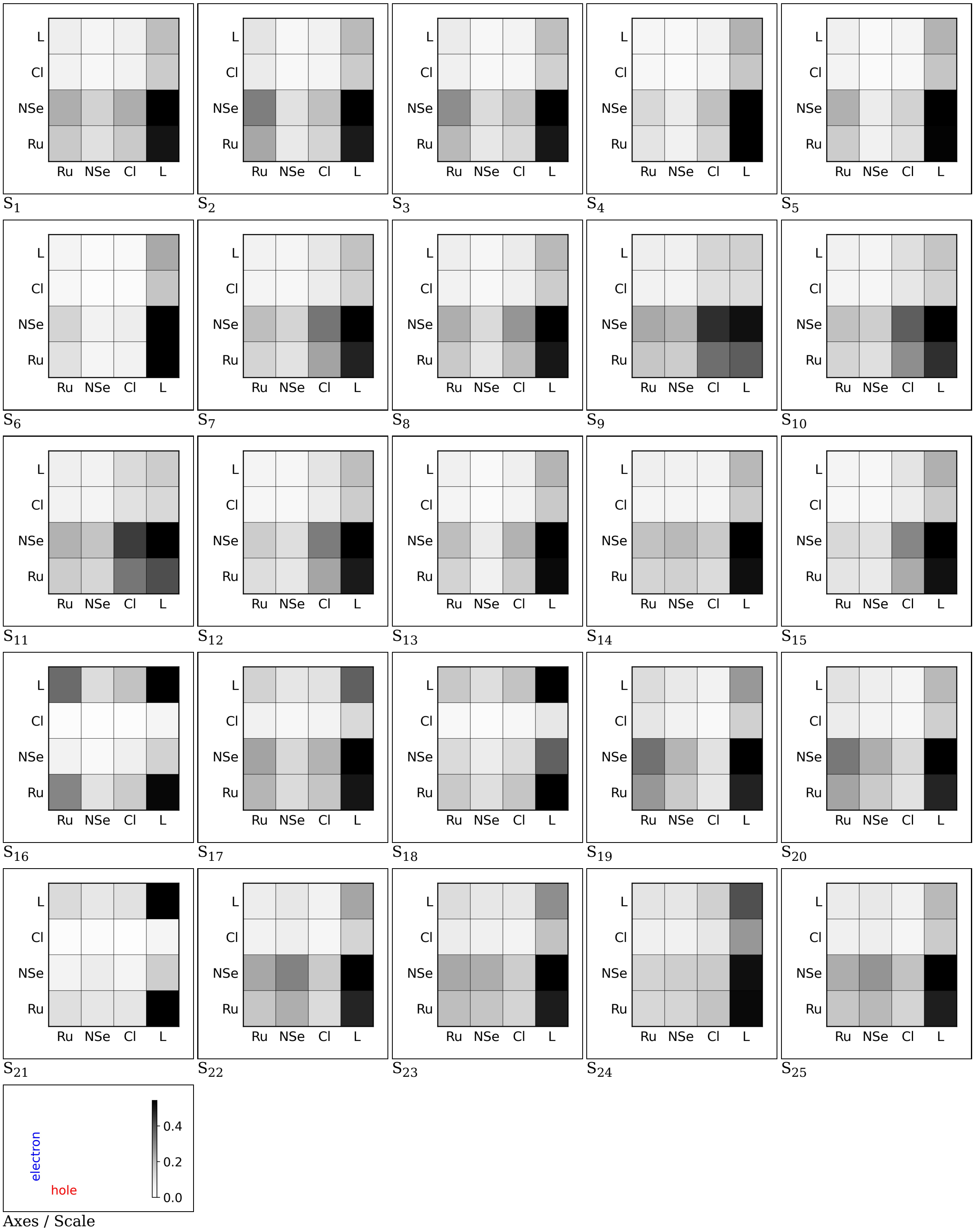


Figure S9: Electron-hole correlation plots of the Omega matrices for the individual states of $\mathbf{[Ru(NSe)]^0}$ in the gas phase.

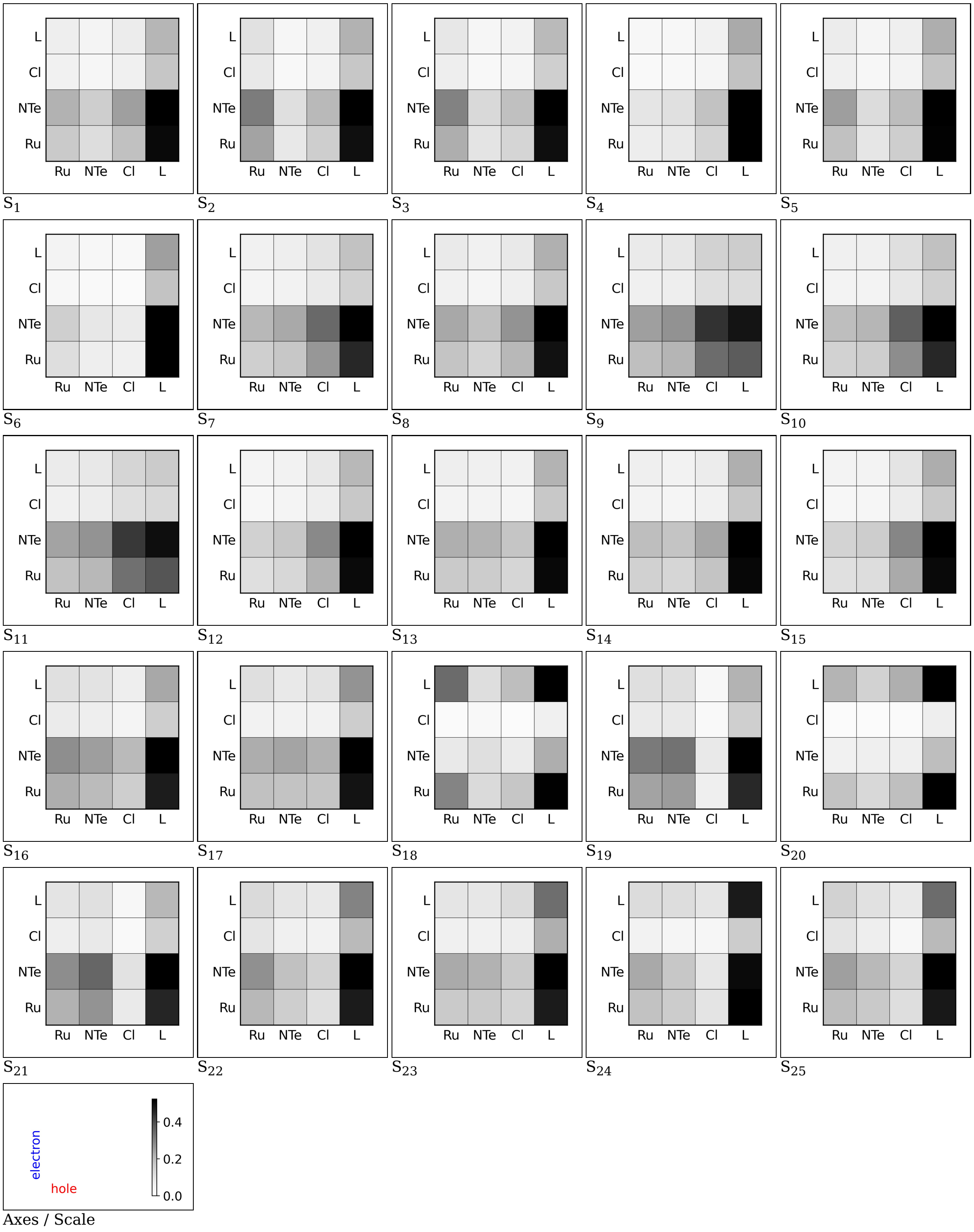


Figure S10: Electron-hole correlation plots of the Omega matrices for the individual states of $[\mathbf{Ru(NTe)}]^{\mathbf{0}}$ in the gas phase.

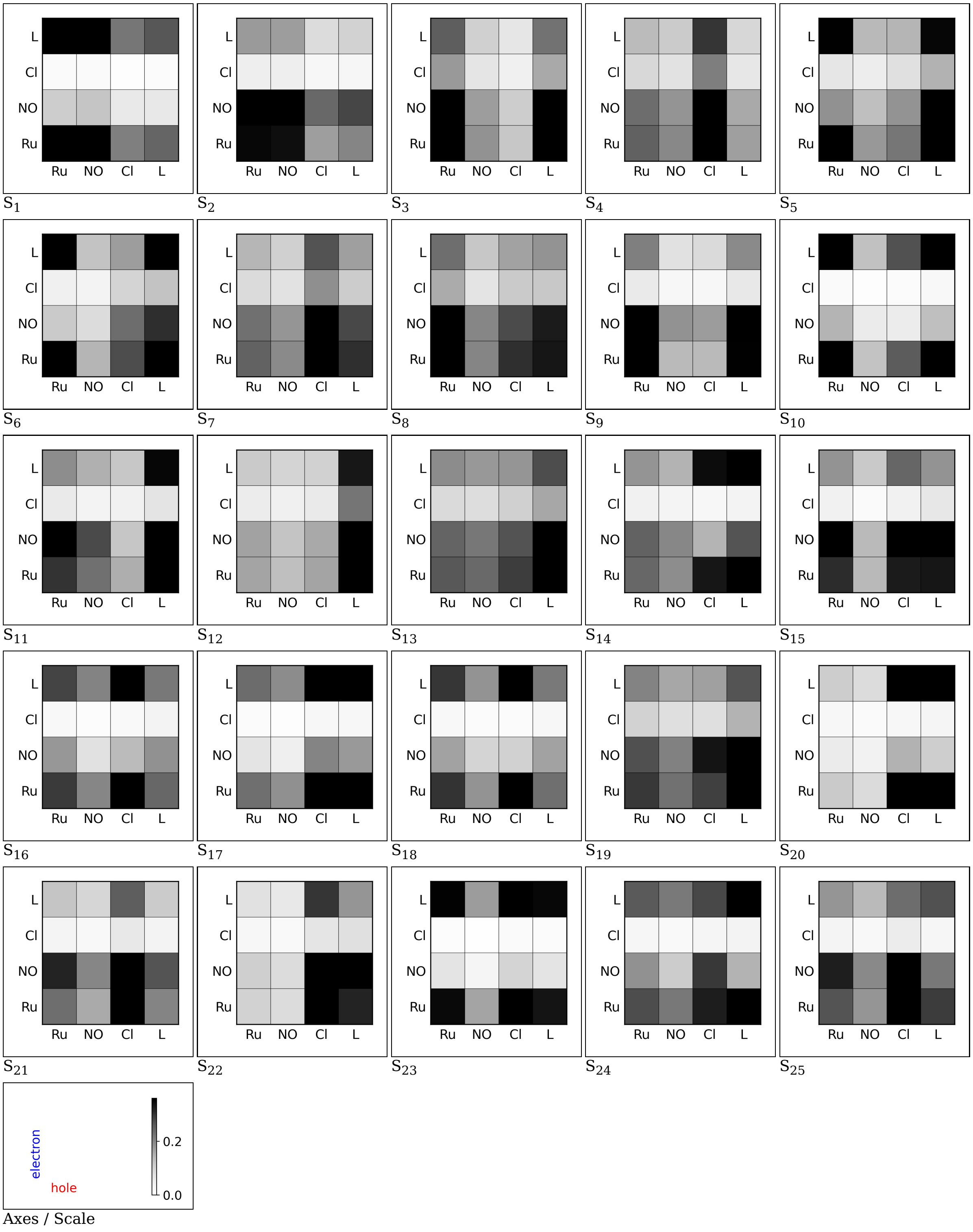


Figure S11: Electron-hole correlation plots of the Omega matrices for the individual states of $[\mathbf{Ru(NO)}]^{-1}$ in the gas phase.

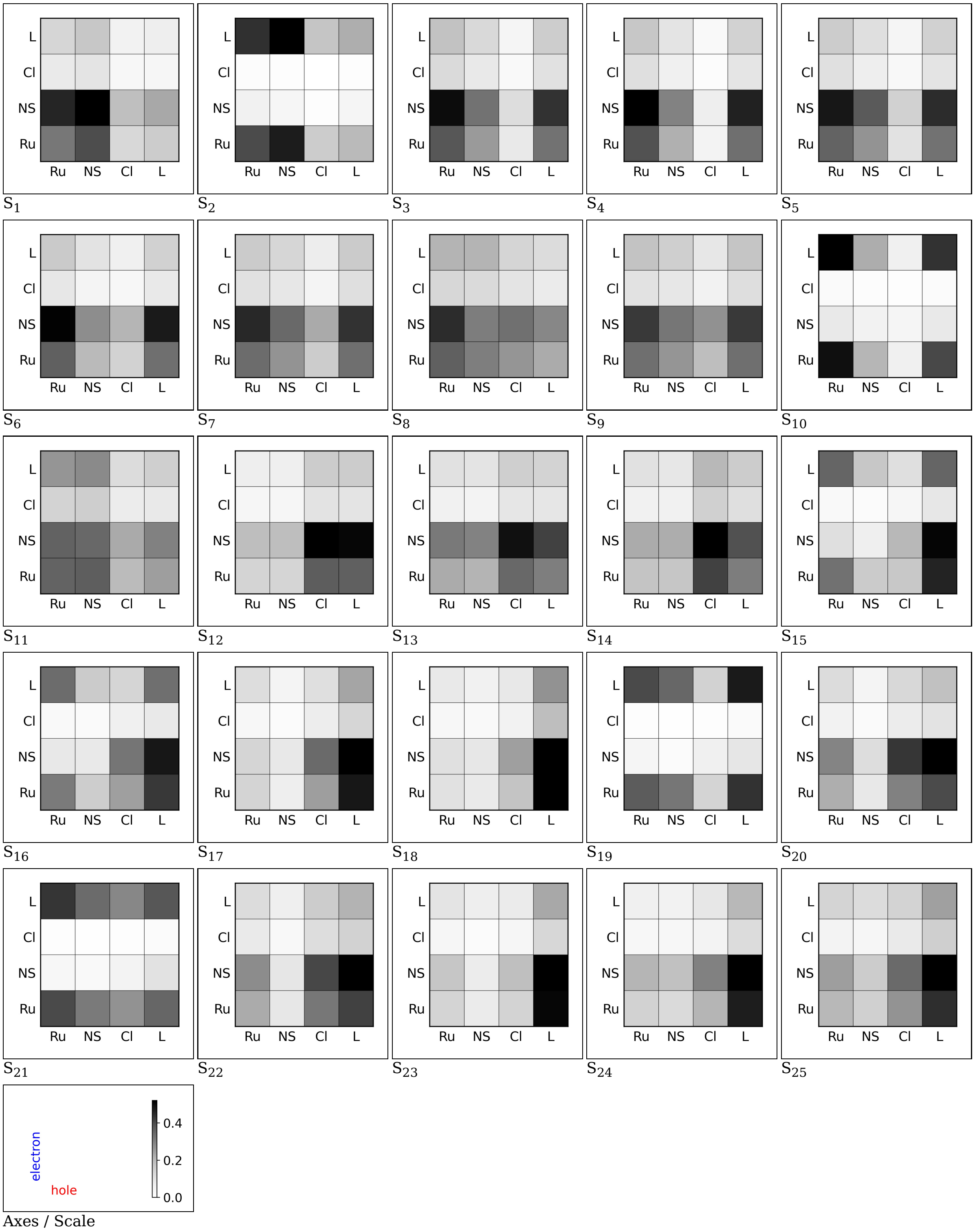


Figure S12: Electron-hole correlation plots of the Omega matrices for the individual states of $[\mathbf{Ru(NS)}]^{-1}$ in the gas phase.

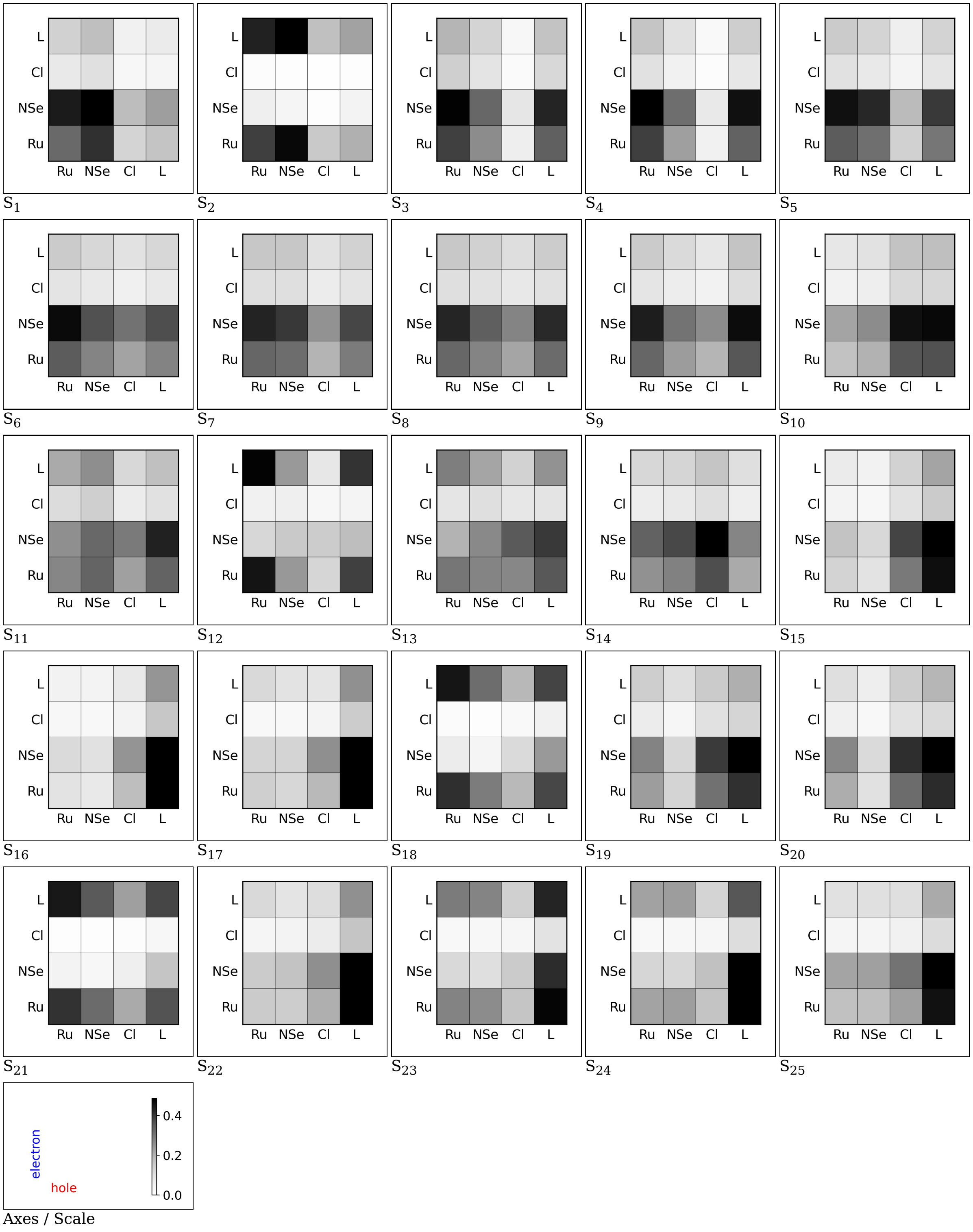


Figure S13: Electron-hole correlation plots of the Omega matrices for the individual states of $[\mathbf{Ru(NSe)}]^{-1}$ in the gas phase.

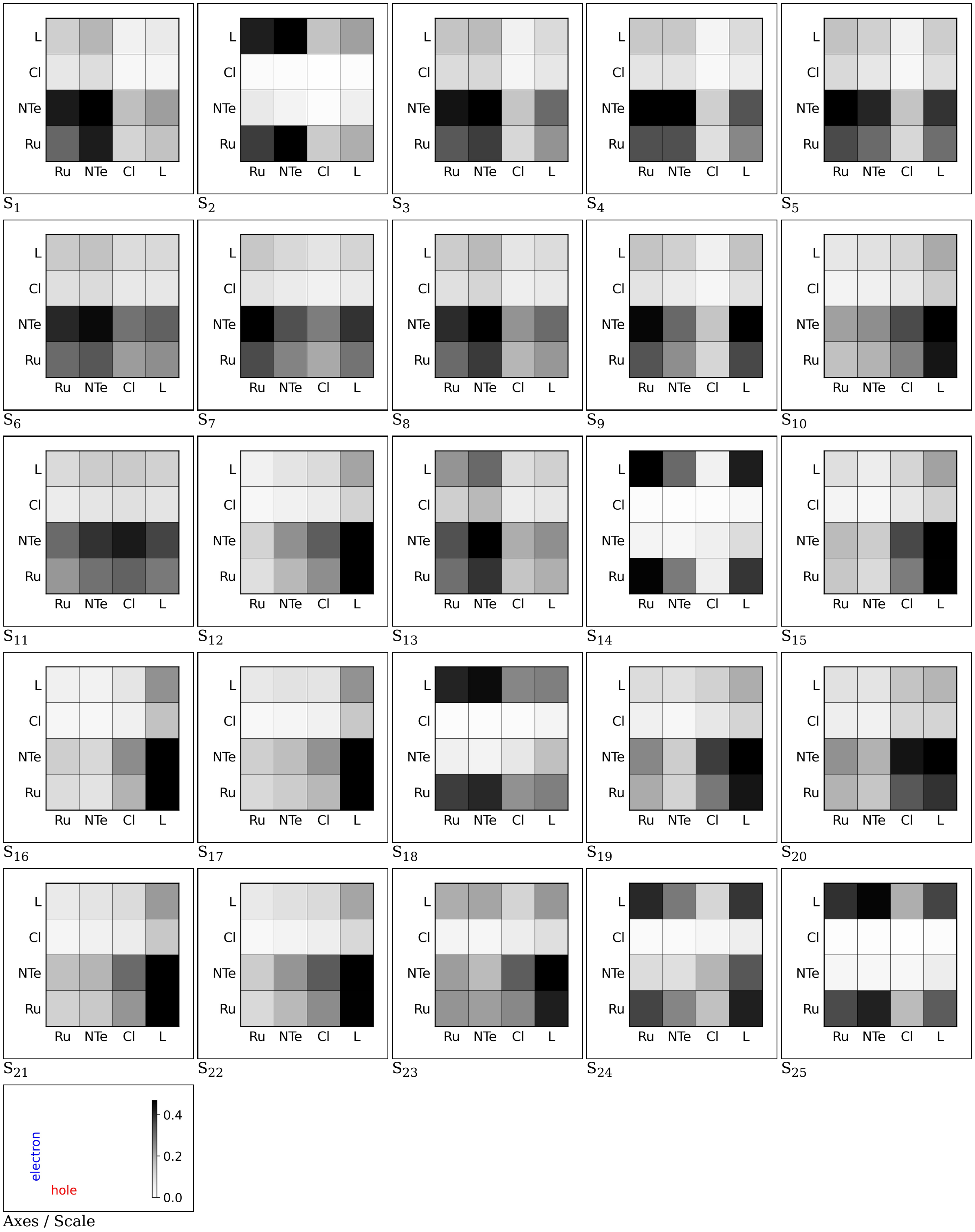


Figure S14: Electron-hole correlation plots of the Omega matrices for the individual states of $[\mathbf{Ru(NTe)}]^{-\mathbf{1}}$ in the gas phase.

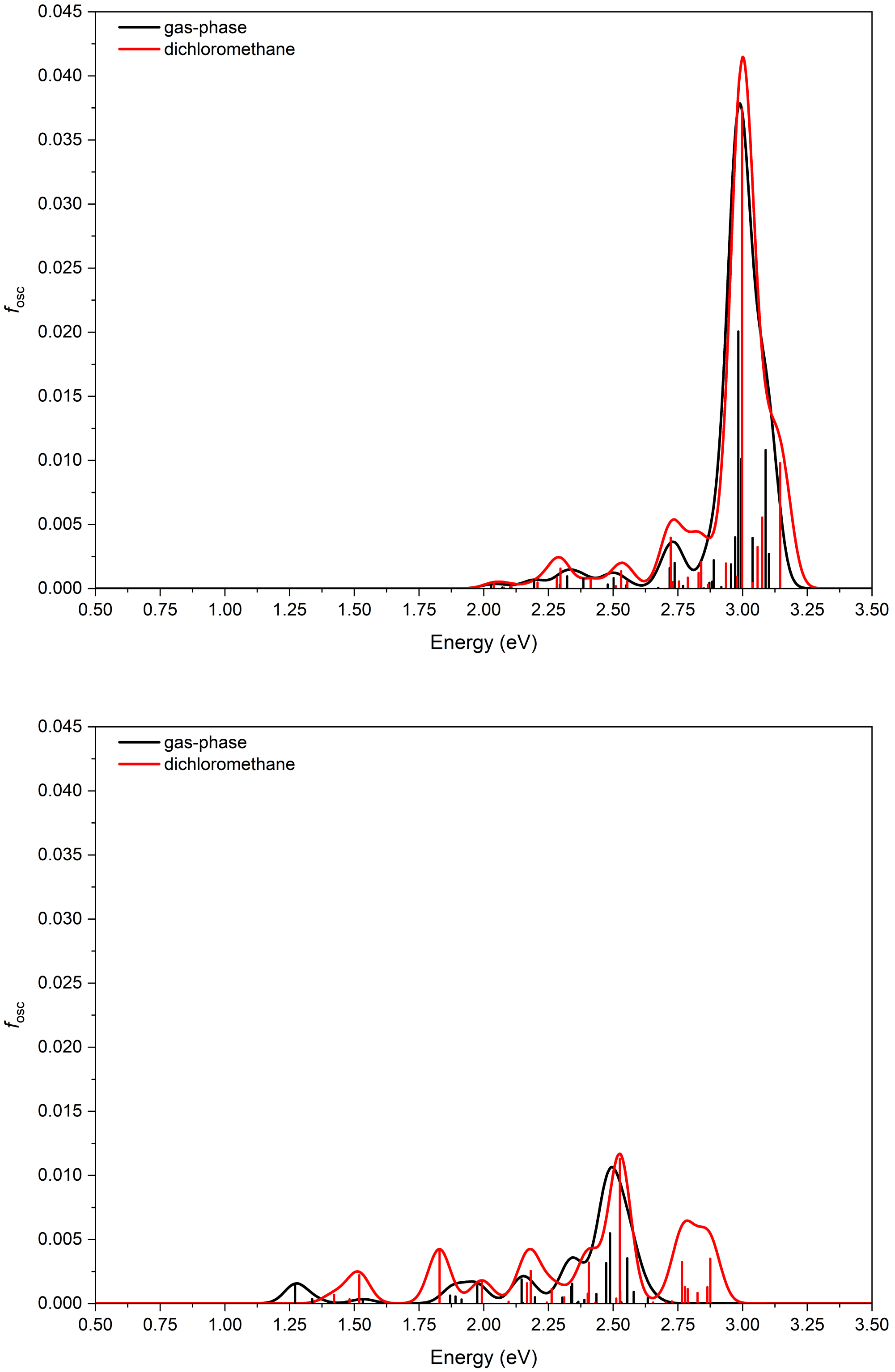


Figure S15: Calculated spectra for $[\mathbf{Ru(NO)}]^{\mathbf{0}}$ (top) and $[\mathbf{Ru(NO)}]^{-\mathbf{1}}$ (bottom) in the gas phase (black) and SMD(dichloromethane) (red).

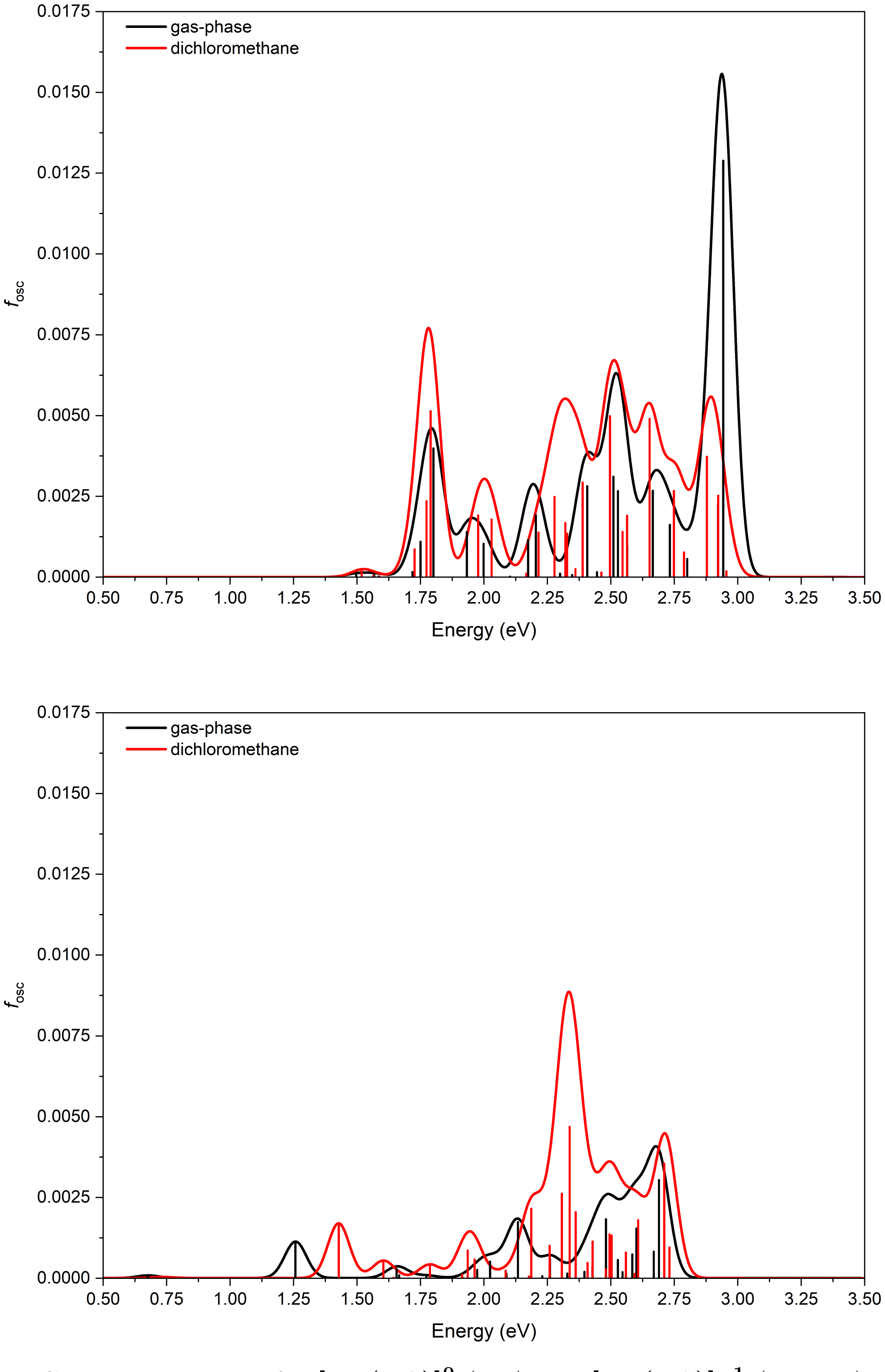


Figure S16: Calculated spectra for $[\mathbf{Ru(NS)}]^{\mathbf{0}}$ (top) and $[\mathbf{Ru(NS)}]^{-\mathbf{1}}$ (bottom) in the gas phase (black) and SMD(dichloromethane) (red).

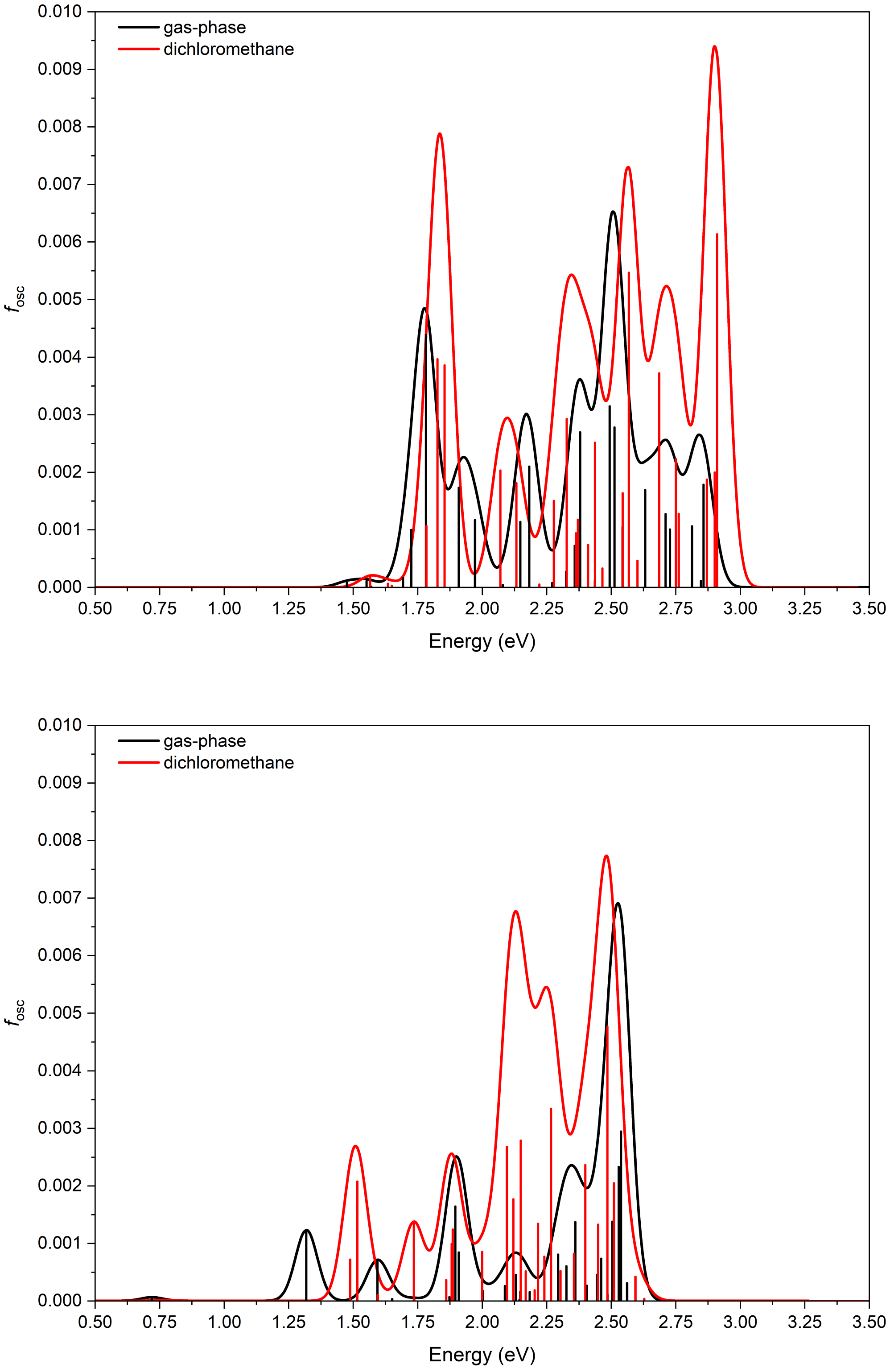


Figure S17: Calculated spectra for $[\mathbf{Ru(NSe)}]^{\mathbf{0}}$ (top) and $[\mathbf{Ru(NSe)}]^{\mathbf{-1}}$ (bottom) in the gas phase (black) and SMD(dichloromethane) (red).

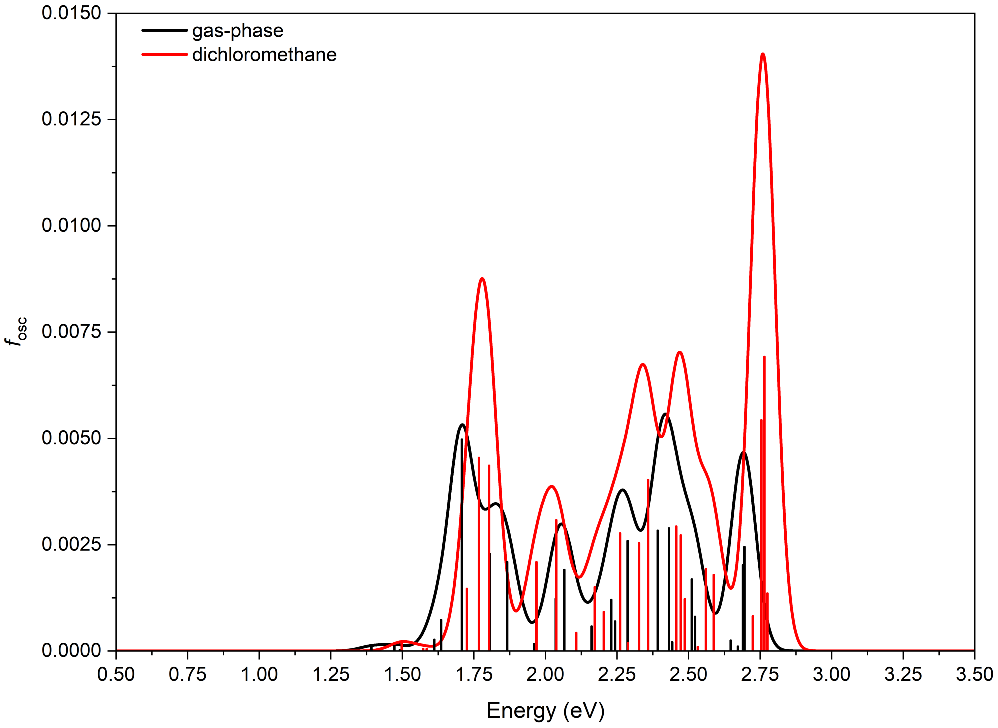


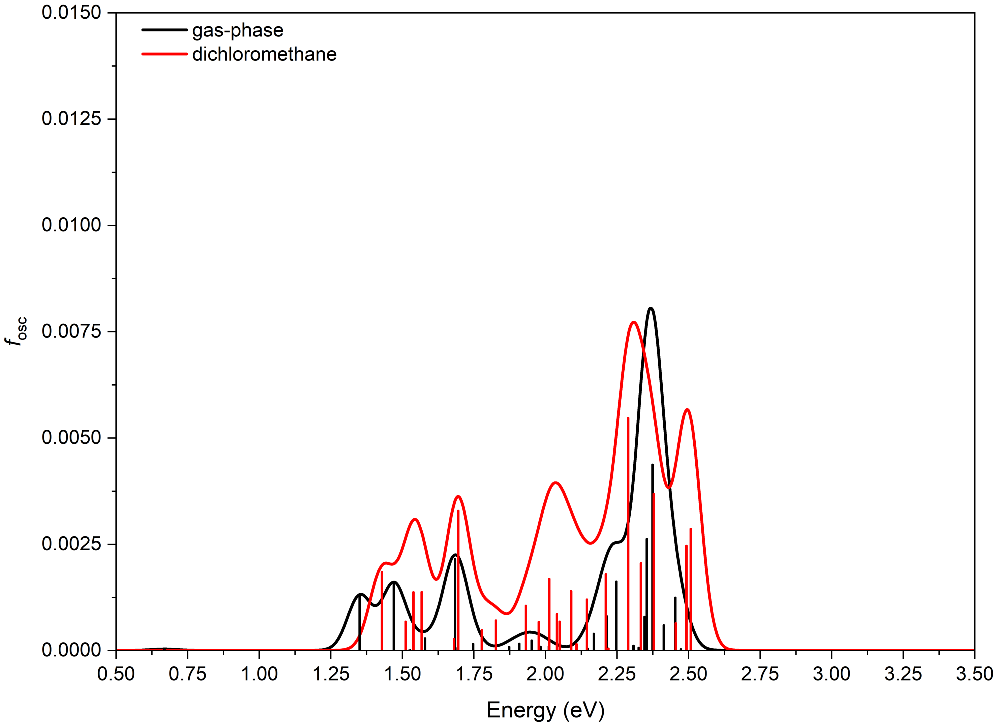


Figure S18: Calculated spectra for $\mathbf{[Ru(NTe)]^{0}}$ (top) and $\mathbf{[Ru(NTe)]^{-1}}$ (bottom) in the gas phase (black) and SMD(dichloromethane) (red).

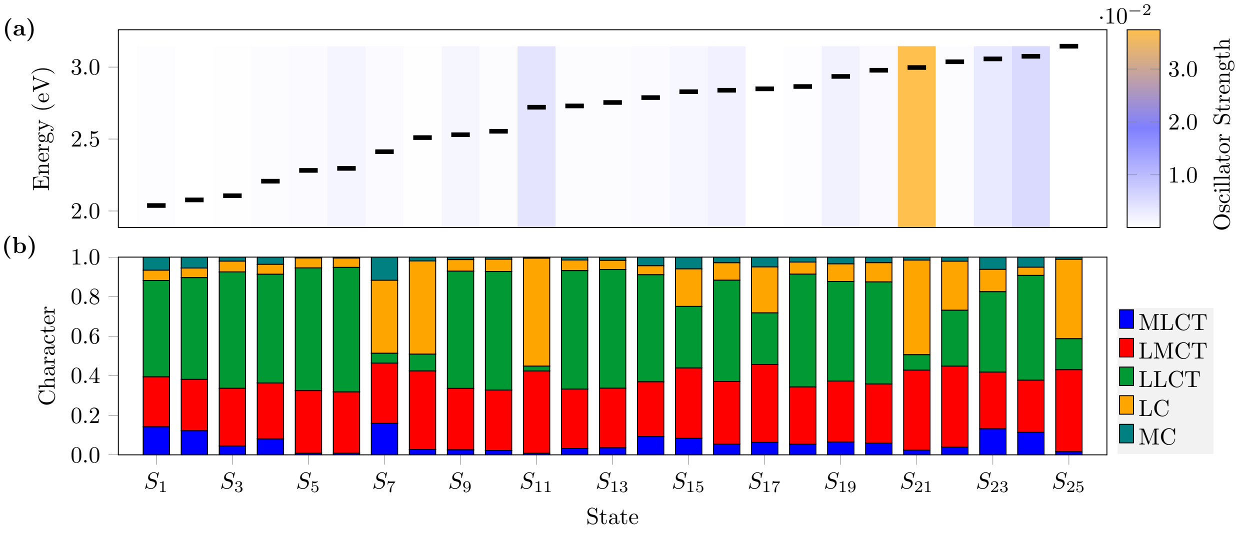


Figure S19: (a)Excitation energies and oscillator strength; (b) decomposition of the charge transfer number matrices of $[\mathbf{Ru(NO)}]^{\mathbf{0}}$ in the SMD(dichloromethane) environment.

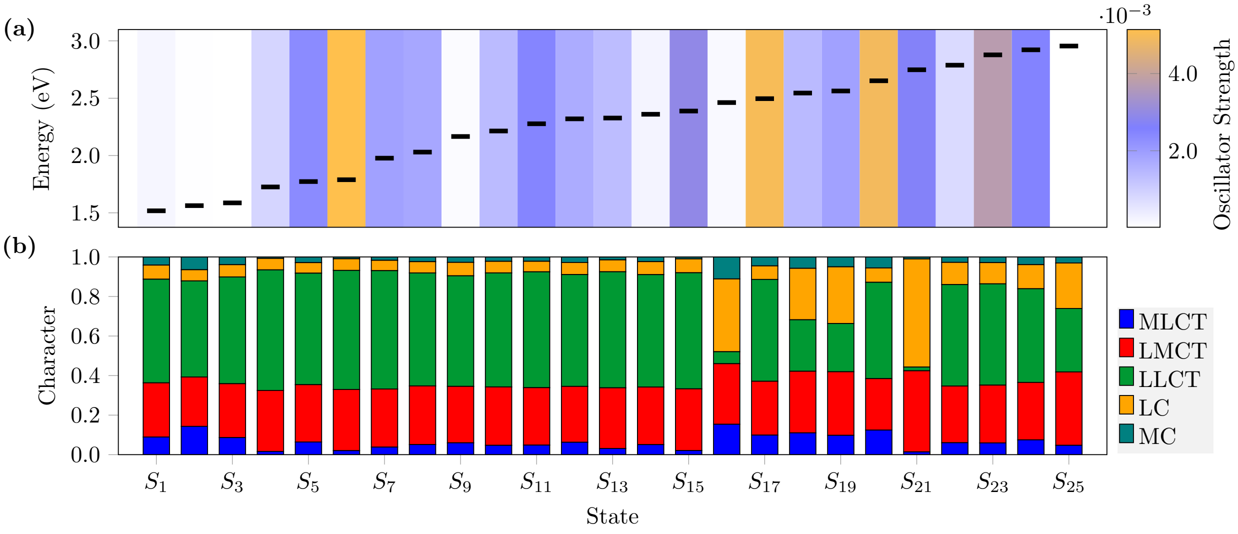


Figure S20: (a)Excitation energies and oscillator strength; (b) decomposition of the charge transfer number matrices of $[\mathbf{Ru(NS)}]^{\mathbf{0}}$ in the SMD(dichloromethane) environment.

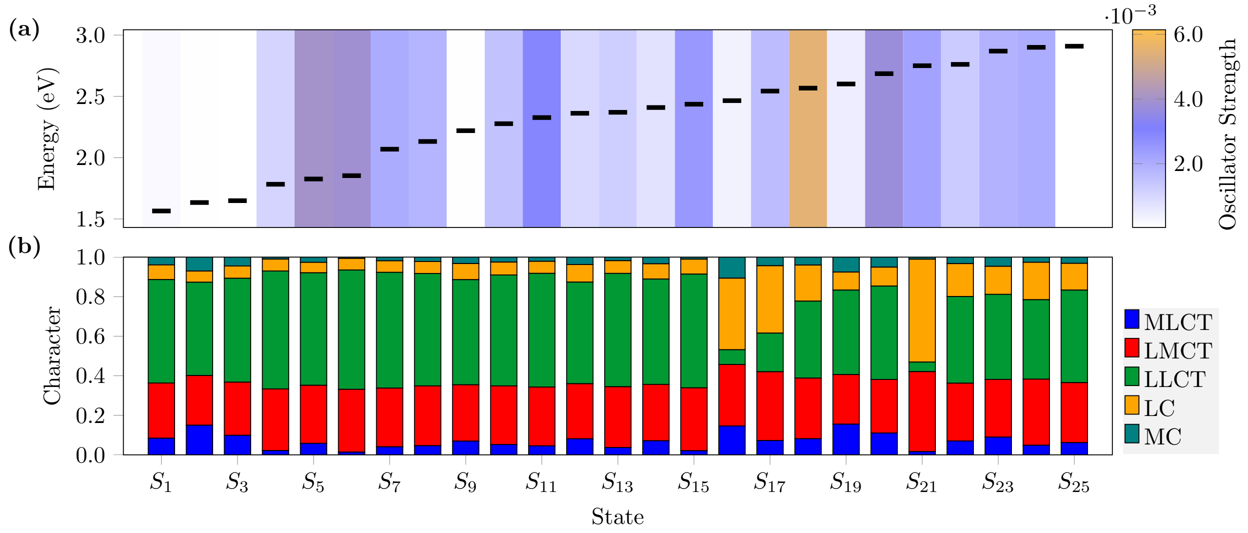


Figure S21: (a)Excitation energies and oscillator strength; (b) decomposition of the charge transfer number matrices of **[Ru(NSe)]$^{\mathbf{0}}$** in the SMD(dichloromethane) environment.

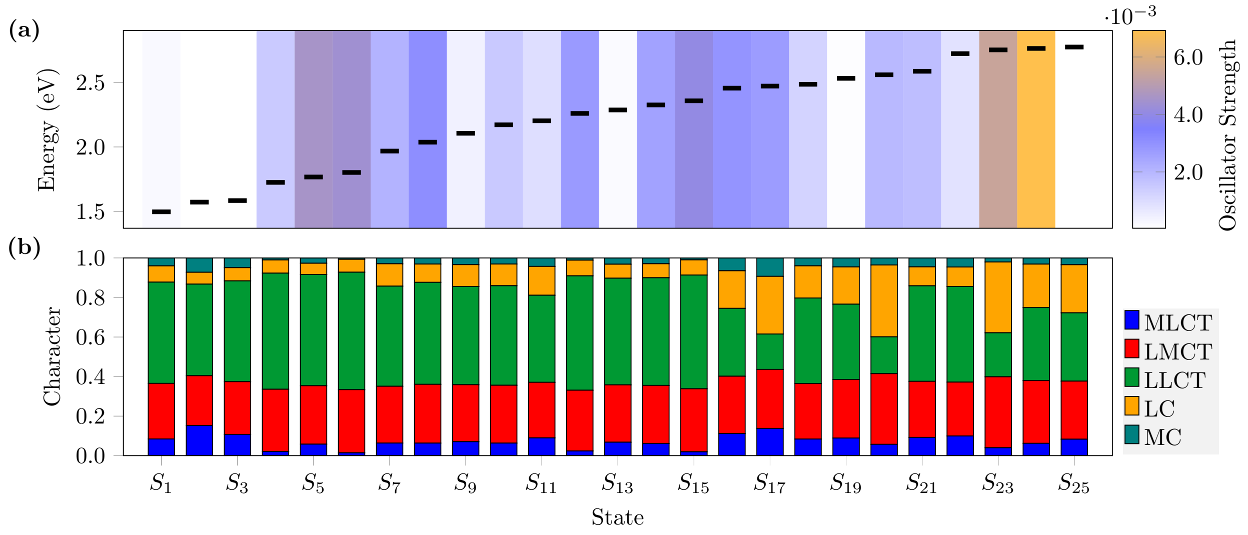


Figure S22: (a)Excitation energies and oscillator strength; (b) decomposition of the charge transfer number matrices of **[Ru(NTe)]$^{\mathbf{0}}$** in the SMD(dichloromethane) environment.

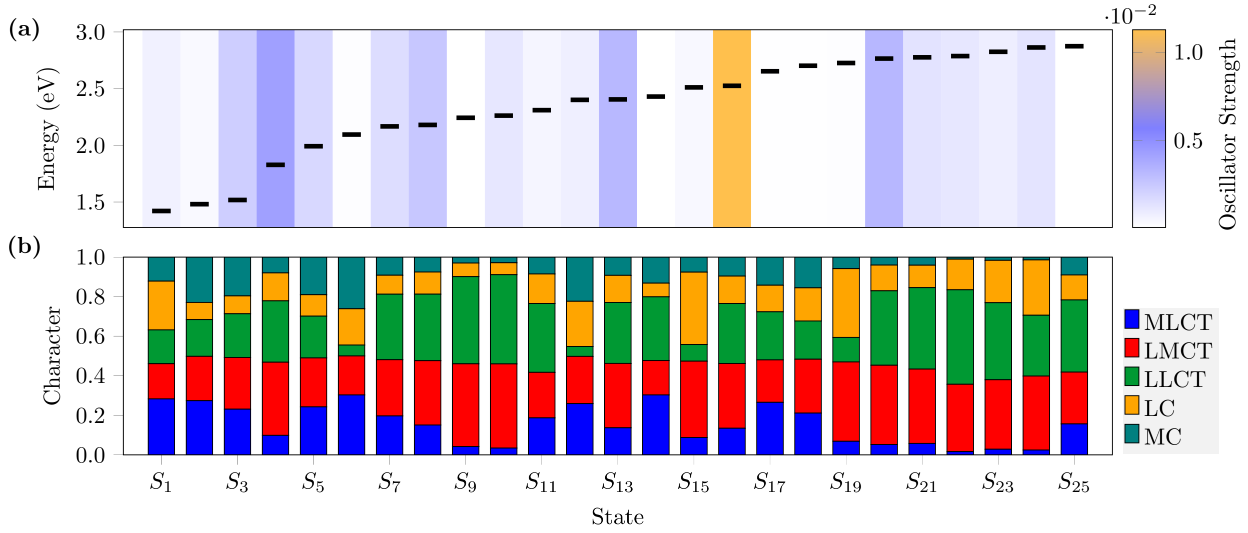


Figure S23: (a)Excitation energies and oscillator strength; (b) decomposition of the charge transfer number matrices of $[\mathbf{Ru(NO)}]^{-1}$ in the SMD(dichloromethane) environment.

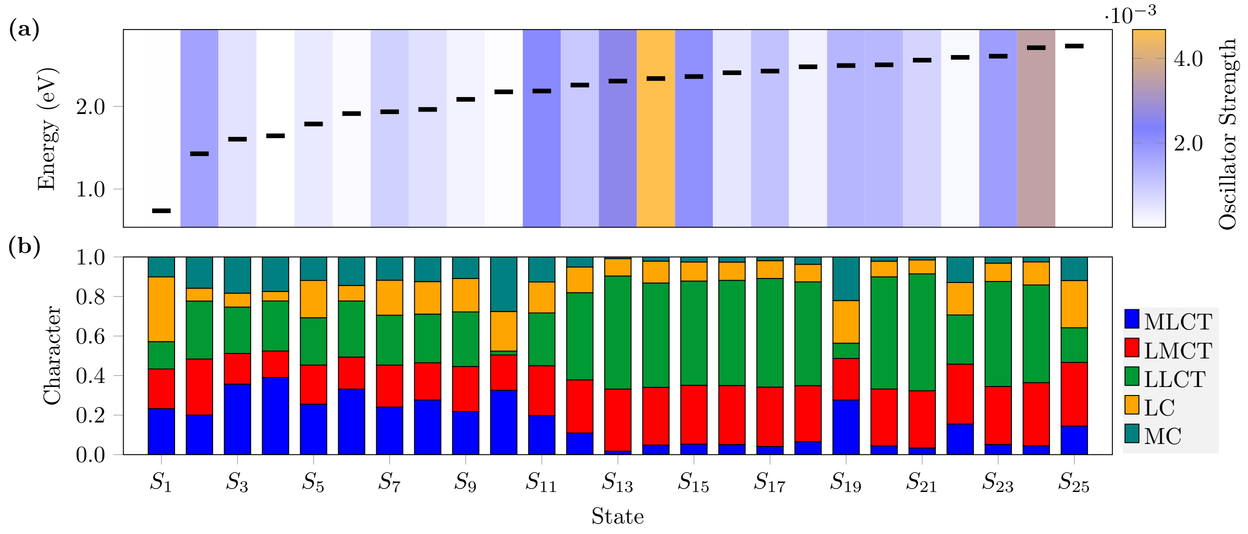


Figure S24: (a)Excitation energies and oscillator strength; (b) decomposition of the charge transfer number matrices of $[\mathbf{Ru(NS)}]^{-1}$ in the SMD(dichloromethane) environment.

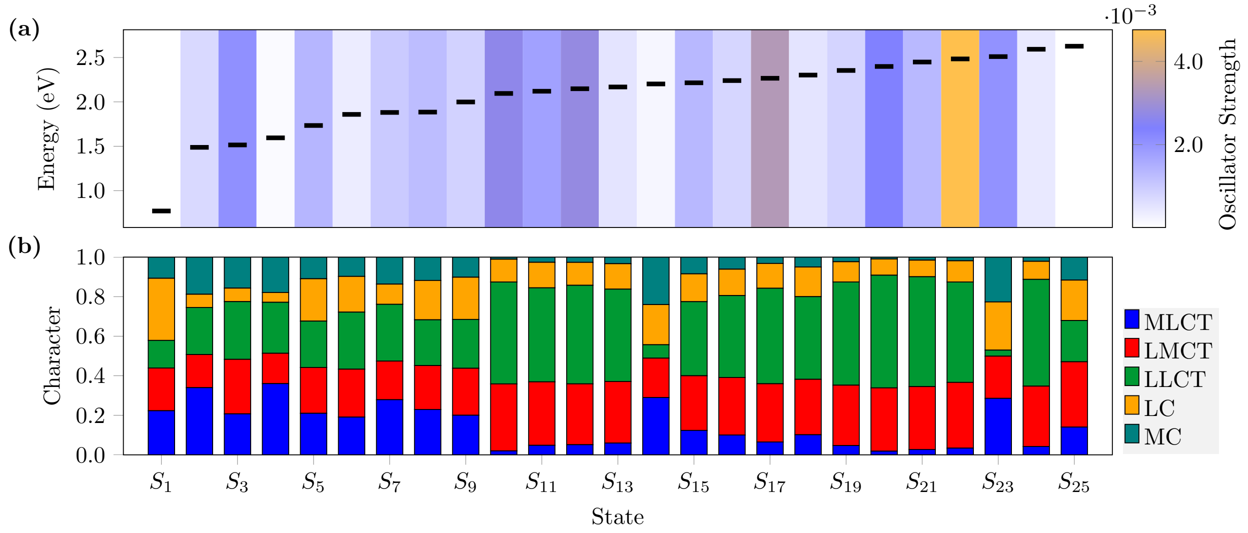


Figure S25: (a)Excitation energies and oscillator strength; (b) decomposition of the charge transfer number matrices of $[\mathbf{Ru(NSe)}]^{-1}$ in the SMD(dichloromethane) environment.

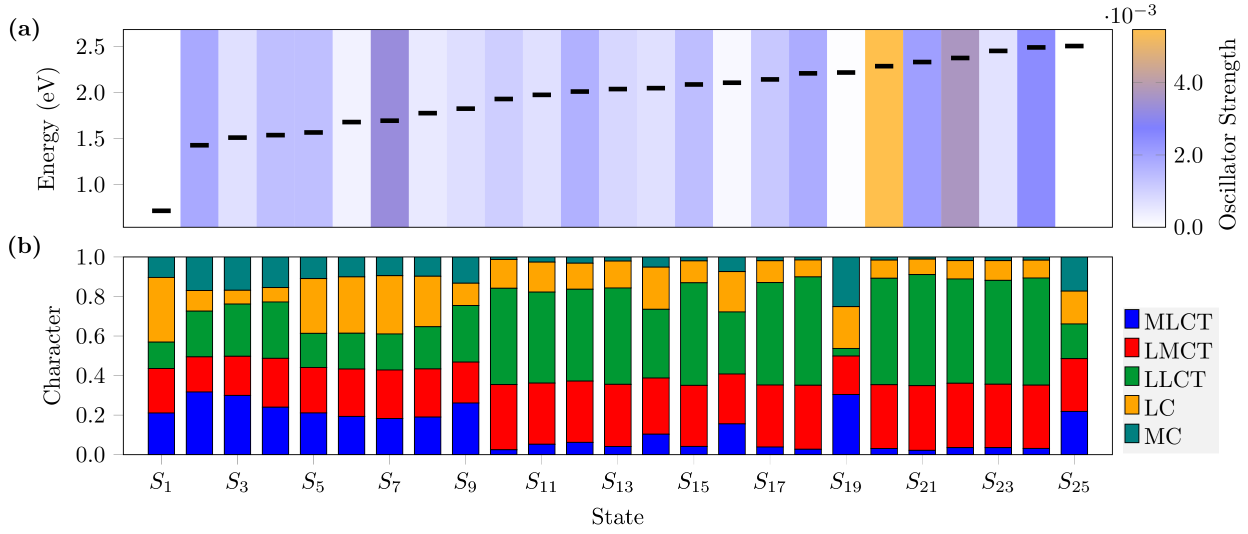


Figure S26: (a)Excitation energies and oscillator strength; (b) decomposition of the charge transfer number matrices of $[\mathbf{Ru(NTe)}]^{-1}$ in the SMD(dichloromethane) environment.

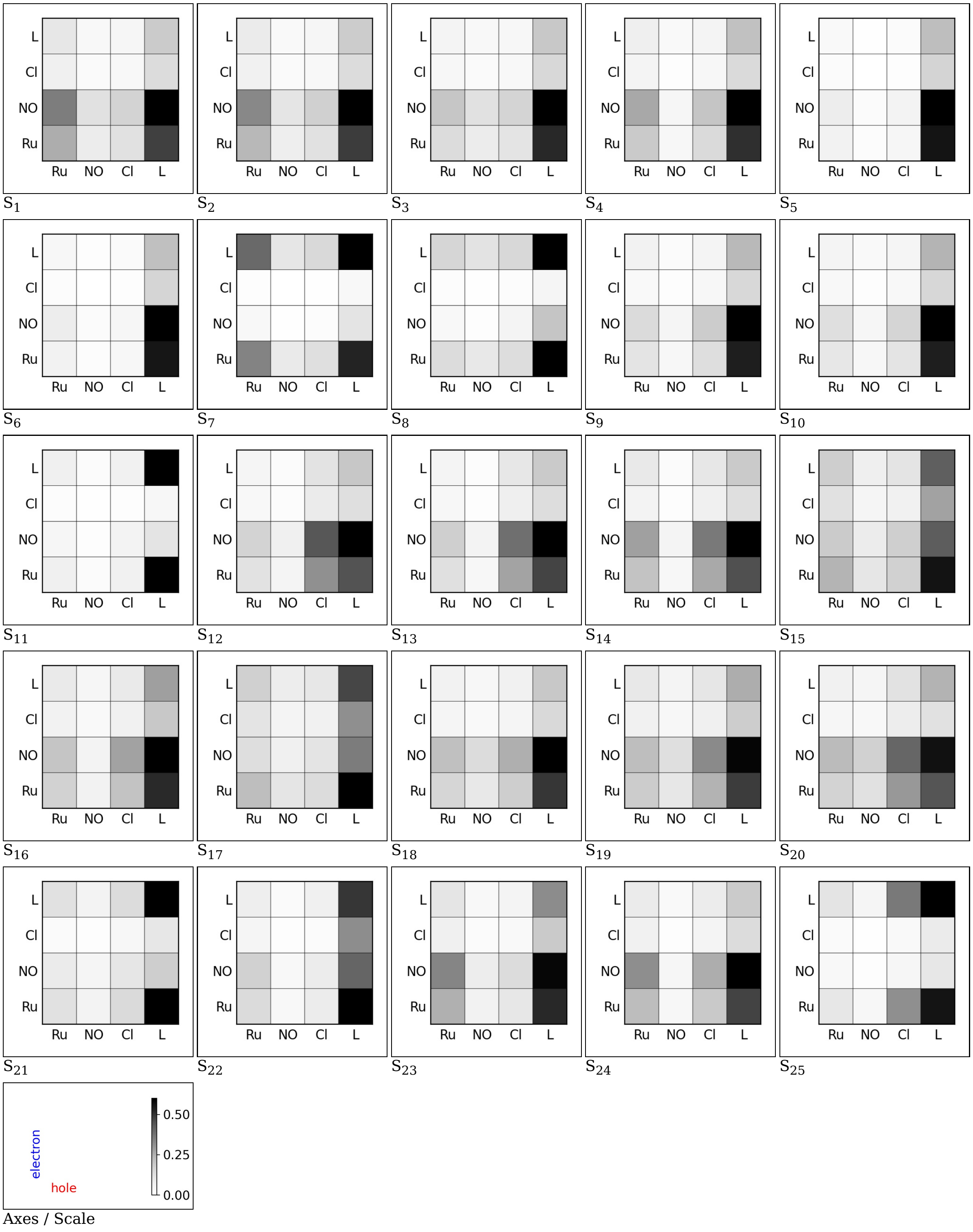


Figure S27: Electron-hole correlation plots of the Omega matrices for the individual states of $[\mathbf{Ru(NO)}]^{\mathbf{0}}$ in the SMD(dichloromethane) environment.

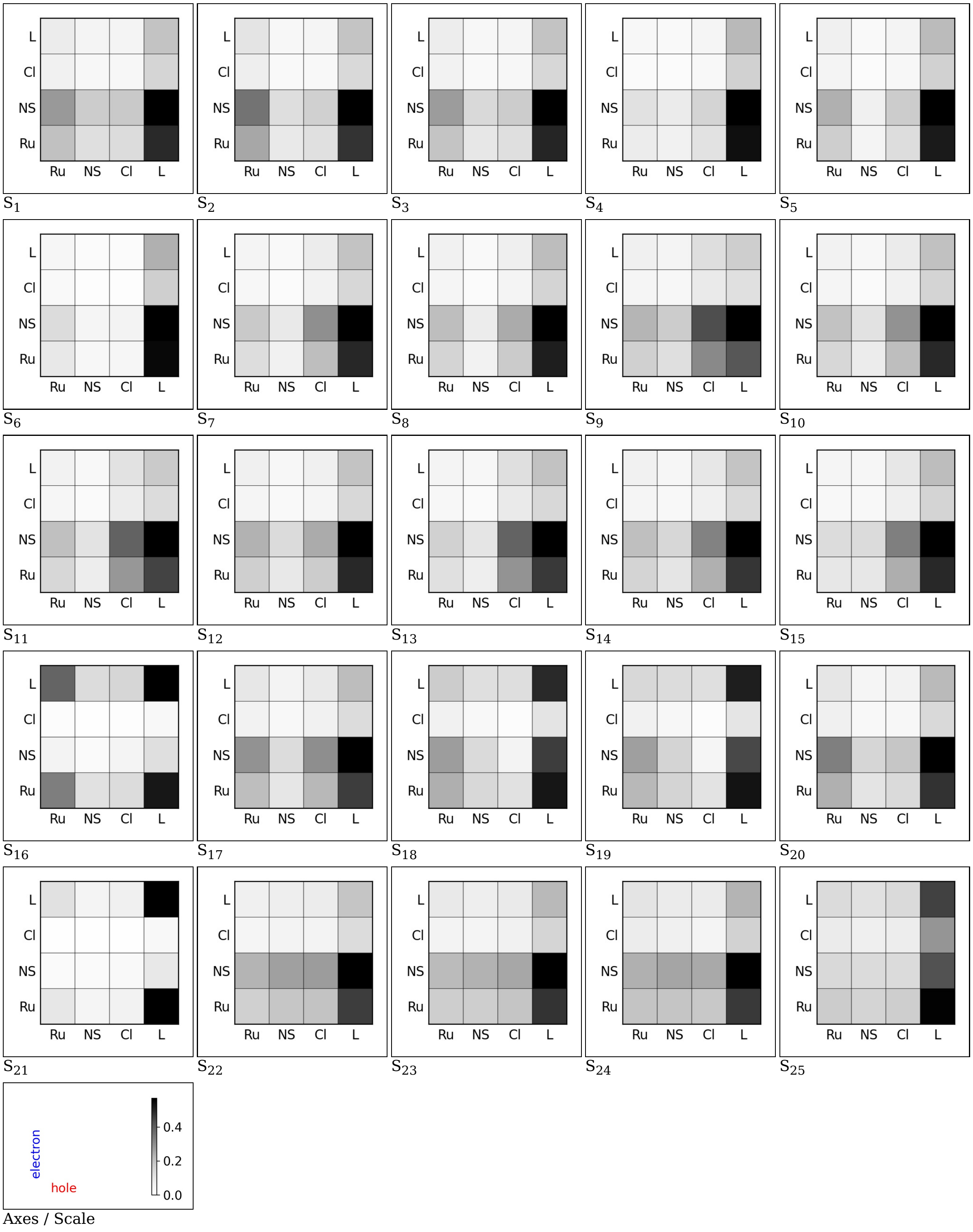


Figure S28: Electron-hole correlation plots of the Omega matrices for the individual states of $[\mathbf{Ru(NS)}]^{\mathbf{0}}$ in the SMD(dichloromethane) environment.

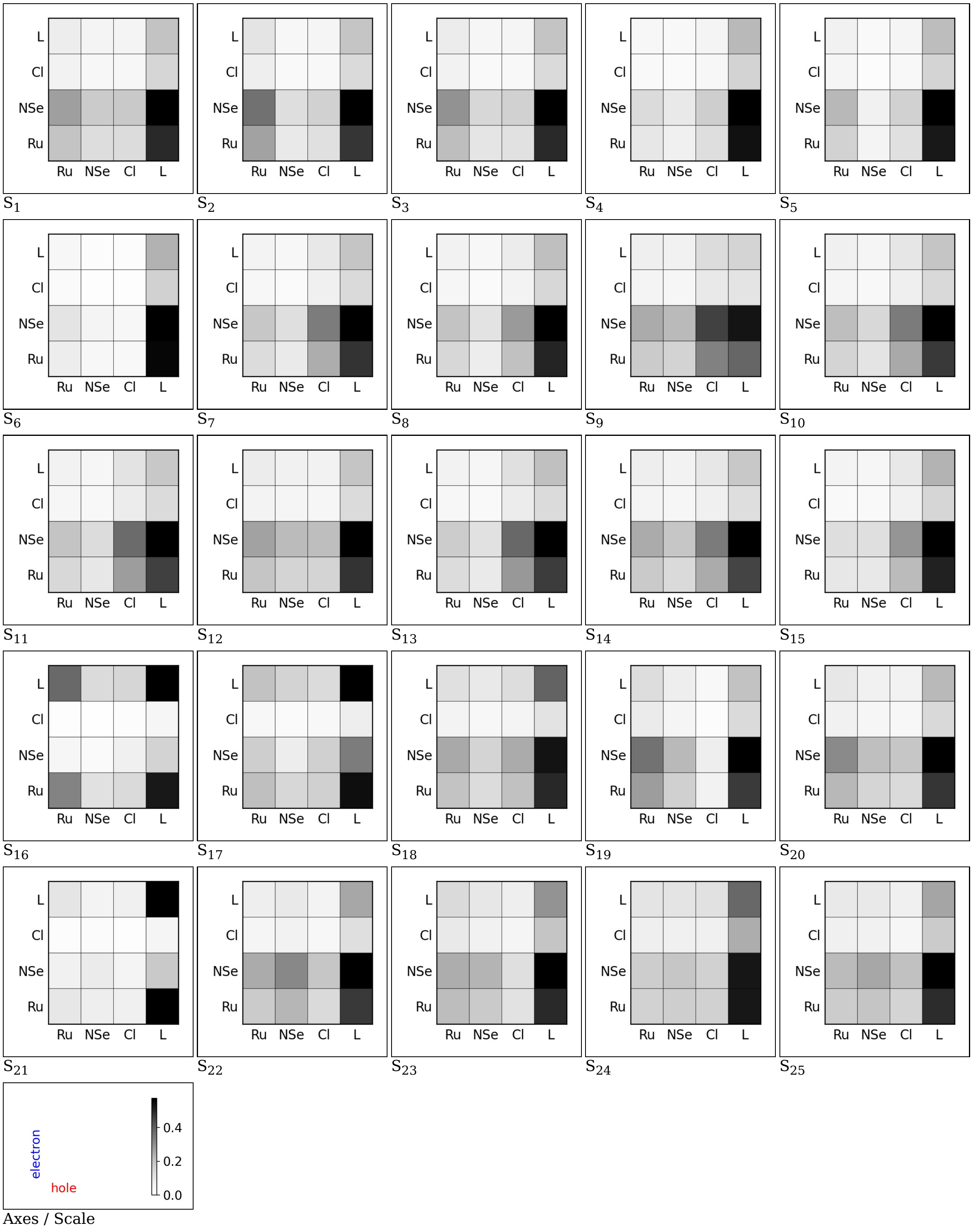


Figure S29: Electron-hole correlation plots of the Omega matrices for the individual states of $[\mathbf{Ru(NSe)}]^{\mathbf{0}}$ in the SMD(dichloromethane) environment.

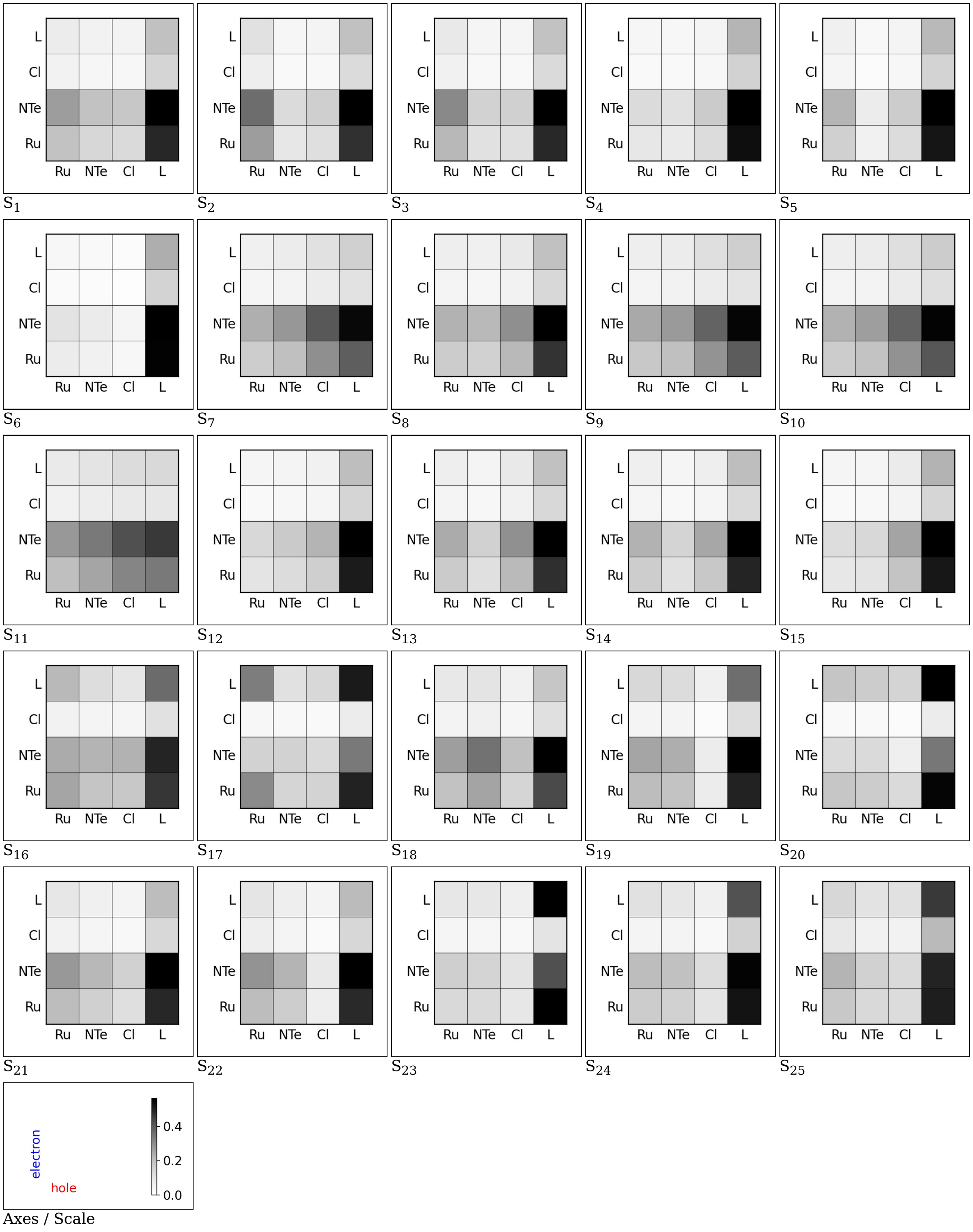


Figure S30: Electron-hole correlation plots of the Omega matrices for the individual states of $[\mathbf{Ru(NTe)}]^{\mathbf{0}}$ in the SMD(dichloromethane) environment.

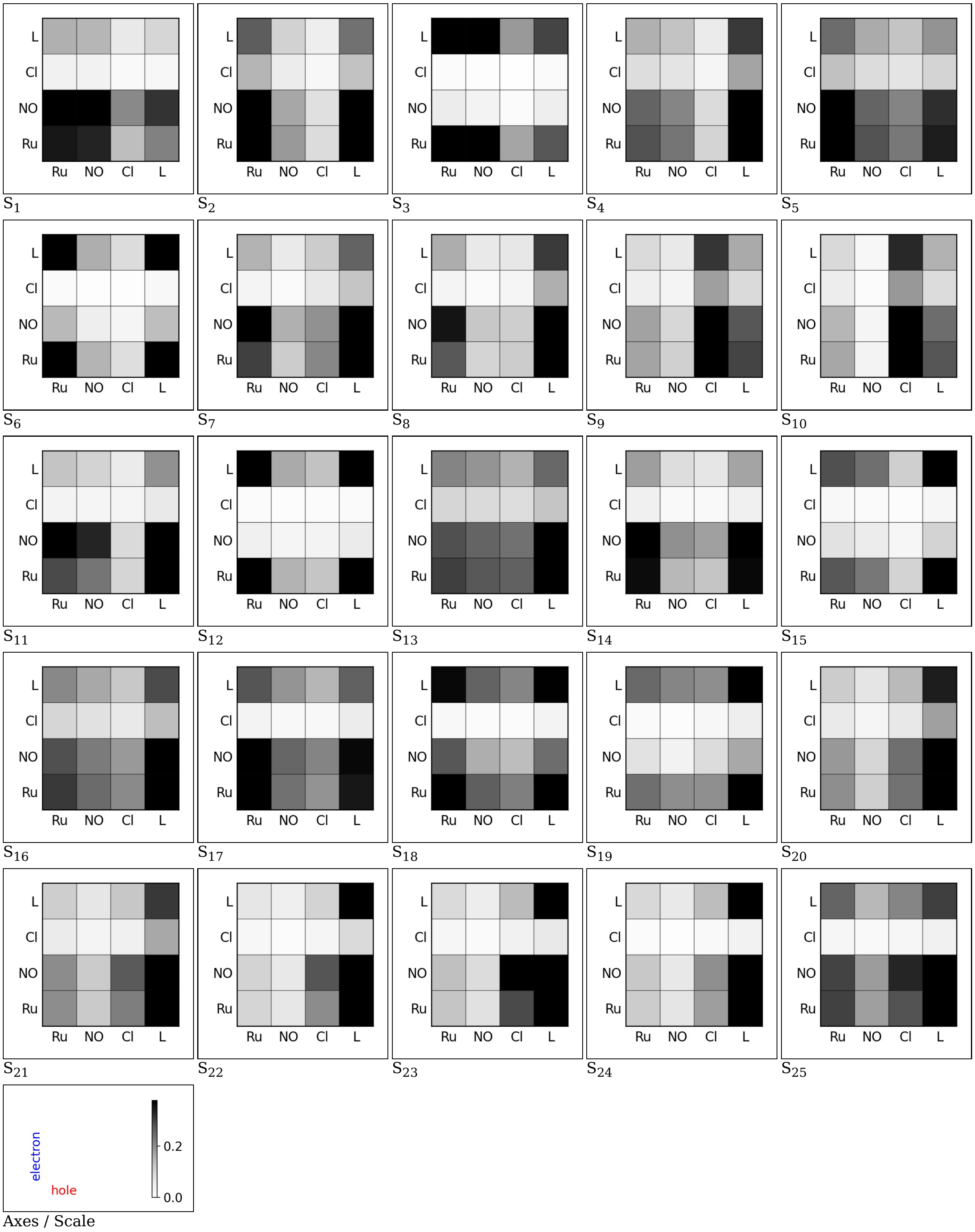


Figure S31: Electron-hole correlation plots of the Omega matrices for the individual states of $[\mathbf{Ru(NO)}]^{-1}$ in the SMD(dichloromethane) environment.

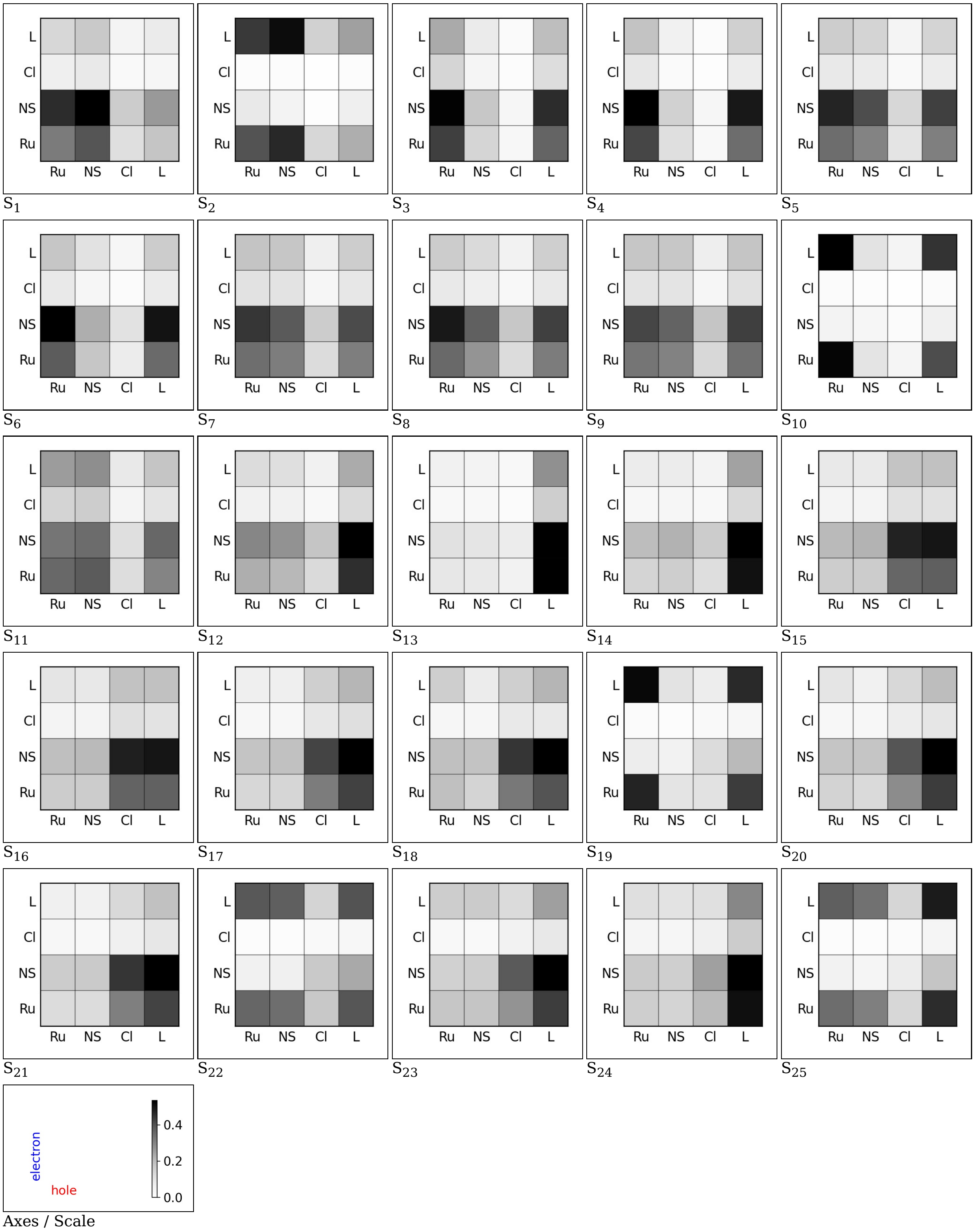


Figure S32: Electron-hole correlation plots of the Omega matrices for the individual states of $[\mathbf{Ru(NS)}]^{-1}$ in the SMD(dichloromethane) environment.

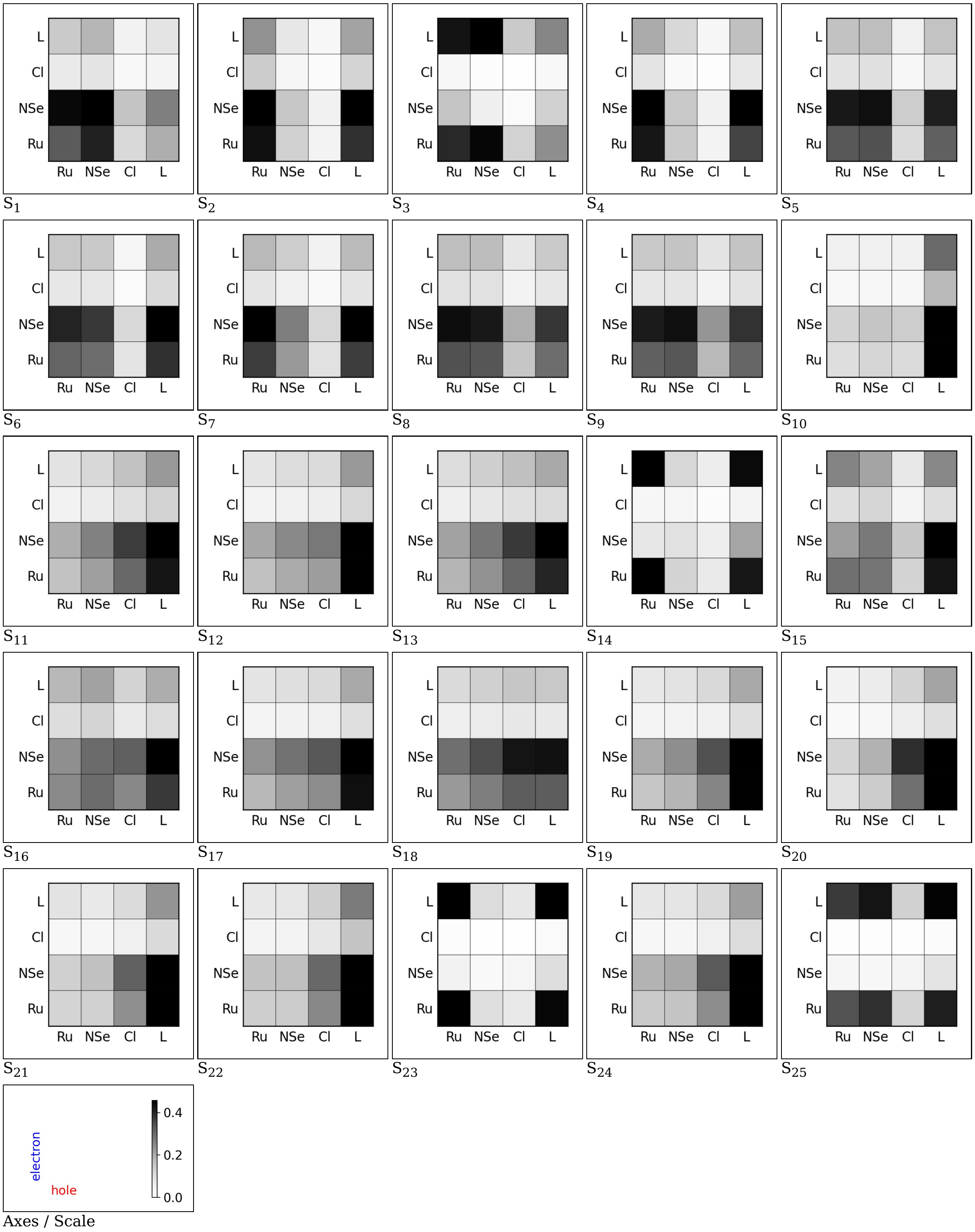


Figure S33: Electron-hole correlation plots of the Omega matrices for the individual states of $[\mathbf{Ru(NSe)}]^{-1}$ in the SMD(dichloromethane) environment.

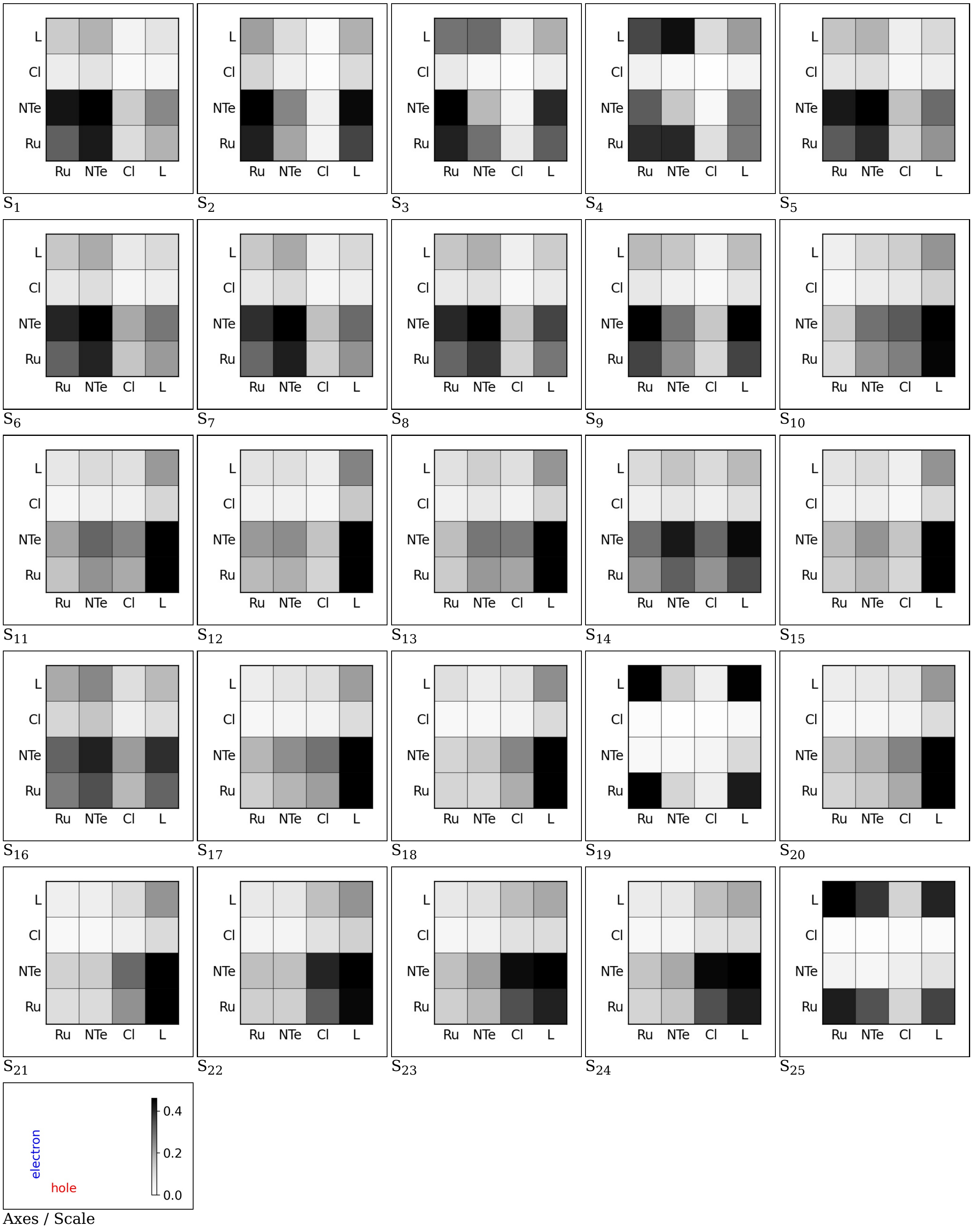


Figure S34: Electron-hole correlation plots of the Omega matrices for the individual states of $[\mathbf{Ru(NTe)}]^{-1}$ in the SMD(dichloromethane) environment.

# Coordinates Cartesian

$[Ru(NO)]^0$

N 2.032667000 1.972208000 5.656917000

O 0.945196000 1.650919000 5.393831000

Cl 5.970803000 2.838275000 6.446971000

Ru 3.693835000 2.386623000 6.017886000

S 4.430178000 1.711779000 3.752113000

S 3.319028000 4.791863000 5.597646000

S 4.245659000 0.021256000 6.501241000

S 3.134523000 3.128686000 8.307623000

P 3.573018000 3.021313000 2.435528000

P 4.332973000 5.329958000 3.896901000

P 3.210482000 -0.492599000 8.188172000

P 3.963424000 1.819101000 9.652436000

N 3.978404000 4.575445000 2.517055000

N 3.553392000 0.262777000 9.565664000

C 3.392781000 2.426453000 11.265386000

H 2.297769000 2.391855000 11.289885000

H 3.796394000 1.769954000 12.047425000

H 3.733449000 3.456088000 11.426046000

C 5.768891000 1.965946000 9.684660000

H 6.150556000 1.342326000 10.504368000

H 6.156732000 1.608321000 8.723720000

H 6.069442000 3.011847000 9.815709000

C 1.413536000 -0.402682000 7.906997000

H 1.128509000 -0.896271000 6.971150000

H 0.905499000 -0.873324000 8.759442000

H 1.130641000 0.655737000 7.876022000

C 3.577373000 -2.251804000 8.438363000

H 3.077313000 -2.587621000 9.356022000

H 3.230559000 -2.839875000 7.580548000

H 4.661059000 -2.370591000 8.551186000

C 1.757003000 2.894234000 2.471658000

H 1.353120000 3.458596000 1.620405000

H 1.426782000 1.850229000 2.430476000

H 1.405284000 3.364368000 3.397036000

C 4.070566000 2.421098000 0.796970000

H 5.165272000 2.415196000 0.743953000

H 3.689098000 1.406998000 0.629423000

H 3.673526000 3.106416000 0.036914000

C 6.124868000 5.294399000 4.163085000

H 6.394148000 5.861945000 5.061247000

H 6.427413000 4.250430000 4.305526000

H 6.613279000 5.713183000 3.272803000

C 3.904485000 7.078418000 3.663796000

H 4.411622000 7.444304000 2.761388000

H 2.819904000 7.162988000 3.531966000

H 4.217466000 7.665319000 4.535328000

[Ru(NS)]$^0$

N 1.789292000 2.051078000 5.566475000

S 0.316563000 1.645530000 5.493462000

Cl 5.811099000 2.419735000 6.435377000

Ru 3.505485000 2.309048000 5.859323000

S 4.235531000 1.680269000 3.602549000

S 3.473174000 4.745495000 5.595823000

S 3.729175000 -0.131224000 6.121472000

S 2.859449000 2.990726000 8.157142000

P 3.471671000 3.109303000 2.351086000

P 4.526379000 5.219407000 3.899239000

P 3.396618000 -0.524017000 8.097917000

P 3.950129000 1.901528000 9.504184000

N 4.030708000 4.612150000 2.488656000

N 4.181662000 0.339682000 9.202741000

C 3.016196000 2.099770000 11.058875000

H 2.021378000 1.656801000 10.935171000

H 3.553196000 1.576041000 11.861193000

H 2.910966000 3.161784000 11.312892000

C 5.560233000 2.655129000 9.843833000

H 6.062793000 2.078005000 10.631527000

H 6.145129000 2.625313000 8.917661000

H 5.421890000 3.696164000 10.159601000

C 1.598573000 -0.523478000 8.408429000

H 1.089719000 -1.188138000 7.700534000

H 1.403166000 -0.843479000 9.441169000

H 1.235919000 0.502727000 8.268082000

C 3.939133000 -2.238901000 8.326502000

H 3.702486000 -2.558319000 9.349613000

H 3.442066000 -2.893627000 7.601054000

H 5.023580000 -2.277033000 8.173000000

C 1.655989000 3.147670000 2.441516000

H 1.280934000 3.802601000 1.643702000

H 1.231722000 2.141939000 2.346216000

H 1.380335000 3.578534000 3.410727000

C 3.869929000 2.532990000 0.677274000

H 4.957442000 2.425294000 0.593155000

H 3.388799000 1.567531000 0.481093000

H 3.522603000 3.283600000 -0.044517000

C 6.290474000 4.876739000 4.136400000

H 6.657522000 5.361769000 5.048248000

H 6.417116000 3.792854000 4.243289000

H 6.835741000 5.236902000 3.253630000

C 4.383261000 7.023185000 3.764183000

H 4.934203000 7.352574000 2.873551000

H 3.325030000 7.286556000 3.655812000

H 4.793460000 7.503874000 4.660022000

[Ru(NSe)]$^0$

N 1.773873000 2.069328000 5.564480000

Se 0.147449000 1.626591000 5.516518000

Cl 5.795879000 2.397544000 6.437782000

Ru 3.484440000 2.310041000 5.851306000

S 4.220063000 1.680001000 3.598726000

S 3.484488000 4.745981000 5.598265000

S 3.695037000 -0.131771000 6.112244000

S 2.841050000 2.989192000 8.152971000

P 3.467813000 3.116189000 2.347248000

P 4.540748000 5.212950000 3.901166000

P 3.401262000 -0.521862000 8.096126000

P 3.946788000 1.908840000 9.494569000

N 4.037291000 4.614713000 2.489795000

N 4.197504000 0.351524000 9.185353000

C 3.010326000 2.090677000 11.050071000

H 2.020729000 1.636564000 10.925119000

H 3.553674000 1.570794000 11.850631000

H 2.892433000 3.150498000 11.307727000

C 5.547541000 2.680105000 9.838778000

H 6.057531000 2.104252000 10.622592000

H 6.132702000 2.663628000 8.912515000

H 5.395643000 3.717019000 10.161838000

C 1.609247000 -0.538300000 8.437660000

H 1.095433000 -1.212144000 7.742148000

H 1.434626000 -0.854141000 9.475410000

H 1.233228000 0.482941000 8.295686000

C 3.964728000 -2.230874000 8.317377000

H 3.745700000 -2.552674000 9.343678000

H 3.464282000 -2.890805000 7.598973000

H 5.047217000 -2.257559000 8.148476000

C 1.653150000 3.165251000 2.433371000

H 1.284302000 3.830656000 1.641347000

H 1.223210000 2.163029000 2.327400000

H 1.377073000 3.585910000 3.406957000

C 3.868063000 2.539627000 0.673982000

H 4.955216000 2.426292000 0.592658000

H 3.382205000 1.577051000 0.475300000

H 3.526641000 3.292907000 -0.047861000

C 6.301126000 4.850983000 4.137355000

H 6.673719000 5.331344000 5.049437000

H 6.415914000 3.765692000 4.243585000

H 6.850119000 5.205763000 3.254732000

C 4.416131000 7.018419000 3.771395000

H 4.970372000 7.344620000 2.881634000

H 3.360644000 7.292904000 3.663850000

H 4.831313000 7.492392000 4.668514000

[Ru(NTe)]$^0$

N 1.738851000 2.099909000 5.555329000

Te -0.087288000 1.609287000 5.558835000

Cl 5.770401000 2.355992000 6.438738000

Ru 3.450565000 2.312684000 5.836699000

S 4.185210000 1.676045000 3.590187000

S 3.501695000 4.743939000 5.599036000

S 3.621150000 -0.131303000 6.099548000

S 2.800074000 2.982766000 8.140647000

P 3.461103000 3.128285000 2.339705000

P 4.568511000 5.198980000 3.904405000

P 3.407947000 -0.520029000 8.095717000

P 3.933340000 1.923453000 9.475426000

N 4.055082000 4.616560000 2.490252000

N 4.220184000 0.374412000 9.155649000

C 2.994126000 2.076820000 11.032895000

H 2.014326000 1.602271000 10.906912000

H 3.549369000 1.565159000 11.830589000

H 2.853261000 3.132544000 11.295859000

C 5.515795000 2.729955000 9.823362000

H 6.042433000 2.158620000 10.599395000

H 6.098398000 2.737718000 8.895387000

H 5.338398000 3.758899000 10.158744000

C 1.629595000 -0.578576000 8.498952000

H 1.108013000 -1.267390000 7.824175000

H 1.497160000 -0.892926000 9.543432000

H 1.224046000 0.432399000 8.363338000

C 4.018558000 -2.214614000 8.300031000

H 3.836745000 -2.542223000 9.331796000

H 3.513962000 -2.886184000 7.595367000

H 5.095980000 -2.214479000 8.099687000

C 1.647807000 3.205706000 2.420813000

H 1.291587000 3.888565000 1.637922000

H 1.202213000 2.212072000 2.299603000

H 1.375675000 3.614877000 3.400561000

C 3.858294000 2.550856000 0.665972000

H 4.943872000 2.421326000 0.587788000

H 3.358577000 1.596359000 0.462721000

H 3.530555000 3.311310000 -0.054711000

C 6.320868000 4.802040000 4.143693000

H 6.701468000 5.275281000 5.056221000

H 6.413748000 3.714695000 4.251128000

H 6.878629000 5.145335000 3.262043000

C 4.478315000 7.006978000 3.782597000

H 5.040513000 7.326780000 2.895510000

H 3.428433000 7.301882000 3.674209000

H 4.900375000 7.469047000 4.682723000

[Ru(NO)]$^{--1}$

N 1.960115000 2.091852000 5.729712000

O 0.829340000 2.467388000 5.880344000

Cl 6.415894000 3.117269000 6.671831000

Ru 3.683024000 2.524142000 6.112792000

S 4.650246000 1.904460000 3.820912000

S 3.203686000 4.897753000 5.652210000

S 4.454045000 0.144431000 6.684053000

S 3.014846000 3.223398000 8.380638000

P 3.660706000 3.085327000 2.511950000

P 4.208442000 5.446860000 3.961270000

P 3.260569000 -0.427911000 8.211777000

P 3.797624000 1.898784000 9.722956000

N 3.892984000 4.684425000 2.568534000

N 3.382647000 0.336923000 9.630875000

C 3.190620000 2.455968000 11.349812000

H 2.096332000 2.391283000 11.354790000

H 3.600114000 1.796907000 12.127157000

H 3.499675000 3.492334000 11.531039000

C 5.599012000 2.076410000 9.790124000

H 6.010871000 1.326281000 10.479235000

H 5.997333000 1.944613000 8.770543000

H 5.862005000 3.092482000 10.110395000

C 1.503275000 -0.435961000 7.718008000

H 1.371378000 -0.929731000 6.747996000

H 0.915095000 -0.941911000 8.496568000

H 1.172681000 0.605652000 7.641898000

C 3.642275000 -2.178731000 8.555681000

H 3.049247000 -2.514715000 9.416952000

H 3.419719000 -2.794997000 7.675986000

H 4.709349000 -2.256776000 8.794867000

C 1.863758000 2.767937000 2.556201000

H 1.390680000 3.267717000 1.699169000

H 1.655210000 1.691630000 2.542832000

H 1.471492000 3.201370000 3.482746000

C 4.172613000 2.568549000 0.838800000

H 5.261898000 2.669424000 0.766516000

H 3.890569000 1.523341000 0.662344000

H 3.695068000 3.223186000 0.097375000

C 5.990992000 5.475925000 4.280413000

H 6.216966000 6.202576000 5.070990000

H 6.290681000 4.483766000 4.656537000

H 6.520292000 5.726413000 3.350732000

C 3.735488000 7.183980000 3.670535000

H 4.265130000 7.552744000 2.781825000

H 2.654146000 7.226699000 3.495727000

H 3.992195000 7.794486000 4.544502000

[Ru(NS)]$^{--1}$

N 2.083658000 2.317296000 5.756090000

S 0.646906000 2.923154000 5.972532000

Cl 6.291659000 2.935060000 6.564570000

Ru 3.860526000 2.541957000 6.149723000

S 4.653720000 1.871464000 3.885094000

S 3.574259000 4.940482000 5.855025000

S 4.343431000 0.145563000 6.557344000

S 3.258896000 3.212968000 8.416723000

P 3.552877000 2.934244000 2.568134000

P 4.186220000 5.331060000 3.960469000

P 3.166448000 -0.403398000 8.108395000

P 3.959193000 1.789656000 9.690041000

N 3.510799000 4.541854000 2.722050000

N 3.411343000 0.272944000 9.557575000

C 3.459690000 2.338070000 11.355897000

H 2.365175000 2.389256000 11.390432000

H 3.815347000 1.607007000 12.094352000

H 3.881146000 3.327886000 11.568403000

C 5.774338000 1.792012000 9.713489000

H 6.124671000 1.022091000 10.414943000

H 6.122957000 1.581287000 8.692356000

H 6.148664000 2.783918000 9.994884000

C 1.401938000 -0.249542000 7.686919000

H 1.193633000 -0.699591000 6.709213000

H 0.802457000 -0.726936000 8.474877000

H 1.158546000 0.818342000 7.642117000

C 3.426499000 -2.193988000 8.347555000

H 2.838504000 -2.529605000 9.212441000

H 3.126355000 -2.744030000 7.447274000

H 4.491674000 -2.365323000 8.542433000

C 1.837360000 2.336433000 2.451061000

H 1.313334000 2.895528000 1.662993000

H 1.830868000 1.261705000 2.230670000

H 1.342522000 2.508061000 3.413956000

C 4.247431000 2.563024000 0.915806000

H 5.298592000 2.874518000 0.907307000

H 4.184849000 1.487965000 0.705320000

H 3.692228000 3.129996000 0.155838000

C 6.004573000 5.243204000 3.900530000

H 6.431594000 5.986374000 4.585704000

H 6.302819000 4.245831000 4.252625000

H 6.351615000 5.414981000 2.871955000

C 3.781113000 7.082299000 3.657604000

H 4.163015000 7.378219000 2.671344000

H 2.690280000 7.189168000 3.674504000

H 4.224196000 7.709618000 4.440316000

[Ru(NSe)]$^{--1}$

N 2.108558000 2.340547000 5.758722000

Se 0.542220000 3.066818000 6.008760000

Cl 6.314212000 2.925173000 6.567828000

Ru 3.868805000 2.555569000 6.160114000

S 4.673967000 1.882123000 3.896898000

S 3.619185000 4.956691000 5.879765000

S 4.342676000 0.155738000 6.563509000

S 3.284991000 3.231984000 8.426963000

P 3.552121000 2.926559000 2.581876000

P 4.178820000 5.331028000 3.965088000

P 3.152947000 -0.396228000 8.105071000

P 3.960317000 1.789068000 9.693308000

N 3.470798000 4.530780000 2.753171000

N 3.384737000 0.284007000 9.554413000

C 3.468364000 2.340394000 11.359850000

H 2.374760000 2.408151000 11.393726000

H 3.812485000 1.601938000 12.096370000

H 3.904758000 3.323075000 11.575166000

C 5.775460000 1.763461000 9.715027000

H 6.115014000 0.982737000 10.409759000

H 6.120481000 1.555925000 8.692034000

H 6.164557000 2.747307000 10.004614000

C 1.393796000 -0.250306000 7.666224000

H 1.194528000 -0.721719000 6.696662000

H 0.786516000 -0.709982000 8.458668000

H 1.150294000 0.816420000 7.593088000

C 3.420518000 -2.185375000 8.346855000

H 2.823988000 -2.524117000 9.204657000

H 3.133842000 -2.737238000 7.443331000

H 4.484212000 -2.350765000 8.554564000

C 1.854687000 2.286102000 2.453984000

H 1.307122000 2.857883000 1.691647000

H 1.878712000 1.220410000 2.194047000

H 1.365315000 2.408320000 3.427716000

C 4.266537000 2.584459000 0.931375000

H 5.307525000 2.928590000 0.929271000

H 4.238684000 1.509002000 0.715494000

H 3.697394000 3.137071000 0.171115000

C 5.996430000 5.244581000 3.866933000

H 6.435303000 6.002840000 4.527781000

H 6.303688000 4.254792000 4.232271000

H 6.322980000 5.396463000 2.828562000

C 3.762278000 7.078622000 3.659943000

H 4.125958000 7.370934000 2.665761000

H 2.671427000 7.180353000 3.695192000

H 4.215952000 7.711895000 4.431710000

[Ru(NTe)]$^{--1}$

N 2.157639000 2.402570000 5.753531000

Te 0.435863000 3.293934000 6.004296000

Cl 6.368031000 2.888102000 6.581364000

Ru 3.910032000 2.570524000 6.179081000

S 4.735489000 1.901884000 3.920937000

S 3.724834000 4.976918000 5.933942000

S 4.329964000 0.160531000 6.558334000

S 3.333071000 3.243485000 8.445391000

P 3.563990000 2.903759000 2.615915000

P 4.155626000 5.327295000 3.980504000

P 3.127749000 -0.381048000 8.095057000

P 3.976255000 1.775303000 9.702321000

N 3.388601000 4.495109000 2.828702000

N 3.369991000 0.284662000 9.549587000

C 3.491017000 2.325261000 11.370863000

H 2.398965000 2.415469000 11.402050000

H 3.817444000 1.574476000 12.102924000

H 3.947041000 3.297045000 11.594674000

C 5.790307000 1.717067000 9.724633000

H 6.115391000 0.924191000 10.412407000

H 6.133488000 1.513583000 8.700109000

H 6.196290000 2.691156000 10.023944000

C 1.374490000 -0.198877000 7.651977000

H 1.167056000 -0.666707000 6.682474000

H 0.753868000 -0.641979000 8.443400000

H 1.154927000 0.873283000 7.575432000

C 3.365107000 -2.175951000 8.322961000

H 2.759420000 -2.511611000 9.175517000

H 3.072494000 -2.715426000 7.413897000

H 4.424994000 -2.361158000 8.533378000

C 1.910804000 2.166104000 2.459204000

H 1.313794000 2.749562000 1.744761000

H 2.003614000 1.124463000 2.126909000

H 1.431444000 2.191274000 3.445390000

C 4.312951000 2.636169000 0.965833000

H 5.327920000 3.050733000 0.976208000

H 4.361233000 1.564722000 0.733857000

H 3.712332000 3.158950000 0.208627000

C 5.968726000 5.265960000 3.791328000

H 6.425375000 6.050124000 4.408608000

H 6.308682000 4.290602000 4.165714000

H 6.244609000 5.394606000 2.735173000

C 3.692641000 7.061892000 3.673322000

H 4.007035000 7.350072000 2.661301000

H 2.602363000 7.140434000 3.754905000

H 4.164960000 7.713565000 4.418142000
